# The causal relationship between sarcopenic obesity factors and benign prostate hyperplasia

**DOI:** 10.3389/fendo.2023.1290639

**Published:** 2023-11-08

**Authors:** Xuezhi Rao, Zhijie Xu, Jingchun Zhang, Jiaxiang Zhou, Jian Huang, Zhanhao Toh, Ruwen Zheng, Zhiyu Zhou

**Affiliations:** ^1^ Beijing University of Chinese Medicine, Beijing, China; ^2^ The Second School of Clinical Medicine, Beijing University of Chinese Medicine, Beijing, China; ^3^ Graduate School, Beijing University of Chinese Medicine, Beijing, China; ^4^ Xiyuan Hospital, China Academy of Chinese Medical Sciences, Beijing, China; ^5^ Department of Spinal Surgery, The Affiliated Hospital of Qingdao University, Qingdao, China; ^6^ Department of Acupuncture and Moxibustion, Dongfang Hospital, Beijing University of Chinese Medicine, Beijing, China; ^7^ Singa Care Medical, Singapore, Singapore; ^8^ Innovation Platform of Regeneration and Repair of Spinal Cord and Nerve Injury, Department of Orthopaedic Surgery, The Seventh Affiliated Hospital, Sun Yat-Sen University, Shenzhen, China; ^9^ Guangdong Provincial Key Laboratory of Orthopedics and Traumatology, Orthopaedic Research Institute/Department of Spinal Surgery, The First Affiliated Hospital of Sun Yat-Sen University, Guangzhou, China

**Keywords:** sarcopenic obesity, basal metabolic rate, appendicular lean mass, benign prostatic hyperplasia, genome-wide association study, mendelian randomization

## Abstract

**Background:**

Both benign prostatic hyperplasia (BPH) and sarcopenic obesity (SO) are common conditions among older adult/adults males. The prevalent lifestyle associated with SO is a significant risk factor for the development of BPH. Therefore, we investigated the causal relationship between SO factors and BPH.

**Method:**

The instrumental variables for SO factors were selected using the inverse variance-weighted method, which served as the primary approach for Mendelian randomization analysis to assess the causal effect based on summary data derived from genome-wide association studies of BPH.

**Result:**

The increase in BMR (OR = 1.248; 95% CI = (1.087, 1.432); P = 0.002) and ALM (OR = 1.126; 95% CI = (1.032, 1.228); P = 0.008) was found to be associated with an elevated risk of BPH. However, no genetic causality between fat-free mass distribution, muscle mass distribution, and BPH was observed.

**Conclusion:**

Our findings indicate that a genetic causal association between BMR, ALM and BPH. BMR and ALM are risk factors for BPH. The decrease in BMR and ALM signified the onset and progression of SO, thus SO is a protective factor for BPH.

## Introduction

With the increasing global aging population (1), the prevalence of benign prostatic hyperplasia (BPH) is resulting in a significant economic burden. Consequently, the prevention and treatment of BPH will pose a major challenge in the future (2). Several Mendelian randomization studies have consistently demonstrated that waist circumference, sedentary behavior (3), thyroid disorders (4), higher education level (5), and bioavailable testosterone (6) are risk factors for BPH.

Obesity plays a crucial and precarious role in the pathogenesis of BPH (7–12). However, previous studies have indicated a lack of causal relationship between body mass index (BMI) and BPH (3). This contradicts the findings from existing clinical trial observations (13). Disparities in this context may arise from varying prevalence of distinct obesity subtypes in diverse research efforts (14). BMI, while reflecting overall obesity, obscures nuances like abdominal or sarcopenic obesity (SO), hindering differentiation of obesity types statistically. Recognizing these varied obesity profiles is crucial due to their differing risks in BPH development. Waist circumference (WC) plays a pivotal role in diagnosing abdominal obesity, closely related to prostate volume and IPSS scores (15, 16), as affirmed in recent MR studies. Relying solely on BMI obscures understanding of obesity’s interplay with BPH, differing from observational trials and MR findings. Adoption of representative obesity subtype markers is thus vital. The correlation between WC and BPH is supported by observational studies and MR research (3), highlighting SO’s substantial impact on older individuals’ lives. In order to gain a deeper comprehension of the relationship between obesity and BPH, it is necessary to do further research into the correlation between SO and BPH. BPH is an age-related condition, with a higher incidence observed among the older adult/adults population, 50% of men over the age of 50 shown to have evidence of BPH, BPH prevalence comes to 80% in those over 70 years (7). Among the older adult/adults population, the most prevalent form of obesity is SO (17, 18). Addressing SO (19) and BPH (1) are important for preventing longterm disability in the older adults at high risk. To better understand the causal relationship between SO and BPH, we used SO-related indices that have not been previously studied. Previous investigations have suggested that increased waist circumference (WC) is a risk factor for BPH (3), which is a trait related to SO. In this study, we focused on analyzing indices related to SO, which is characterized by both obesity and decreased muscle mass (20). Basal metabolic rate (BMR) and appendicular lean mass (ALM) are factors associated with SO (21), the decrease in BMR and ALM signifies the onset and progression of SO. Since there is no direct measure of appendicular skeletal muscle mass index (ASMI) traits, ALM can serve as a main substitute to assess muscle mass and reflect the degree of muscle atrophy (22).

In this current study, we aimed to investigate the impact of SO on BPH by assessing the causal relationship between BMR, ALM, and BPH. Furthermore, we sought to explore the potential role of fat-free mass (FFM) distribution and muscle mass (MM) distribution as risk factors in the development of BPH.

## Materials and methods

**Figure 1:**
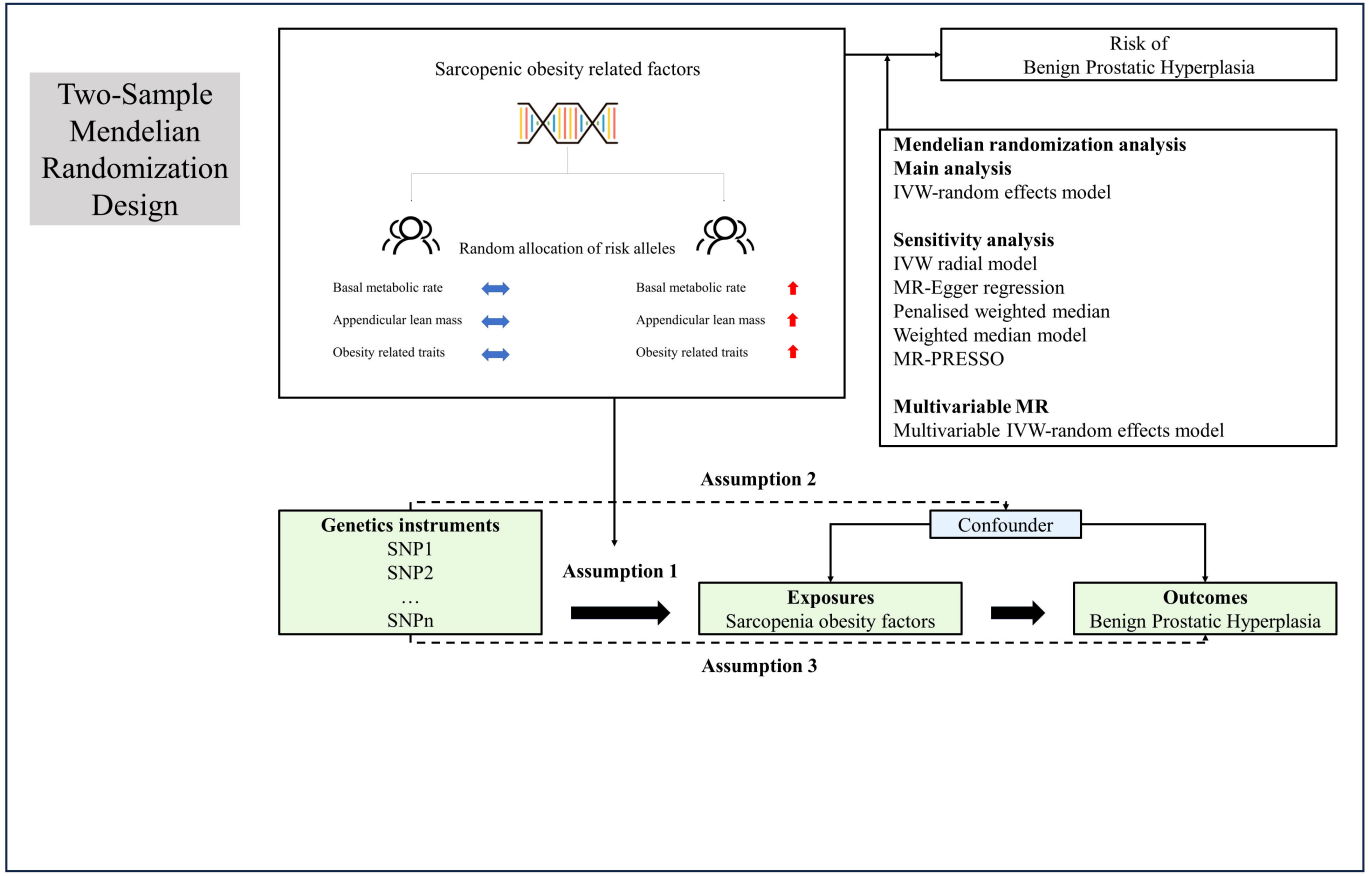
Overview and assumptions of the Mendelian randomization study design. Assumption 1: the instrumental variables should be closely related to the risk factor of interest; assumption 2: the instrumental variables should not be associated with potential confounders, and assumption 3: the instrumental variables should affect the risk of outcome only through risk factors and not through other alternative pathways. LD, Linkage disequilibrium; SNP, single nucleotide polymorphisms; IVW, inverse-variance weighted; PRESSO, Pleiotropy Residual Sum and Outlier.

**Figure 1** presented the study design and the assumptions of Mendelian randomization (MR) in our study. We performed two-sample univariable and multivariable MR based on the previous Epidemiology study. This IV analysis mimics randomized controlled trial with respect to the random allocation of single nucleotide polymorphisms (SNPs) in offspring (independent of confounding factors such as sex and age). The data used in this study were obtained from recent genome-wide association studies (GWAS). Specifically, we extracted the data from the IEU OpenGWAS database, which was developed by the MRC Integrative Epidemiology Unit (IEU) at the University of Bristol (23, 24). This database offered a comprehensive collection of GWAS summary datasets that have been meticulously curated. Users can gain access to the platform by visiting the website https://gwas.mrcieu.ac.uk. In this study, we focused on BMR, ALM, and sixteen other traits that might influence the occurrence of BPH, including body fat percentage (BFP), whole body fat mass (WBFM), whole body fat-free mass (WBFFM), whole body water mass (WBWM), leg fat percentage (LFP), leg fat mass (LFM), leg fat-free mass (LFFM), leg predicted mass (LPM), arm fat percentage (AFP), arm fat mass (AFM), arm fat-free mass (AFFM), arm predicted mass (APM), trunk fat percentage (TFP), trunk fat mass (TFM), trunk fat-free mass (TFFM) and trunk predicted mass (TPM).

In this investigation, we scrutinized the following traits: BMR, ALM, Obesity factors and BPH. GWAS evaluations were undertaken in contributors of European lineage, predicated upon K-means clustering (K = 4), subsequent to routine exclusions, that include withdrawn consent, suspected sex chromosome aneuploidy, and discordance between genetically inferred and self-reported sex (25). Appropriate IVs for the MR evaluations were culled from disparate GWAS summary findings. SNPs that met the rigorous criterion of genome-wide significance (p < 5 × 10^−8^) were selected during the initial phase. Subsequently, relevant SNPs were retained based on the linkage disequilibrium criterion, stipulated by an R² < 0.001 according to the Genome reference panel (26). SNPs exhibiting an association with the outcome variables at a significance level of p < 5 × 10^−8^ were methodically excluded from consideration. Variants signifying correlations with BMR, ALM, Obesity factors and BPH, meeting conventional GWAS thresholds (P < 5 × 10^−8^), were harnessed to fabricate genetic instruments tailored to each phenotype. A genetic instrument embodies one or numerous genetic variances imbued with attributes conducive to their utilization as an IV within the purview of MR (27). Throughout the harmonization process encompassing both exposure and outcome data sets, palindromic SNPs and those devoid of requisite information were meticulously purged. The robustness of the IVs was evaluated via the computation of F-statistics, with values beneath the threshold of 10 indicating an inherently weak instrument strength, thereby necessitating their removal from the analysis (28, 29).

### Data sources of phenotypes

The BMR and ALM metrics were derived from the UK Biobank (UKB) dataset, which included a sample size of 331,307 individuals for BMR and 205,513 individuals for ALM. Regarding the BPH phenotype, we utilized participants from the FinnGen, comprising 13,118 cases and 72,799 controls. For more comprehensive information on all the included phenotypes, please refer to **Supplementary Tables 1**–**3**.

### Statistical analysis

**Figure 2:**
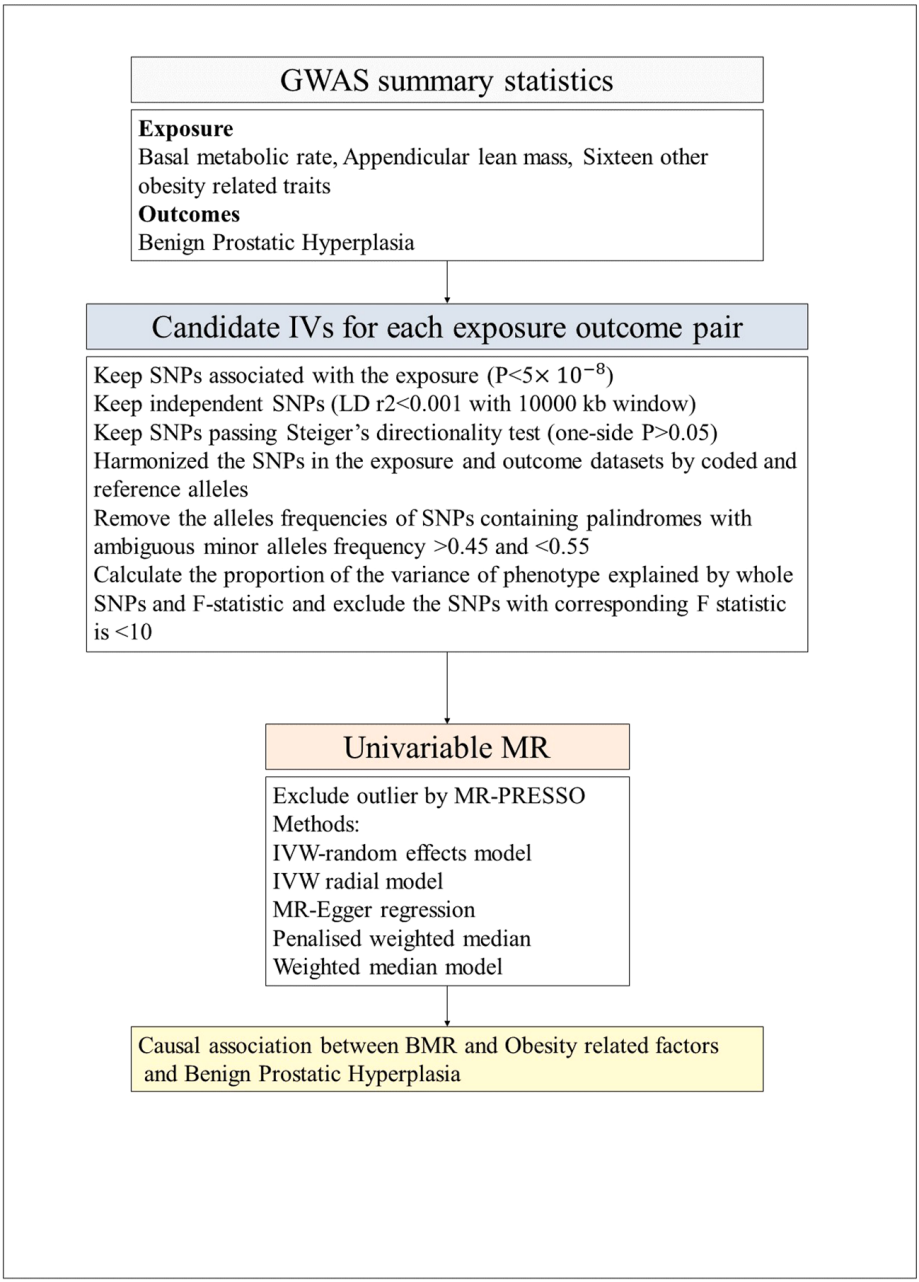
The flow chart of the inclusion and exclusion criterion of candidate SNPs for each exposure-outcome pair. GWAS, genome-wide association studies; LD, Linkage disequilibrium; IVW, inverse-variance weighted; PRESSO, Pleiotropy Residual Sum and Outlier; MR, Mendelian randomization; BMR, Basal metabolic rate.

The flow chart of our study, as depicted in **Figure 2**, outlines the inclusion and exclusion criteria for candidate SNPs in each exposure-outcome pair. To perform the analysis, we employed the TwoSample MR and MR-PRESSO packages in R software (version 4.3.1). We employed a P value threshold of 5 × 10^–8^ to identify significant SNPs for each exposure variable. To ensure independence and significance, we performed linkage disequilibrium (LD)-based clumping with parameters R^2^ < 0.001 and kb = 10,000 (30). These selected SNPs were then utilized for the outcome variable to calculate the effect size of each SNP. Finally, we harmonized both the exposure data and the outcome data to facilitate subsequent MR analysis.

A two-sample MR approach was employed to assess the association between BMR, obesity-related factors and BPH. The impact of exposure on the outcome can be estimated by calculating the ratio between the genetic outcome and genetic exposure associations. Moreover, if the genetic variants (GVs) are not in linkage disequilibrium, their ratio estimates can be combined using inverse variance weighted (IVW) methods (31) to obtain a comprehensive estimate. Causality assessment primarily relied on IVW methods when all SNPs met instrumental variable requirements. Additionally, MR-Egger regression, weighted median, weighted mode, and simple mode analyses were used for supplementary evaluation in cases involving outliers (32).

If there were no weak instrumental variables (IVs), we utilized the IVW method as the primary outcome, while considering the other methods as secondary outcomes (33). In case of significant pleiotropy detected by the MR-PRESSO method, we would address this issue by removing outlier variability and repeating the MR analysis (34). The leave-one-out test was employed to assess individual SNP effects (35). Heterogeneity was evaluated using Cochran’s Q test, with a significance level of P < 0.05 indicating substantial heterogeneity and necessitating SNP exclusion (35). The MR-Egger method provided estimates of horizontal pleiotropy through intercepts from linear regressions between SNP outcome and SNP exposure associations (36). A nominal significance level of p < 0.05 indicated statistical significance (37).

We employed multivariable MR as a statistical methodology that enables the incorporation of SNP-phenotype associations into the analysis, facilitating estimation of the direct impact of each phenotype on the outcome (38). To account for potential unbalanced horizontal pleiotropy in our analysis, we also conducted multivariable MR-Egger analyses (39).

## Results

**Figure 3:**
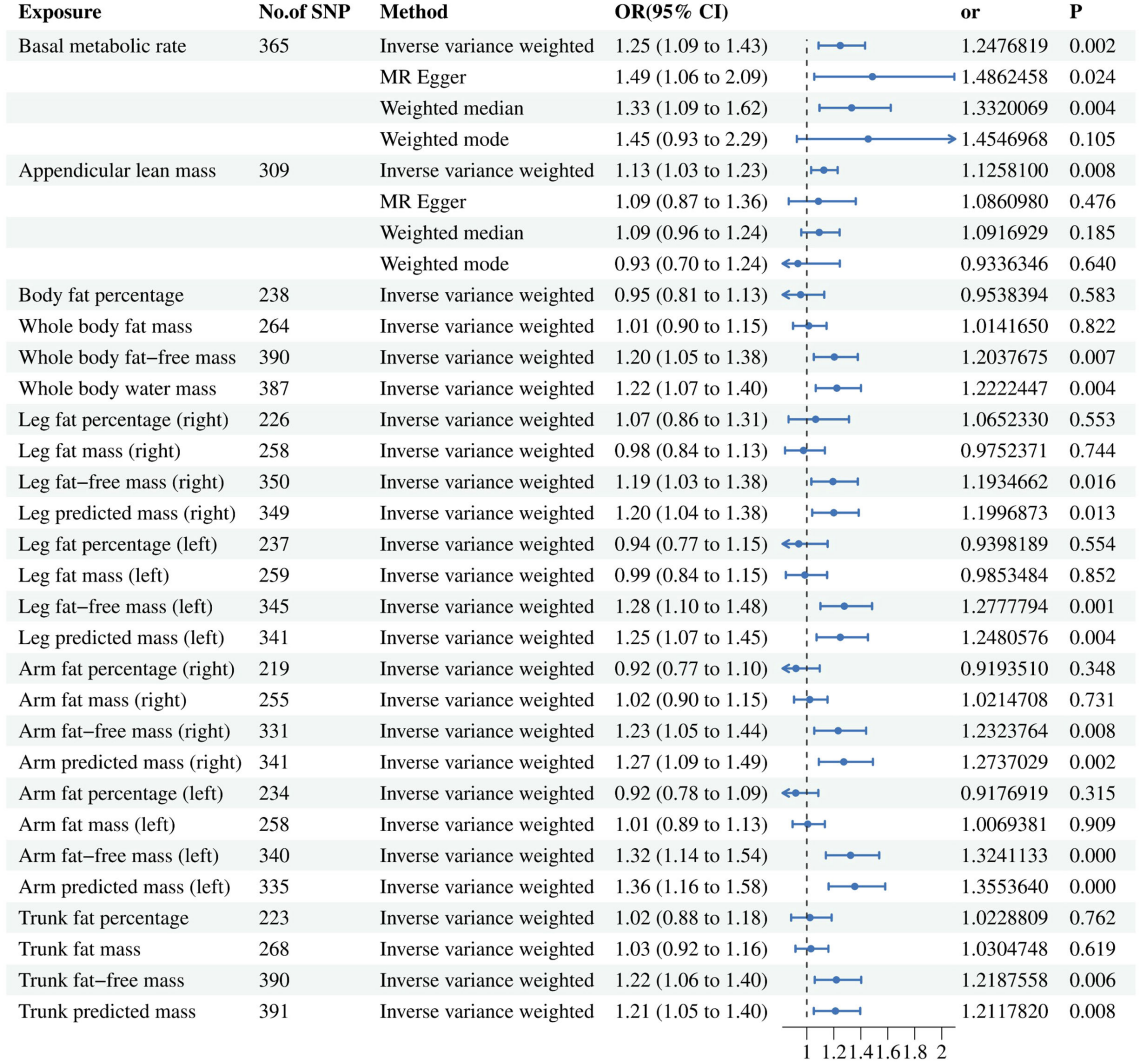
Associations of genetically predicted risk factors with benign prostatic hyperplasia using random effect inverse-variance weighted method. IVW, inverse-variance weighted; OR, odds ratio; CL, confidence interval; SNP, single nucleotide polymorphism.

The UVMR results are presented in **Figure 3**. A total of 365 SNPs related to BMR and 309 SNPs related to ALM were ultimately selected for evaluating their contributions to BPH outcome. Details of significant SNPs and relevant information for other traits can be found in **Supplementary Tables 1**, **2**. All the SNPs exhibited sufficient strength (mean F-statistic > 10) and demonstrated the correct causal direction.

After conducting a comprehensive meta-regression analysis, we made a significant observation that an increase in BMR (ORIVW = 1.248; 95% CI = (1.087, 1.432); P = 0.002) and ALM (ORIVW = 1.126; 95% CI = (1.032, 1.228); P = 0.008) is associated with an elevated risk of developing BPH. Furthermore, even after removing outliers through the MR-PRESSO analysis, the association between BMR and ALM with BPH remained robust as indicated by non-significant distortion test results (P > 0.05). However, it should be noted that no statistically significant relationship was observed between ALM and BPH using the other three methods. In the context of more precise muscle mass and fat distribution, the fat-free mass related factors have causal relationship with BPH, while the fat-percentage factors have not.

**Figure 4:**
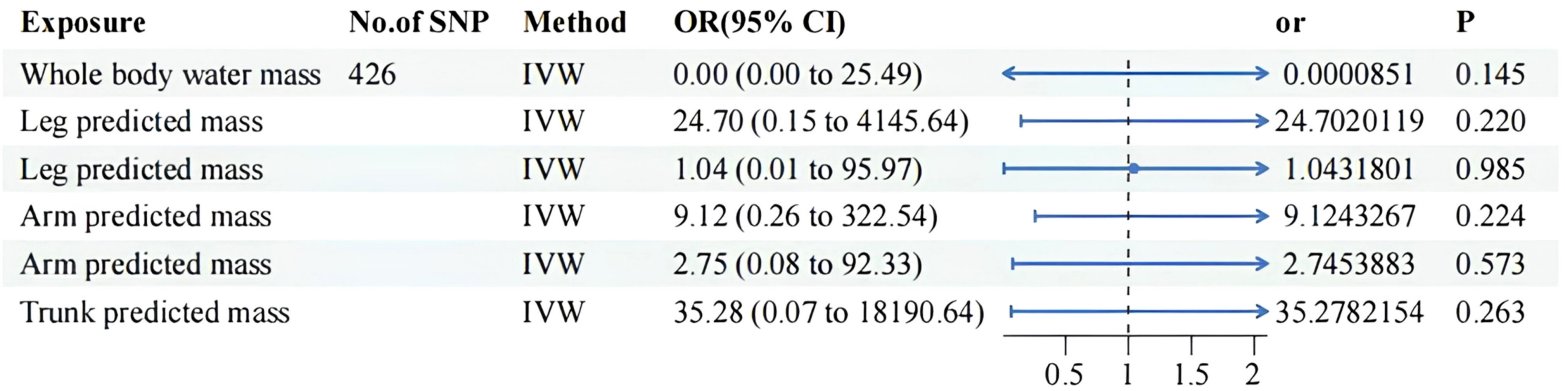
MVMR result of predicted mass related factors using random effect inverse-variance weighted method. IVW, inverse-variance weighted; OR, odds ratio; CI confidence interval; SNP, single nucleotide polymorphism.

**Figure 5:**
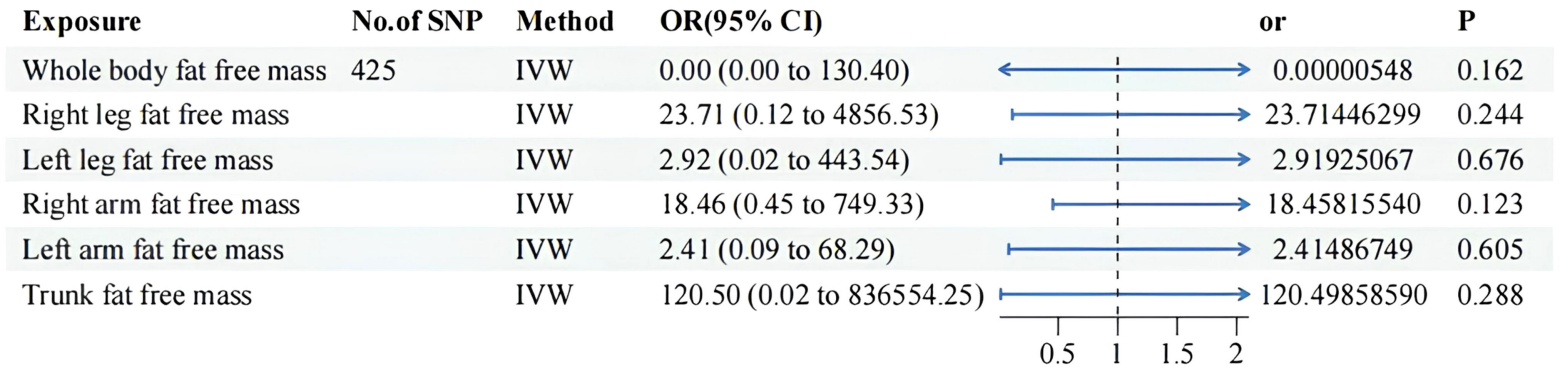
MVMR result of fat free mass related factors using random effect inverse-variance weighted method. IVW, inverse-variance weighted; OR, odds ratio; CI confidence interval; SNP, single nucleotide polymorphism.

Details of significant SNPs and relevant information for MVMR traits can be found in **Supplementary Tables 4**-**6**. All the SNPs exhibited sufficient strength (mean F-statistic > 10) and demonstrated the correct causal direction. All MVMR results were listed in **Figures 4** and **5**. The MVMR analysis did not reveal any genetic causal associations. The genetic causality between the distribution of FFM, MM, and BPH was not observed. The leave-one-out sensitivity analysis, funnel plots, scatter diagram and forest map can be fund from **Supplementary Figure 3-1** to **Supplementary Figure 3-4**. None horizontal pleiotropy were found. Heterogeneity were found in MVMR, the heterogeneity test result can be found in **Supplementary Table 8**.

## Discussion

To the best of our knowledge, hitherto no MR studies have embarked upon an exploration of the causal relationship between SO and BPH. Our study used the MR approach to identify the genetic causal association between BMR, ALM and BPH, indicating that BMR and ALM serves as a significant risk factor for the development of BPH. Each component of body fat-free mass may contribute to an increased susceptibility to BPH. However, no substantial evidence was observed in this study to suggest a causal relationship between fat mass and BPH. Most of the research observed BMR and ALM decreased in SO (40). Our MR results suggested the increase of BMR and ALM would rise the risk of occurring BPH. Our results indicated that the SO is a protective factor to BPH.

In an observational study, they found that an elevation in BMR significantly elevated the risk of developing BPH (41), which were consistent with our MR results. BMR is widely utilized as an index for measuring the basal metabolic rate in human bodies (42) and finds extensive application in studies related to obesity, aging, and other relevant fields.

BMR serves as a crucial indicator for predicting the progression of SO in individuals (20). The reported prevalence of SO among adults is 3.40% (21). A decrease in BMR heightens the risk of developing SO (21) and often indicates an increased proportion of visceral fat (21). In addition to being obese, patients with SO commonly exhibit sedentary behaviors, prolonged periods of sitting or bed rest, and an increase in waist circumference. Some individuals within this group may even present with Metabolic Syndrome (MetS) (11). All these factors collectively contribute to the risk factors associated with the onset of BPH. According to our research, it is evident that patients with SO are less likely to developing BPH. Normally, with age advancement, there is a tendency for BMR to decrease (20). Within the context of obesity-related disorders, divergent perspectives arise. A study indicates that as weight increases, BMR gradually rises (43). However, other studies suggested that obese individuals have a lower BMR compared to normal individuals (44, 45). It is important to note that these two observational outcomes may not contradict each other. As weight increases, BMR rises (46); however, in cases where weight is comparable, the BMR of obese patients is lower compared to the normal population (47). This can be attributed to the muscle-fat ratio in obese individuals and alterations in their muscle type (48).

BMR is regulated by a complex network of hormones and metabolic pathways. Both testosterone (49) and thyroid (50) hormones have the ability to increase BMR. In populations with obesity (51), aging, and MetS (52), there were a significant decrease in BMR. The value of BMR is determined by the intricate interplay of various factors. Bioavailable testosterone (6), thyroid hormones (4), obesity (7), aging, and MetS (52) all act as risk factors for the development of BPH. This highlights the complexity of BMR in the pathogenesis of BPH. These aforementioned factors serve as potential mediators through which BMR may influence the onset of BPH. Reduced bioavailable testosterone lowers BMR, increasing obesity risk. Conversely, elevated bioavailable testosterone is a key contributor to BPH onset. Low thyroid hormone levels decrease BMR, enhancing obesity risk (53), while high levels increase BPH risk (4). Those with obesity often show increased BMR (54), often mirroring their weight progression. Numerous studies identify obesity as a significant BPH risk factor. Elderly obesity rates are rising, largely due to chronic diseases and metabolic imbalances. In the general population, BMR decreases with age, but BPH incidence grows (17). The rise in metabolic disturbances in aging may explain increased older adult/adults obesity (53). Intriguingly, those diagnosed with metabolic syndrome consistently register a BMR inferior to their counterparts (55), with BMR exhibiting a profound association with metabolic age (52); an ascendant BMR frequently heralds a rejuvenated metabolic age.

Among various factors, only age concurrently elevates the risk for both obesity and BPH. This suggests that while both SO and BPH predominantly afflict older adult/adults males, there exists a dichotomous relationship between them. Clinically, SO patients without an increase in waist circumference and BMI present a diminished risk of BPH and are less likely to experience urinary obstruction.

In clinical diagnostics, uroflowmetry is a guideline-recommended pivotal tool for evaluating BPH obstructive symptoms, with the severity of these symptoms dictating the need for surgical intervention (56). With the advancement of portable home devices, the acquisition of UF data has become increasingly accessible and cost-effective (57). Comprehensive UF information aids in the precise assessment of LUTS symptoms. An enlarged WR poses a risk factor for SO (3, 58). An increase in WR is correlated with BPH obstructive symptoms, impacting the frequency score of the IPSS and Qmax in BPH patients (56).

BPH surgery techniques are tailored to prostate volume. Small prostates often utilize methods like HoLEP and TURP, while larger ones may opt for Simple Prostatectomy or ThuLEP (59). Though TURP is prevalent, HoLEP and ThuLEP are highly recommended. Metabolic Syndrome can affect TURP’s efficacy, and abdominal obesity correlates with better life quality (60). ThuLEP is preferable for obese individuals (61). For open surgeries, RASP provides benefits in safety and efficiency (62–64). Our studies show that while SO acts as a BPH deterrent, increased waist circumference, and rising older adult/adults obesity rates necessitate more vigilant BPH tracking and proactive interventions.

For patients undergoing prolonged bed rest, BMR constitutes the primary energy expenditure (65). Androgen replacement therapy is commonly employed to attenuate the progression of muscle atrophy (49). However, it should be noted that androgen replacement therapy may result in an elevation of BMR (49), thereby increasing the risk of BPH. Additionally, some scholars utilize thyroid hormone analogs to intervene in patients’ BMR (50). In clinical practice, regular monitoring of prostate volume and IPSS scores were advisable when utilizing androgen or thyroid hormone analogs for patient intervention. This facilitates timely adjustments in therapy strategies to prevent treatment induced BPH. For bedridden patients, the presence of BPH significantly increases the risk of urinary obstruction (66). Consequently, urinary catheterization is often necessary to facilitate urination in such cases. However, this introduces complexities in caregiving and elevates the risk of infections. Therefore, effective risk management for BPH among bedridden patients becomes imperative.

We have also identified a causal association between ALM and BPH. ALM serves as a direct indicator of muscle atrophy (22). In individuals with abdominal obesity, there is a positive correlation between ALM and waist circumference (67). Lower levels of ALM often indicate an increased risk of disability occurrence and poorer physical function (68). Physical activities unrelated to exercise intensity can also contribute to the enhancement of ALM (69). The relationship between ALM and fat remains uncertain, with conflicting reports on their association (69, 70). Current research suggests that ALM is closely linked to basal metabolic rate (71). Further investigation is required to elucidate the involvement of ALM in the pathogenesis of BPH.

Observational studies are susceptible to the influence of confounding factors, which may compromise their internal validity. To address this limitation, MR has emerged as a widely used alternative approach. MR utilizes GVs that are randomly allocated at conception, thereby minimizing the impact of confounding factors (72). Currently, there is a lack of MR studies specifically investigating the causal relationship between SO factors and BPH.

This study has several limitations. Firstly, a portion of the BPH cases in this study originated from self-reported patients in the UKB. Self-reported disease conditions might overlook some BPH cases and mistakenly include other lower urinary tract disorders. Secondly, our findings are only applicable to individuals of European lineage, and the impact of BPH in other lineages remains unexplored. Additionally, due to the lack of distinction between male and female cohorts in the UKB database of SO factors, this study encounters gender-related bias. If this missing data segment could be supplemented in future studies, it would enable more precise causal inferences and better control over potential confounding factors. Additionally, due to limitations in the database, we were unable to assess the health status of the sample population, potentially introducing latent confounding bias. Furthermore, this study employed SO factors-related genetic variants as instrumental variables to establish a causal relationship between SO factors and BPH. With limited number and strength of genetic instrument SNPs, some of the conclusions reached in this study should be cautiously interpreted. Currently, there is no existing research exploring the potential mechanisms underlying the association between SO and BPH. Evaluating the role of SO in BPH from a genetic perspective may only provide partial insights into its impact within this context.

## Conclusion

In summary, our findings suggest a genetic causal association between BMR, ALM and BPH. However, no genetic causal relationship was observed between FFM distribution, MM distribution, and BPH. The decrease in BMR and ALM signifies the onset and progression of SO. Which means SO might be a protective factor for BPH.

## Data availability statement

The original contributions presented in the study are included in the article/**Supplementary Material**. Further inquiries can be directed to the corresponding authors.

## Author contributions

XR: Conceptualization, Data curation, Formal Analysis, Funding acquisition, Investigation, Methodology, Visualization, Writing – original draft, Writing – review & editing. ZX: Conceptualization, Investigation, Methodology, Software, Visualization, Writing – original draft, Writing – review & editing. JCZ: Formal Analysis, Writing – review & editing. JXZ: Investigation, Methodology, Writing – review & editing. JH: Investigation, Writing – review & editing. ZT: Writing – review & editing. RZ: Conceptualization, Data curation, Investigation, Writing – review & editing. ZZ: Conceptualization, Investigation, Methodology, Supervision, Writing – review & editing.

## Supplementary material

Supplementary Figure 1 BMR related diagrams.

Supplementary Figure 2 ALM related diagrams.

Supplementary Figure 3 1 MR leave-one-out sensitivity analysis for other factors. 2 Funnel plots for other factors. 3 Scatter diagram for other factors. 4 Forest map for other factors.

Supplementary Table 1 SNPs of BMR for BPH in TSMR.

Supplementary Table 2 SNPs of ALM for BPH in TSMR.

Supplementary Table 3 SNPs of other related factors for BPH in TSMR.

Supplementary Table 4 The SNPs selected for other obesity factors to perform Multivariable MR analysis.

Supplementary Table 5 The SNPs selected for predicted mass related factors to perform Multivariable MR analysis.

Supplementary Table 6 The SNPs selected for fat free mass related factors to perform Multivariable MR analysis.

## Glossary

**Table:**

MR	Mendelian randomization
BMI	body mass index
SO	sarcopenic obesity
BPH	benign prostatic hyperplasia
WC	waist circumference
BMR	basal metabolic rate
ALM	appendicular lean mass
ASMI	appendicular skeletal muscle mass index
FFM	fat-free mass
MM	muscle mass
GWAS	genome-wide association studies
IEU	Epidemiology Unit
BFP	body fat percentage
WBFM	whole body fat mass
WBFFM	whole body fat-free mass
WBWM	whole body water mass
LFP	leg fat percentage
LFM	leg fat mass
LFFM	leg fat-free mass
LPM	leg predicted mass
AFP	arm fat percentage
AFM	arm fat mass
AFFM	arm fat-free mass
APM	arm predicted mass
TFP	trunk fat percentage
TFM	trunk fat mass
TFFM	trunk fat-free mass
TPM	trunk predicted mass
LD	linkage disequilibrium
IVW	inverse variance weighted
MetS	Metabolic Syndrome
GVs	genetic variants.
