# Supplementary figures and images for: The causal relationship between sarcopenic obesity factors and benign prostate hyperplasia
Source: Front Endocrinol (Lausanne). 2023 Nov 8;14:1290639. doi: 10.3389/fendo.2023.1290639 (PMC10663947; doi:10.3389/fendo.2023.1290639)

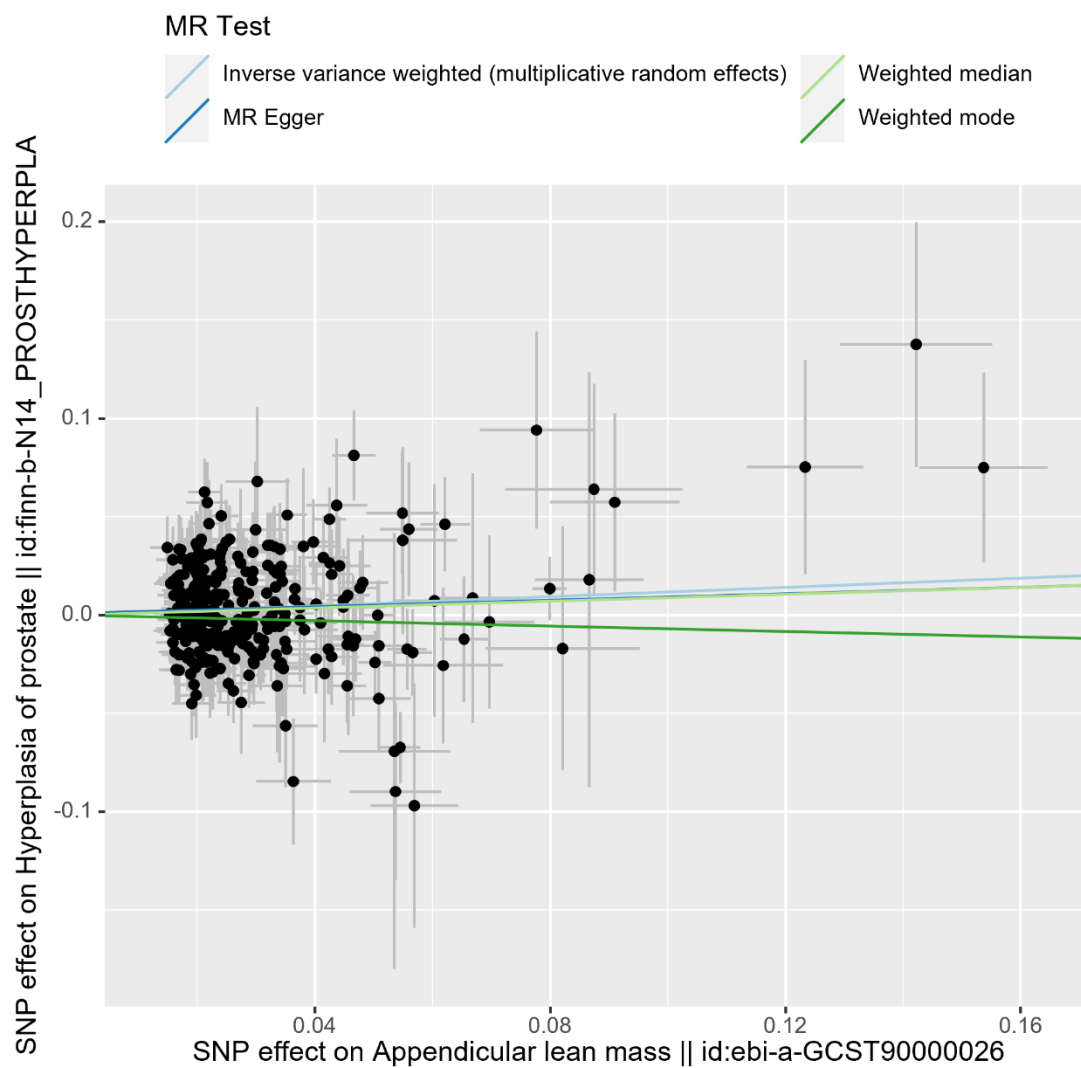

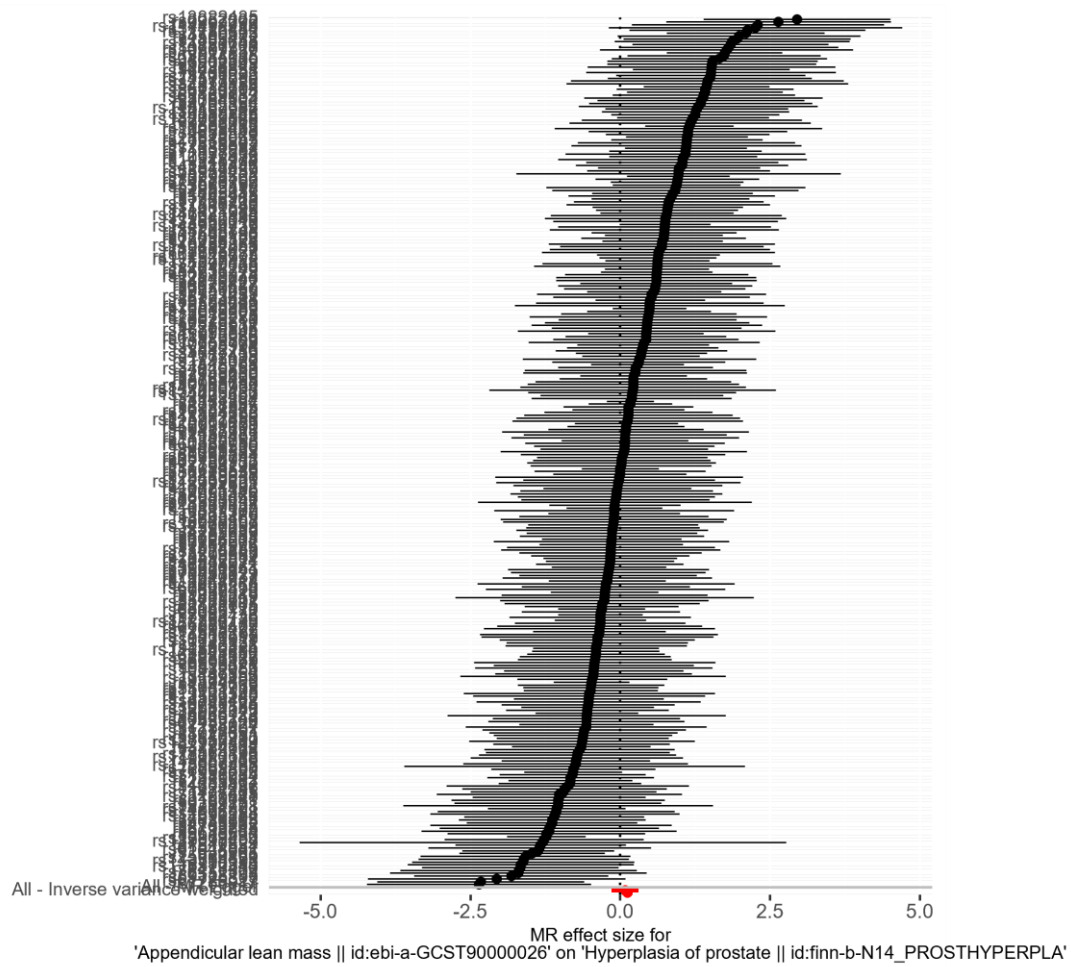

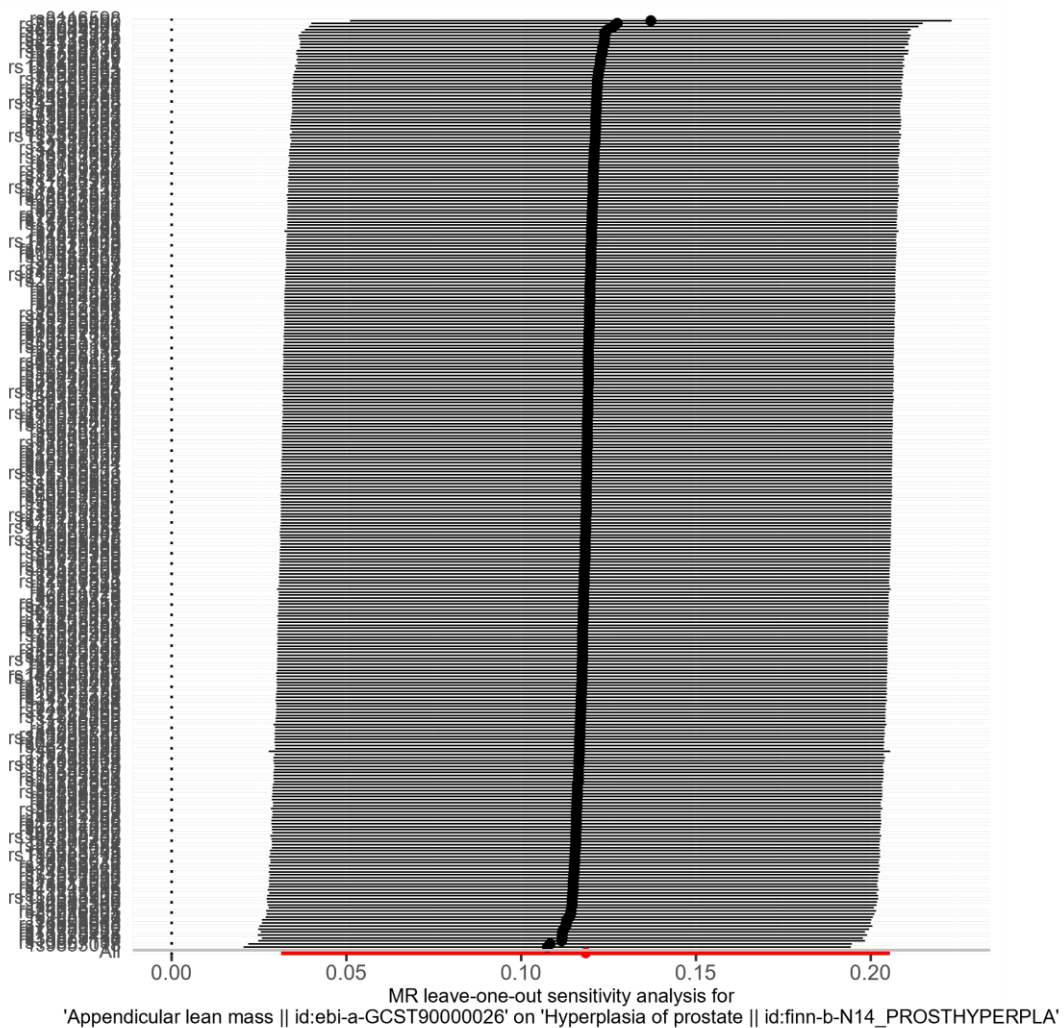

# MR Method

- Inverse variance weighted
- MR Egger

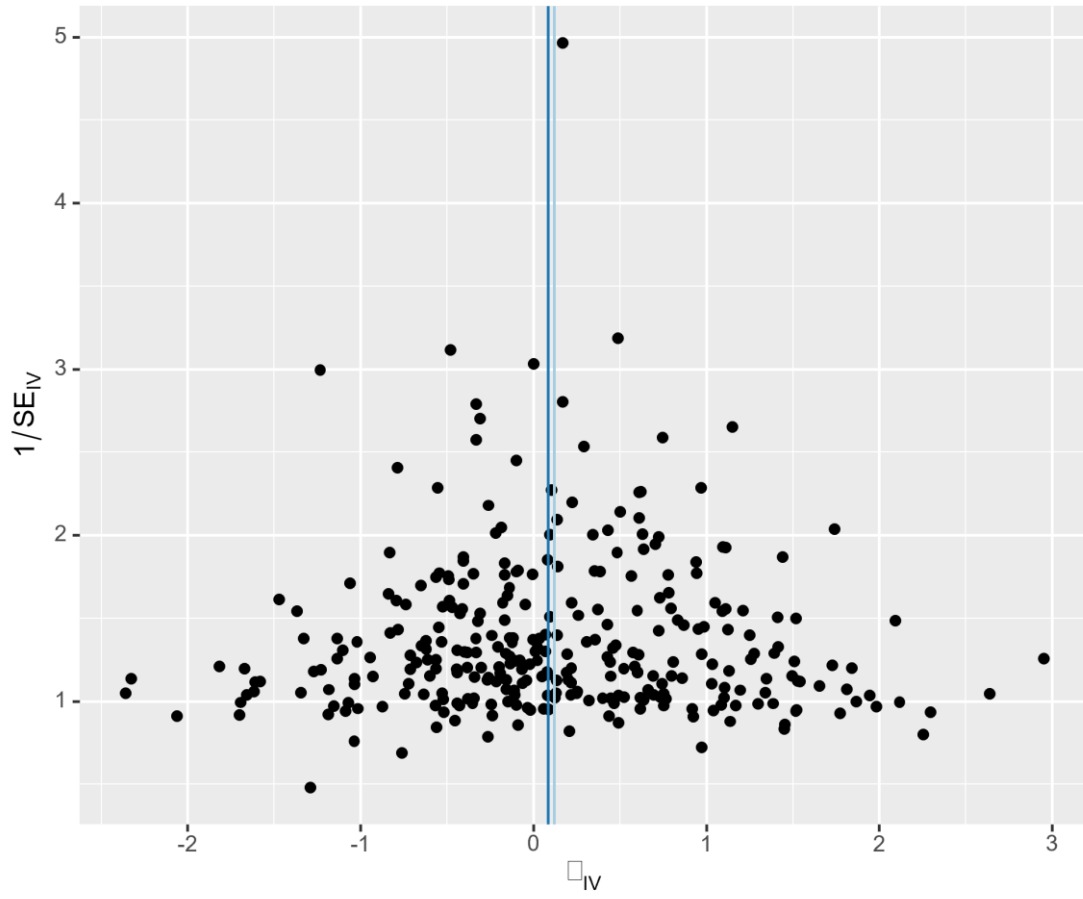

Supplement: Supplementary file 1 [file DataSheet_2.pdf]
