# Supplementary material for: The causal relationship between sarcopenic obesity factors and benign prostate hyperplasia
Source: Front Endocrinol (Lausanne). 2023 Nov 8;14:1290639. doi: 10.3389/fendo.2023.1290639 (PMC10663947; doi:10.3389/fendo.2023.1290639)
Supplement: Supplementary file 2 [file DataSheet_3.pdf]

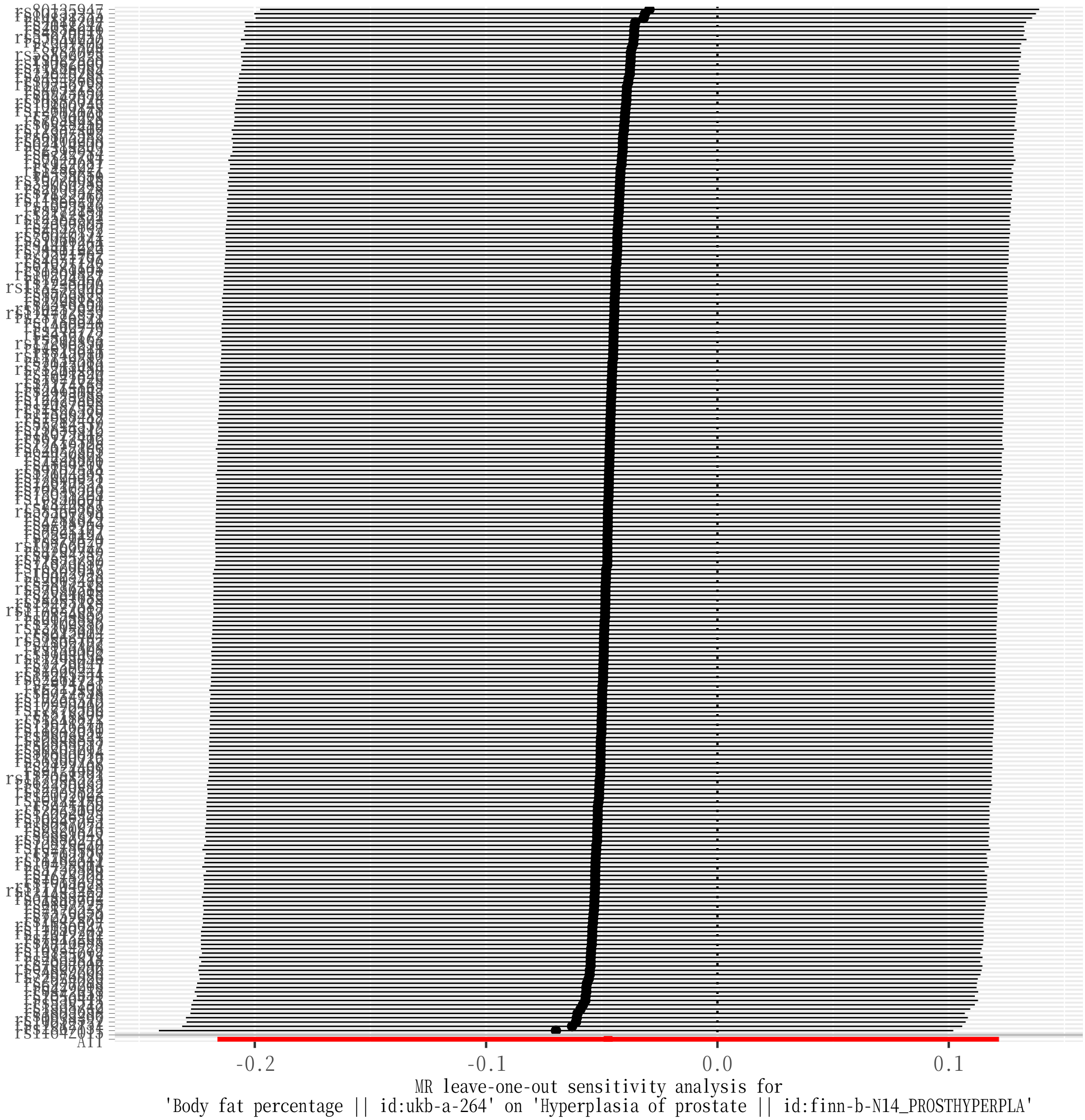
All
-0.2
-0.1
0.0
0.1
MR leave-one-out sensitivity analysis for
'Body fat percentage || id:ukb-a-264' on 'Hyperplasia of prostate || id:finn-b-N14_PROSTHYPERPLA'

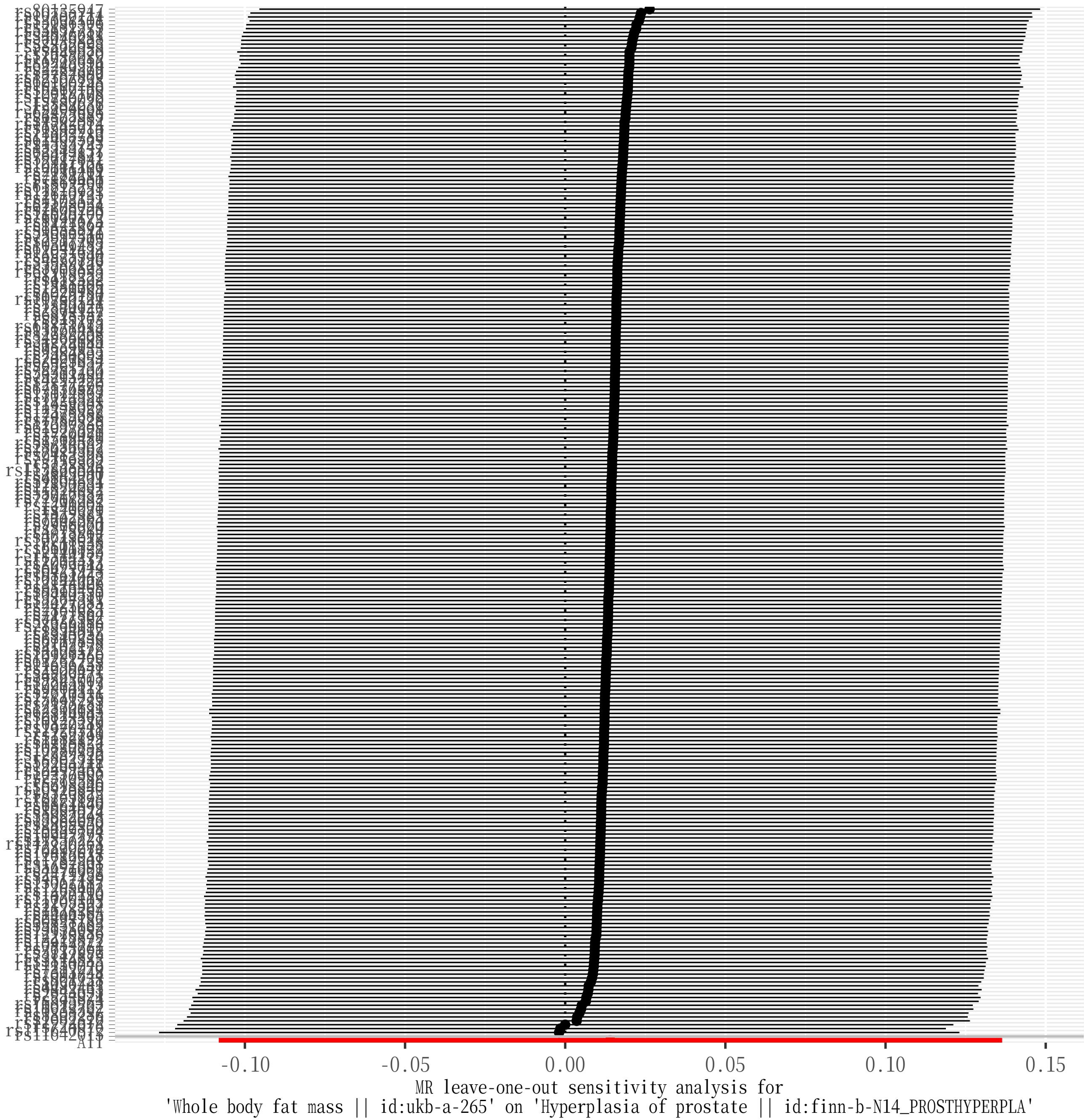
All
-0.10
-0.05
0.00
0.05
0.10
0.15
MR leave-one-out sensitivity analysis for
'Whole body fat mass || id:ukb-a-265' on 'Hyperplasia of prostate || id:finn-b-N14_PROSTHYPERPLA'

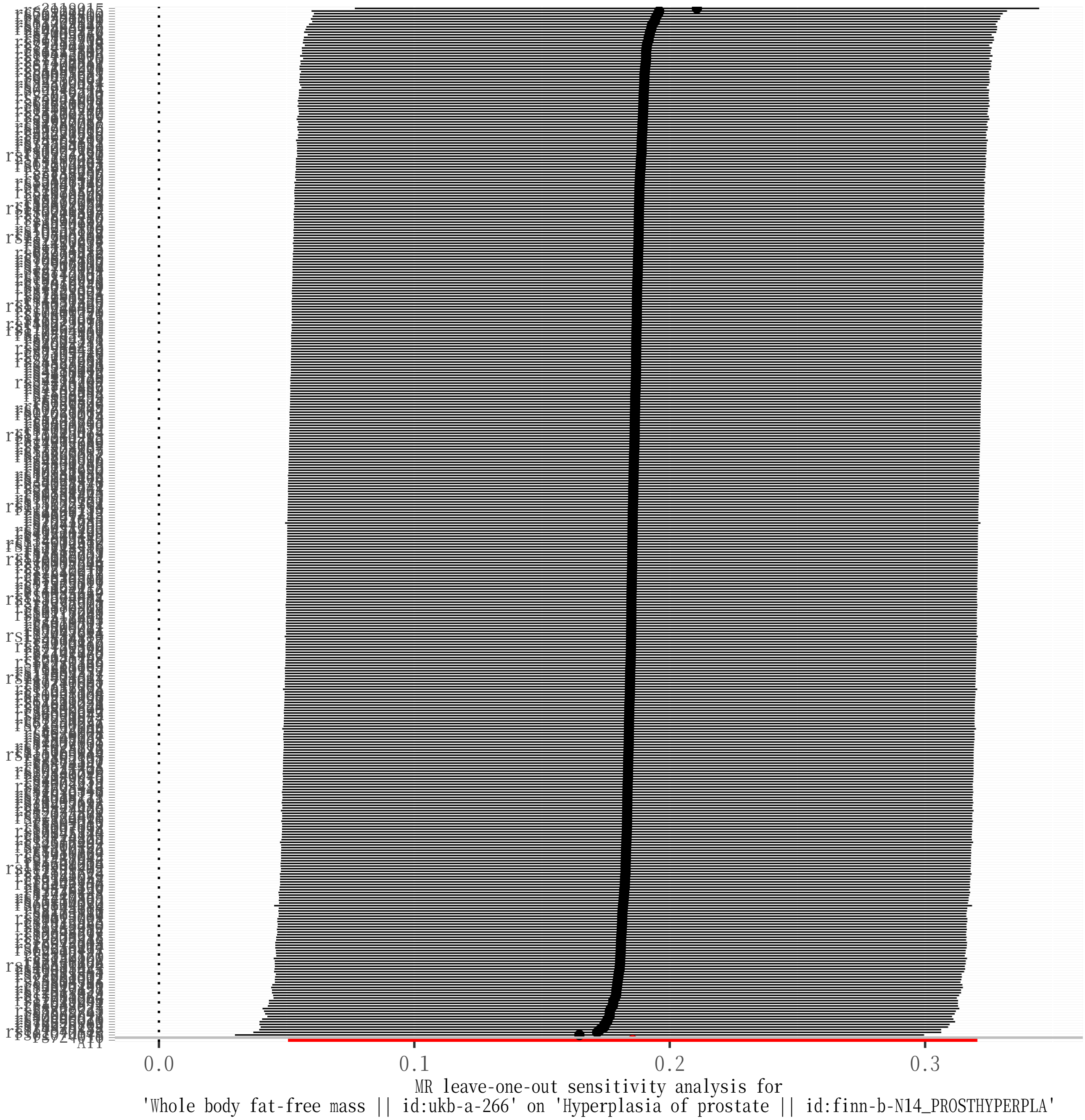
All
0.0
0.1
0.2
0.3
MR leave-one-out sensitivity analysis for
'Whole body fat-free mass || id:ukb-a-266' on 'Hyperplasia of prostate || id:finn-b-N14_PROSTHYPERPLA'

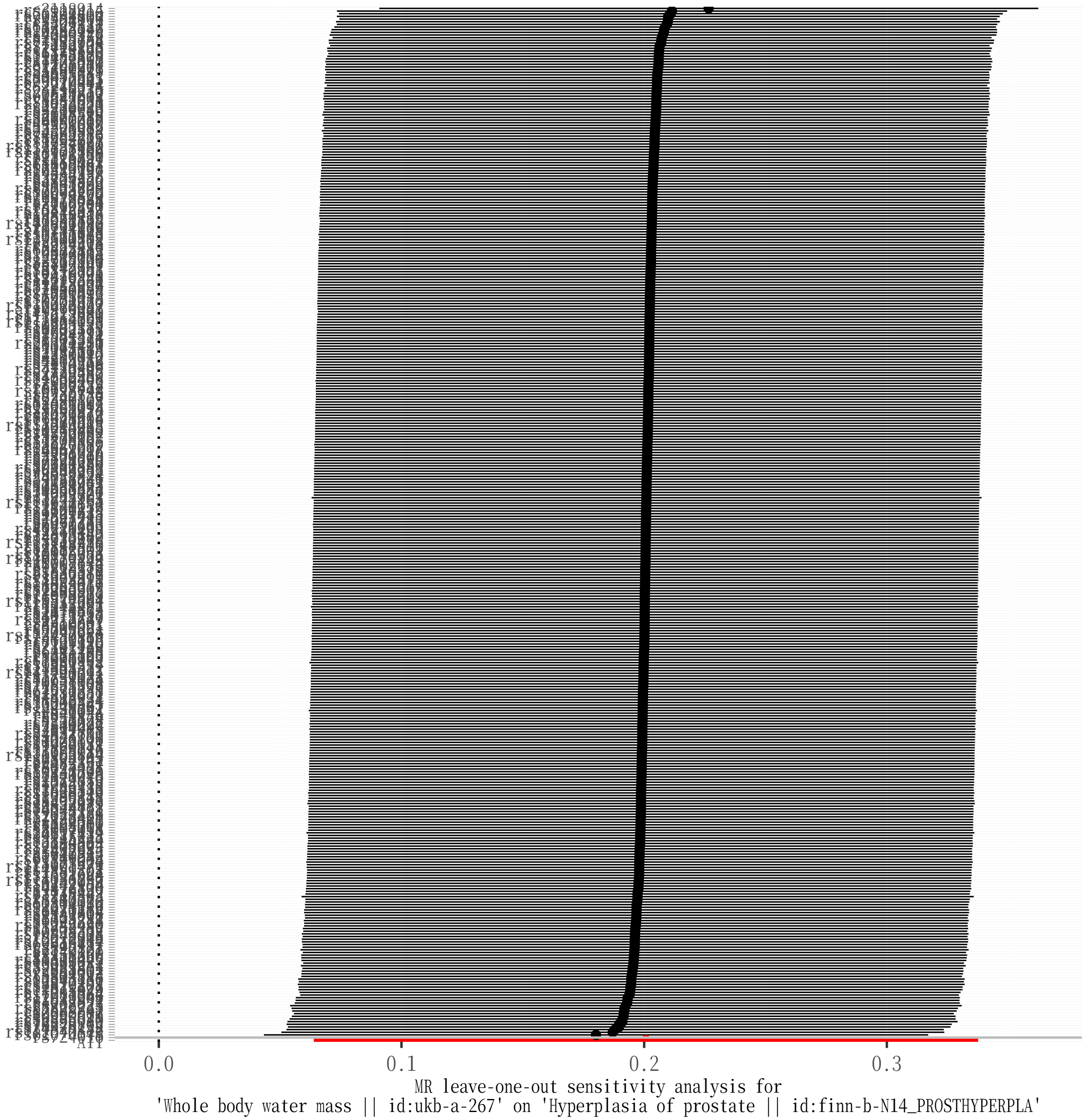
All
0.0
0.1
0.2
0.3
MR leave-one-out sensitivity analysis for
'Whole body water mass || id:ukb-a-267' on 'Hyperplasia of prostate || id:finn-b-N14_PROSTHYPERPLA'

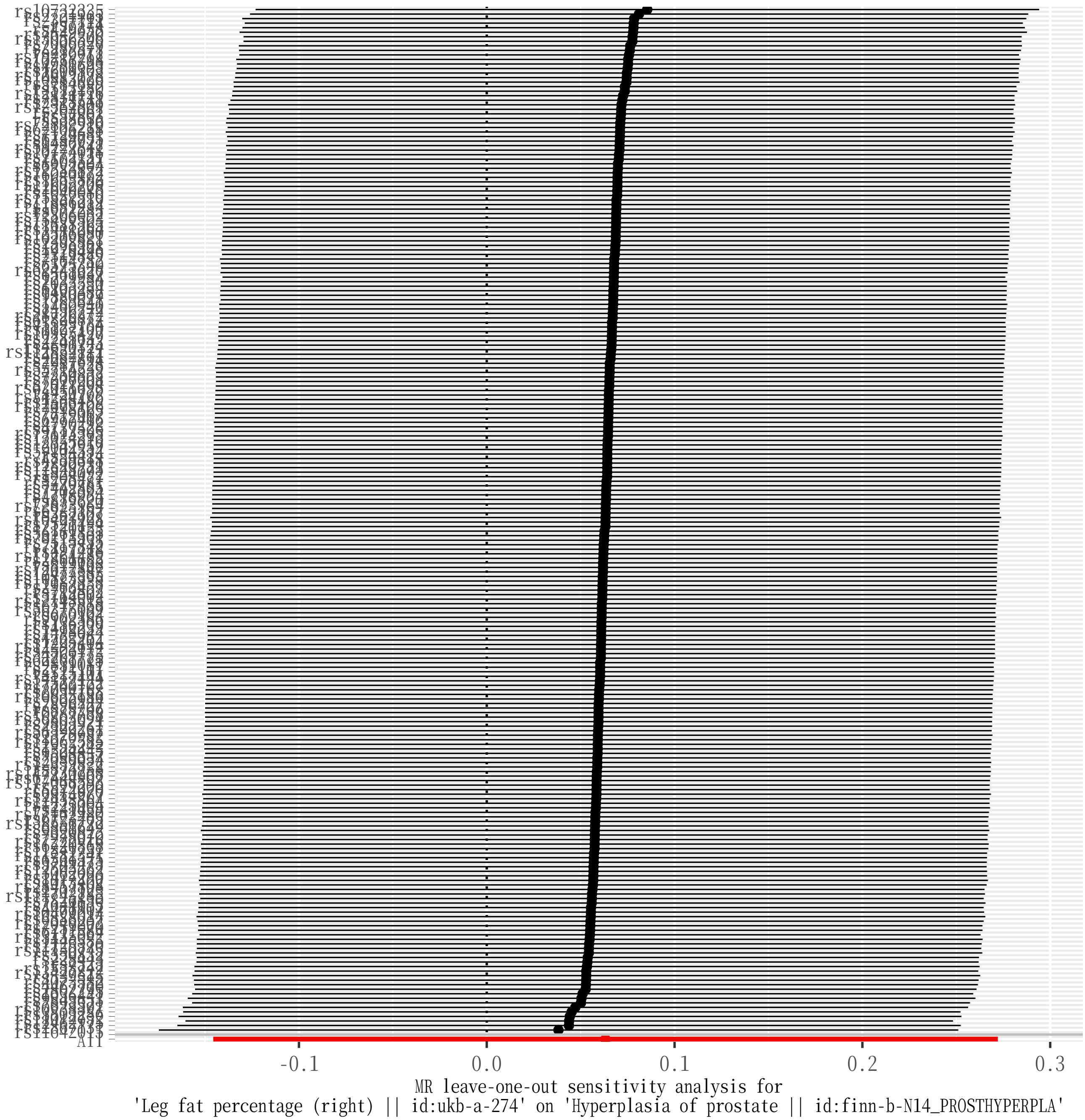
All
-0.1
0.0
0.1
0.2
0.3
MR leave-one-out sensitivity analysis for
'Leg fat percentage (right) || id:ukb-a-274' on 'Hyperplasia of prostate || id:finn-b-N14_PROSTHYPERPLA'

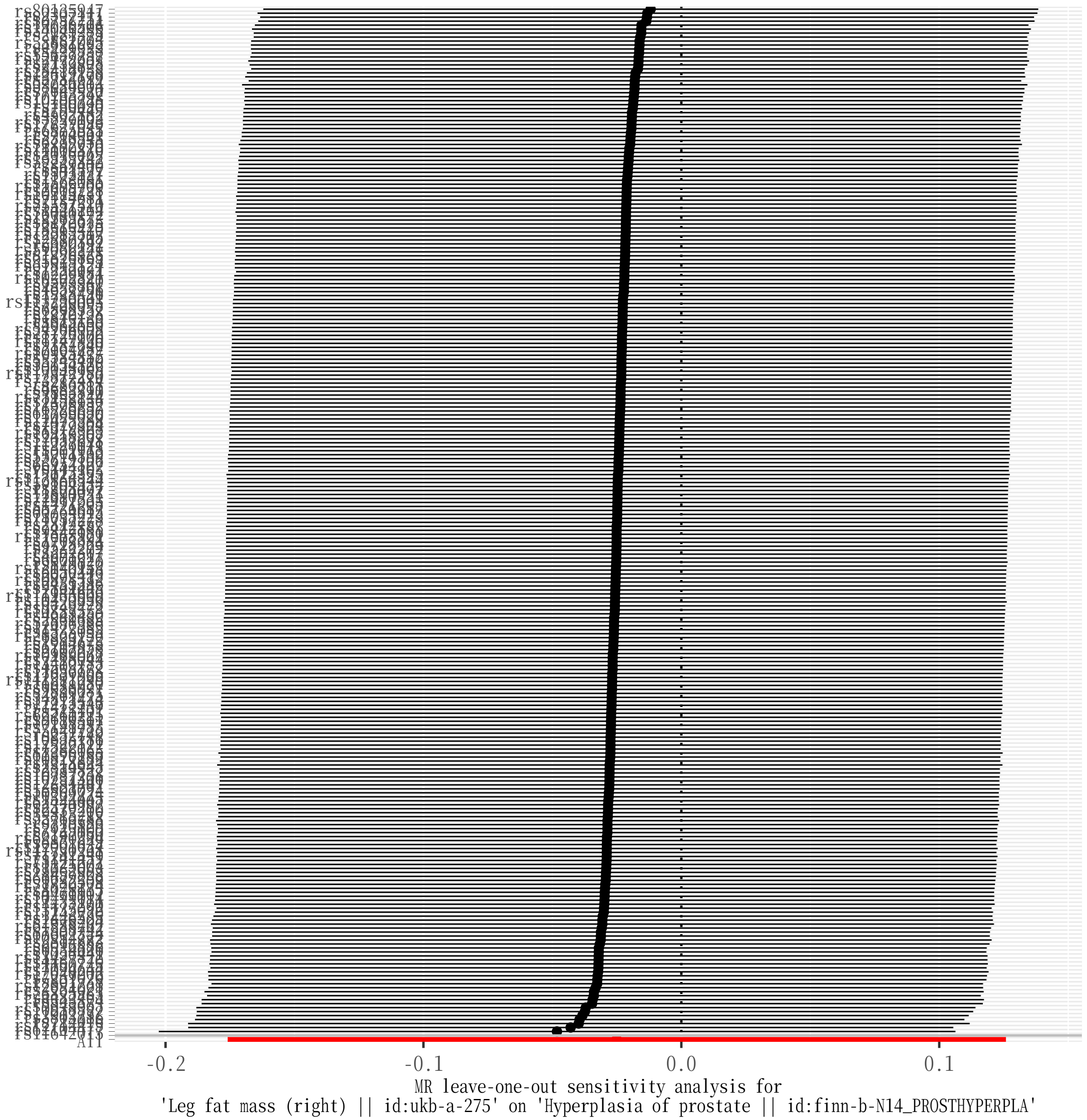
All
-0.2
-0.1
0.0
0.1
MR leave-one-out sensitivity analysis for
'Leg fat mass (right) || id:ukb-a-275' on 'Hyperplasia of prostate || id:finn-b-N14_PROSTHYPERPLA'

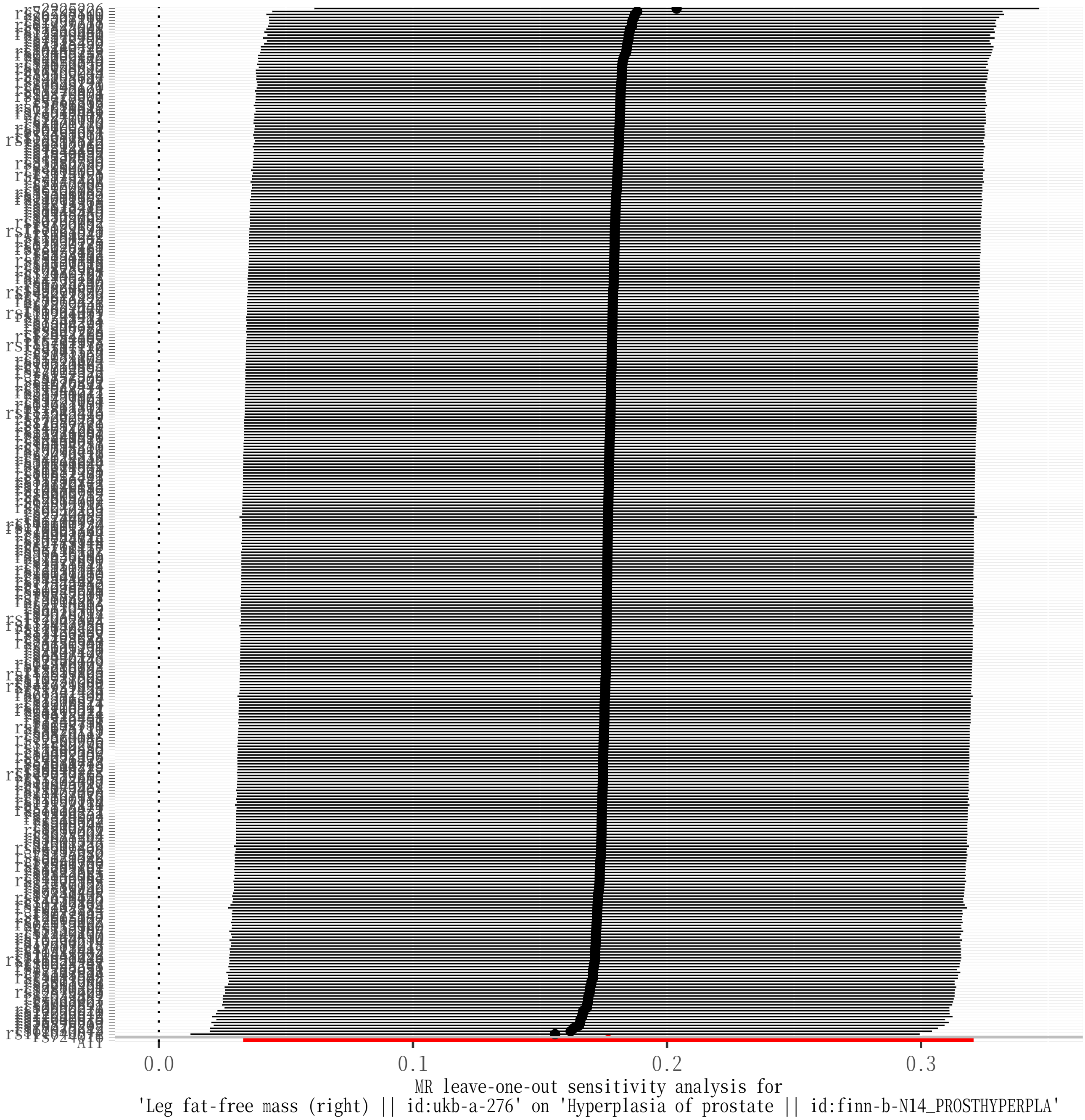
All
0.0
0.1
0.2
0.3
MR leave-one-out sensitivity analysis for
'Leg fat-free mass (right) || id:ukb-a-276' on 'Hyperplasia of prostate || id:finn-b-N14_PROSTHYPERPLA'

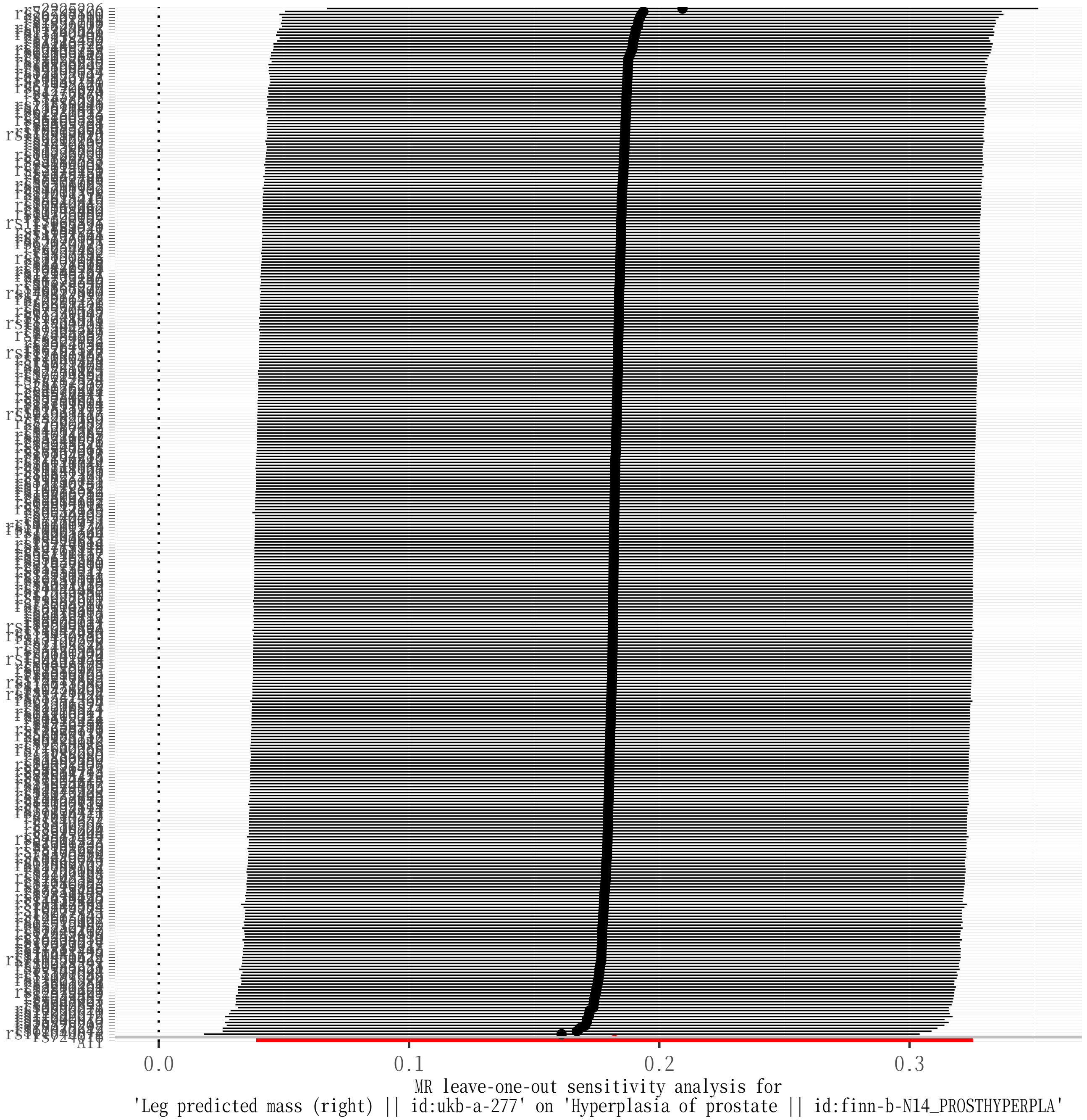
All
0.0
0.1
0.2
0.3
MR leave-one-out sensitivity analysis for
'Leg predicted mass (right) || id:ukb-a-277' on 'Hyperplasia of prostate || id:finn-b-N14_PROSTHYPERPLA'

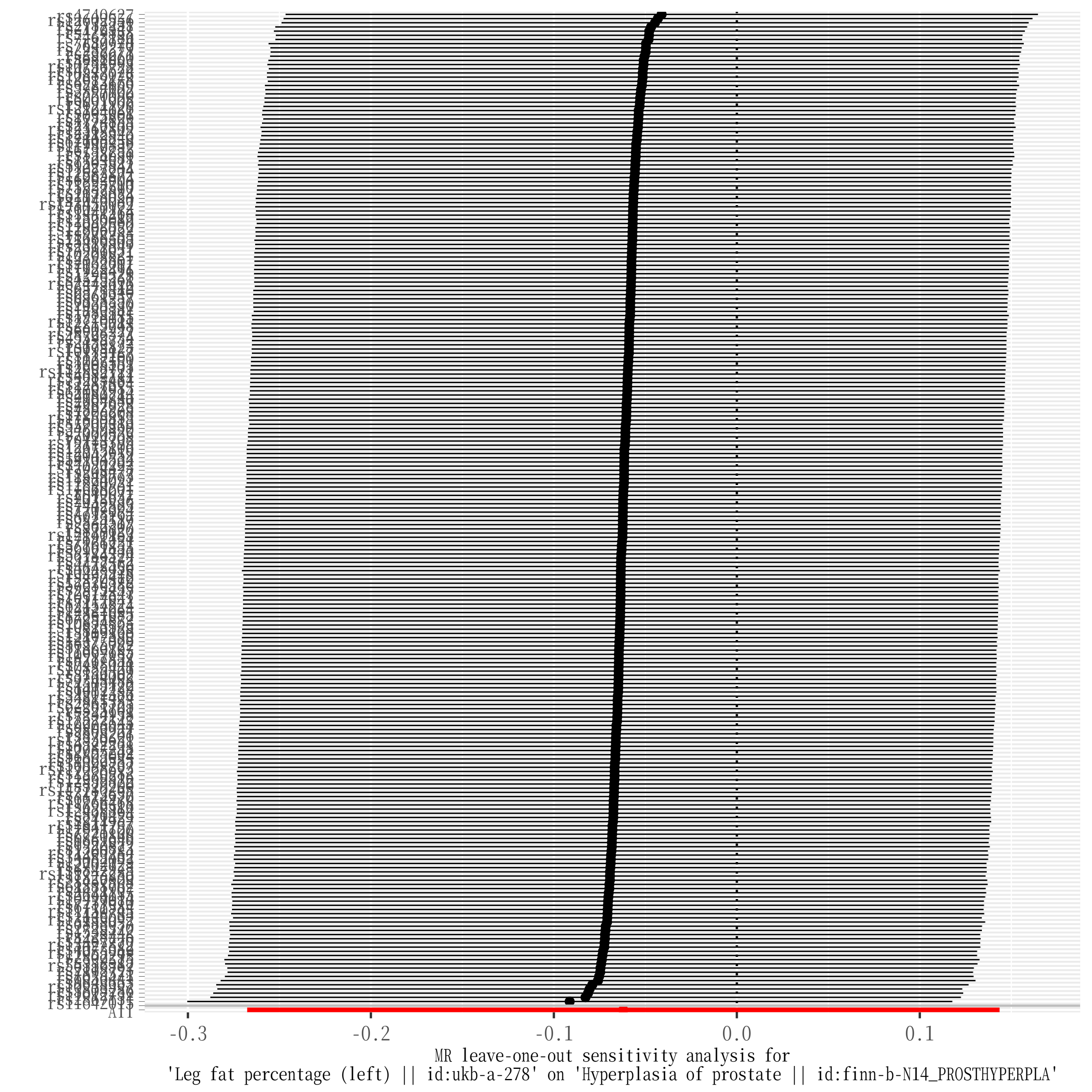
All
-0.3
-0.2
-0.1
0.0
0.1
MR leave-one-out sensitivity analysis for
'Leg fat percentage (left) || id:ukb-a-278' on 'Hyperplasia of prostate || id:finn-b-N14_PROSTHYPERPLA'

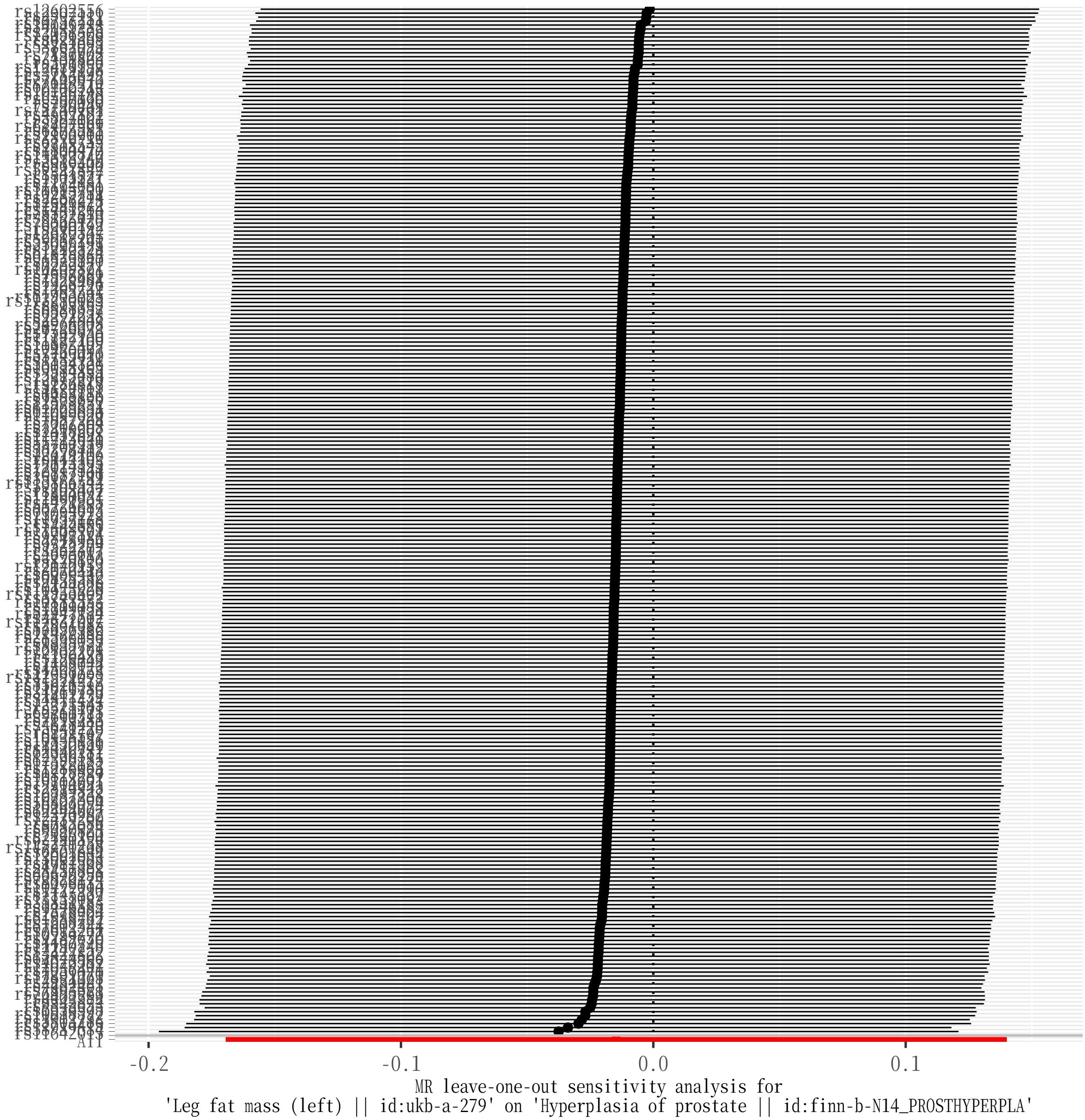
All
-0.2
-0.1
0.0
0.1
MR leave-one-out sensitivity analysis for
'Leg fat mass (left) || id:ukb-a-279' on 'Hyperplasia of prostate || id:finn-b-N14_PROSTHYPERPLA'

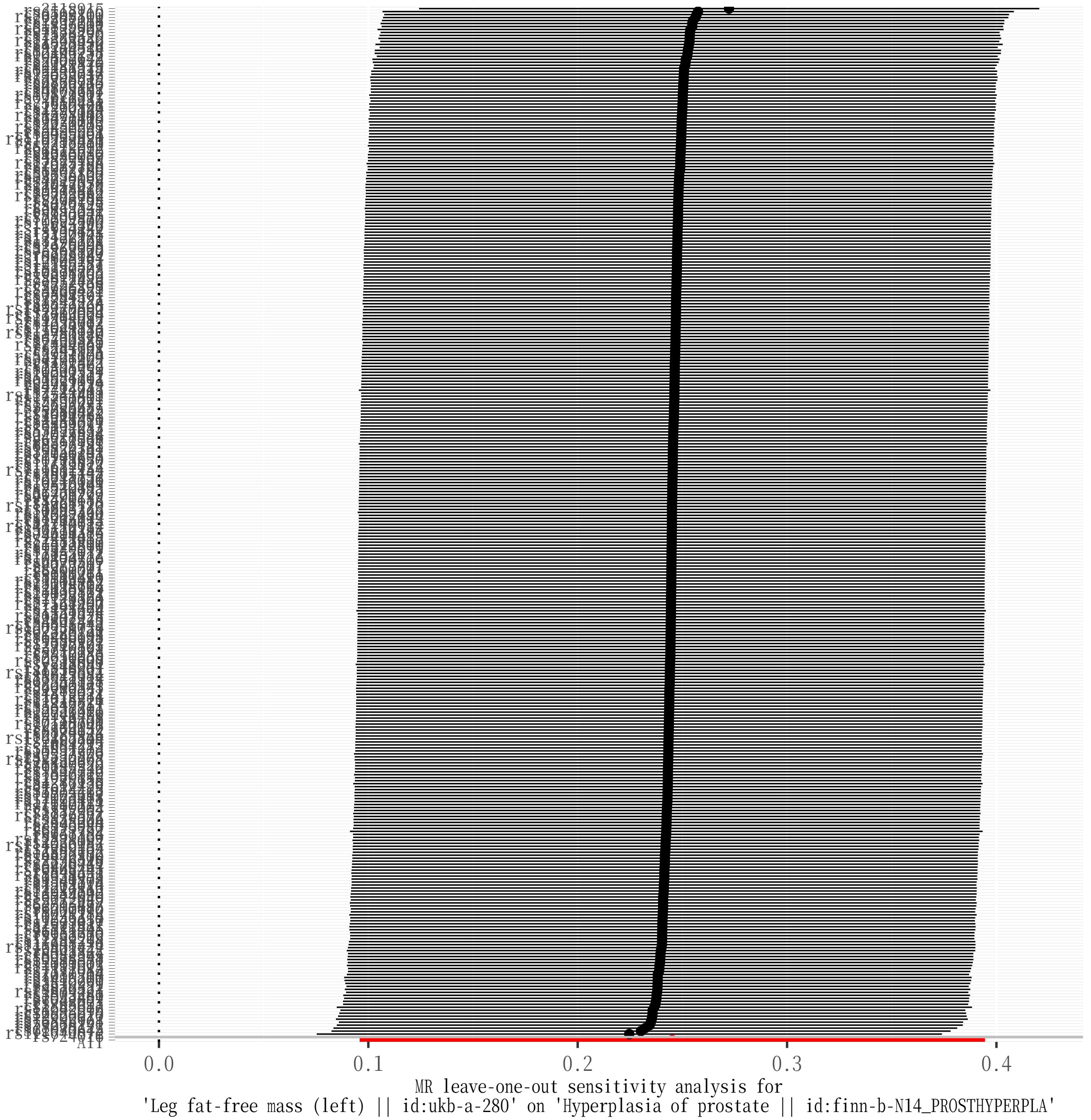
All
0.0
0.1
0.2
0.3
0.4
MR leave-one-out sensitivity analysis for
'Leg fat-free mass (left) || id:ukb-a-280' on 'Hyperplasia of prostate || id:finn-b-N14_PROSTHYPERPLA'

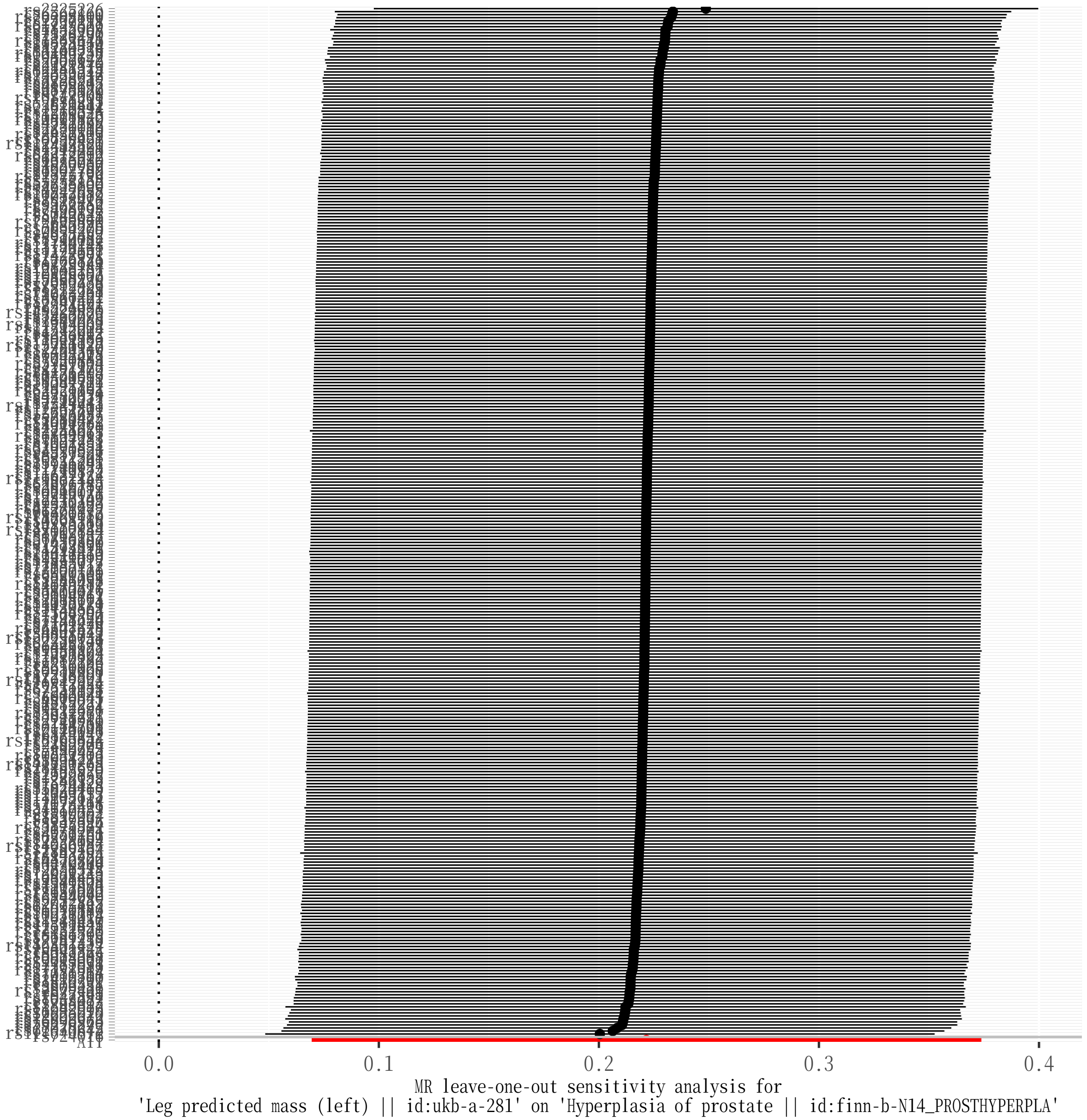
All
0.0
0.1
0.2
0.3
0.4
MR leave-one-out sensitivity analysis for
'Leg predicted mass (left) || id:ukb-a-281' on 'Hyperplasia of prostate || id:finn-b-N14_PROSTHYPERPLA'

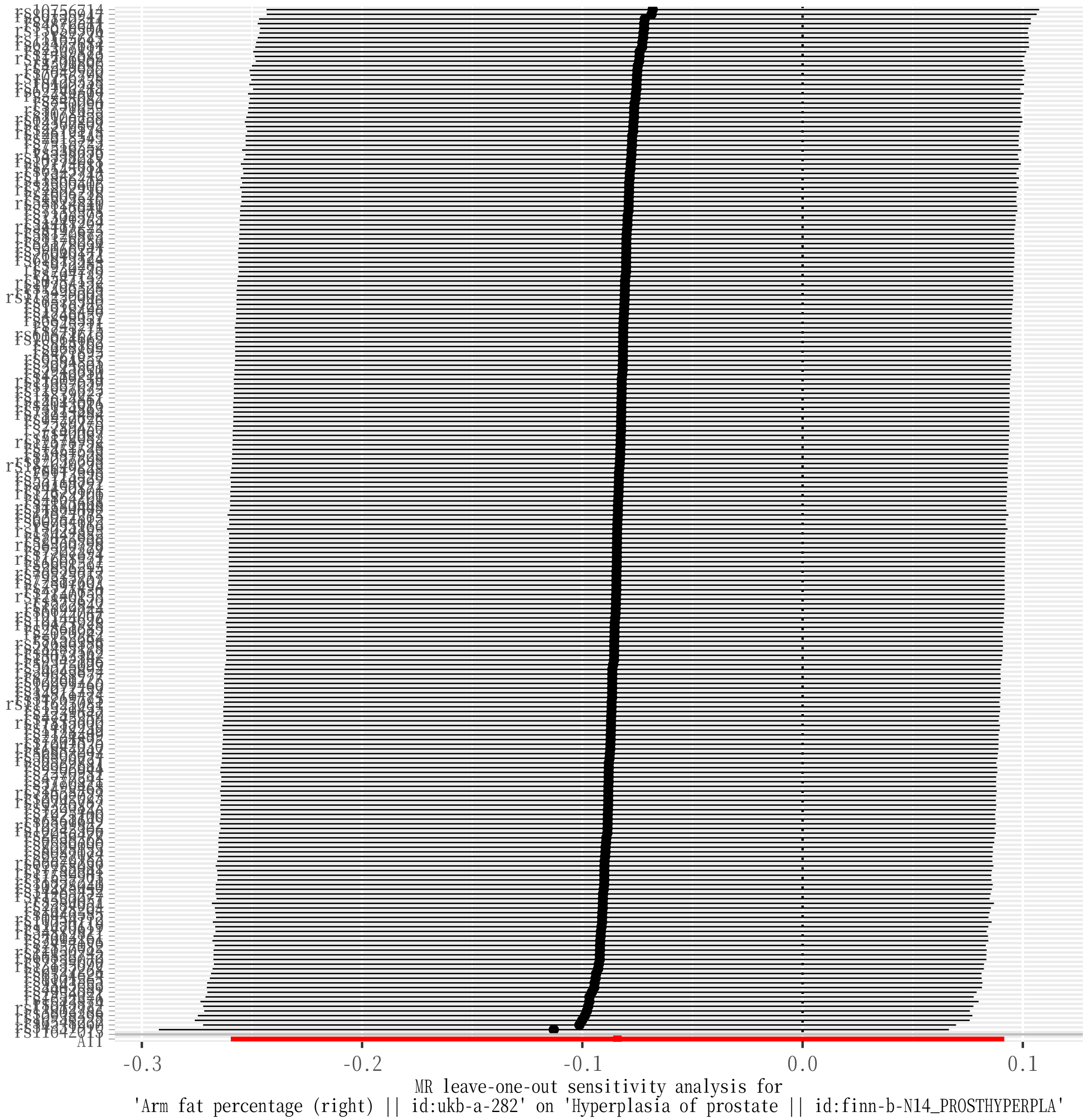
All
-0.3
-0.2
-0.1
0.0
0.1
MR leave-one-out sensitivity analysis for
'Arm fat percentage (right) || id:ukb-a-282' on 'Hyperplasia of prostate || id:finn-b-N14_PROSTHYPERPLA'

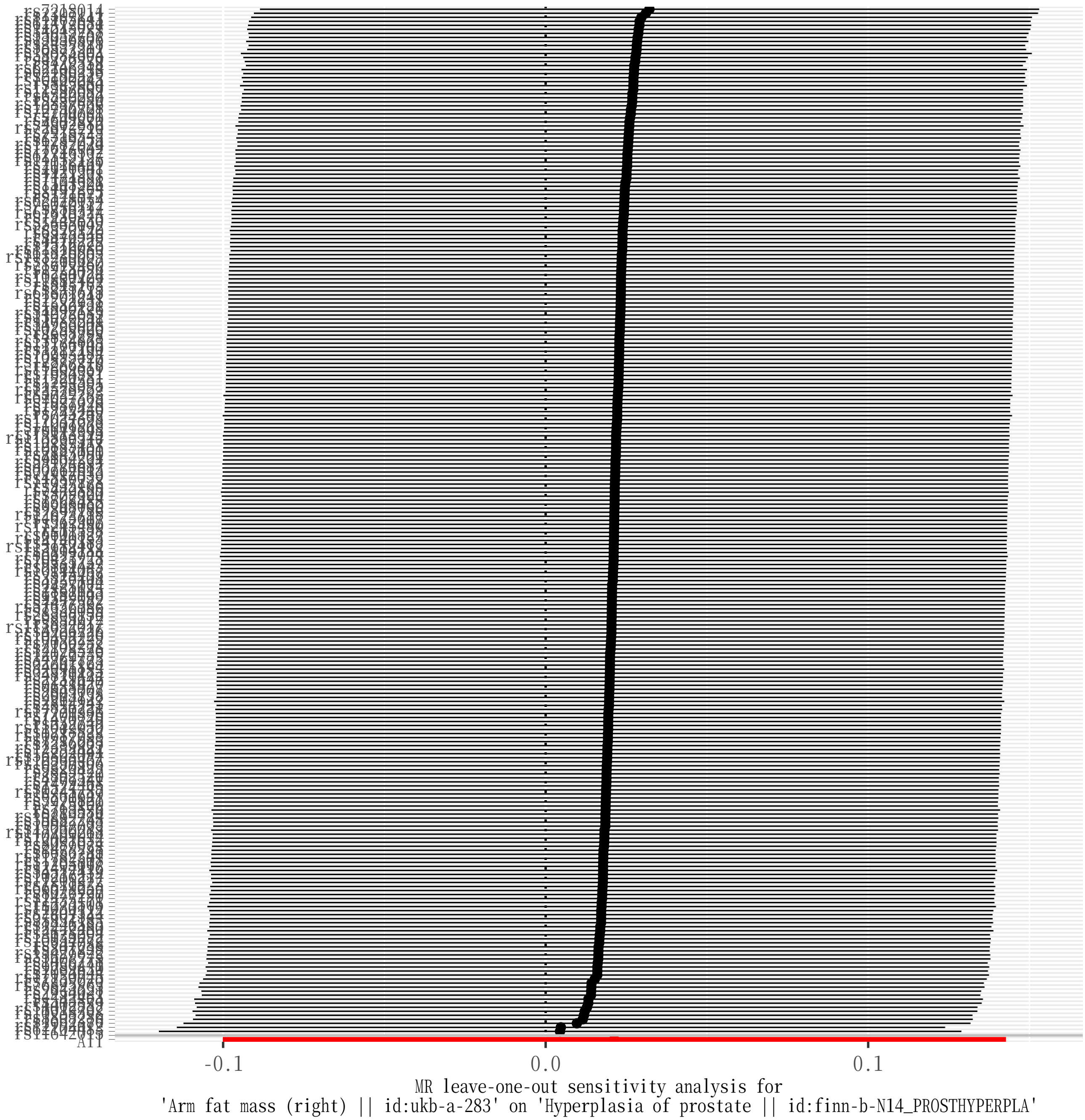
All
-0.1
0.0
0.1
MR leave-one-out sensitivity analysis for
'Arm fat mass (right) || id:ukb-a-283' on 'Hyperplasia of prostate || id:finn-b-N14_PROSTHYPERPLA'

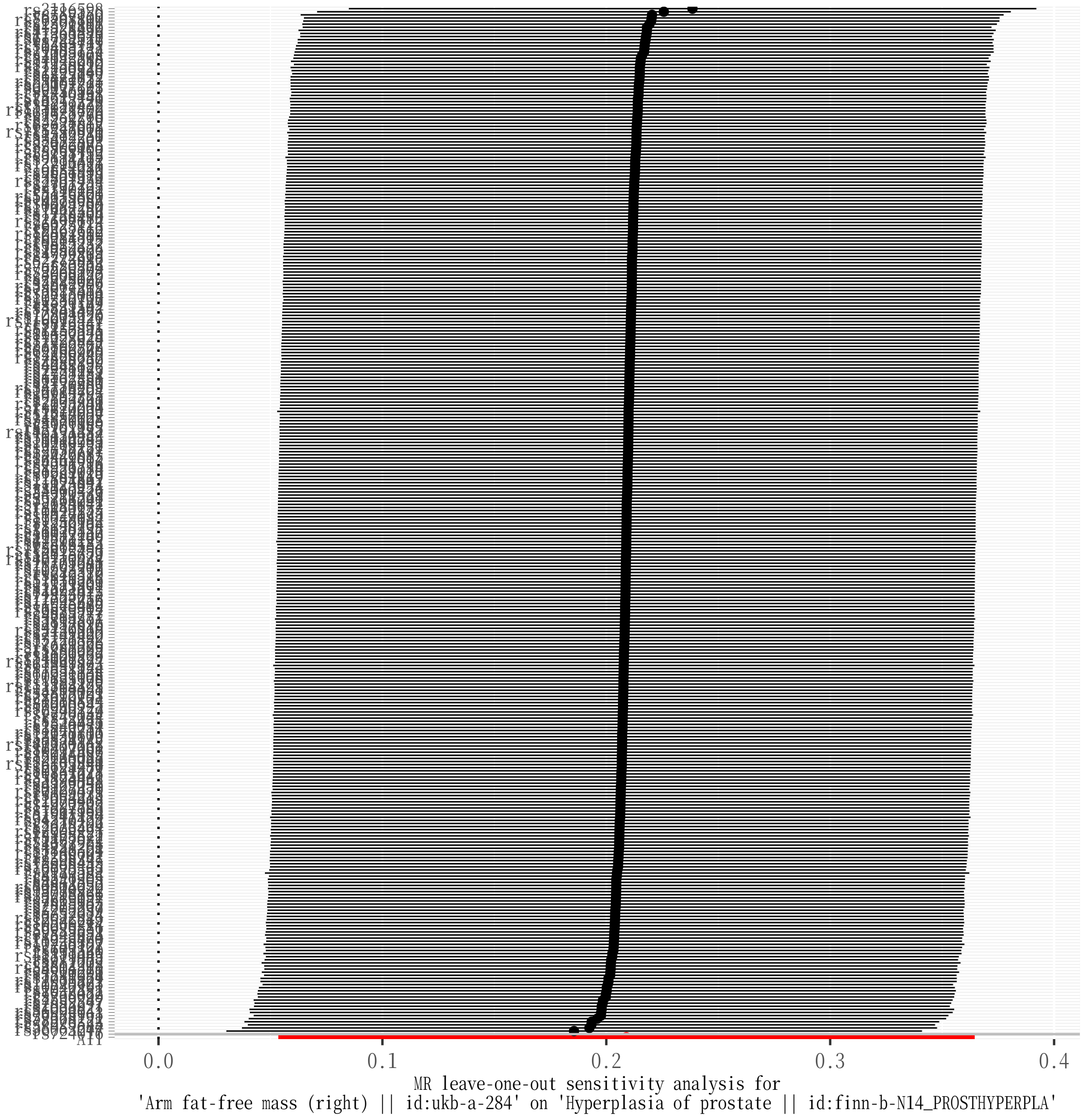
rs2116598
All
0.0
0.1
0.2
0.3
0.4
MR leave-one-out sensitivity analysis for
'Arm fat-free mass (right) || id:ukb-a-284' on 'Hyperplasia of prostate || id:finn-b-N14_PROSTHYPERPLA'

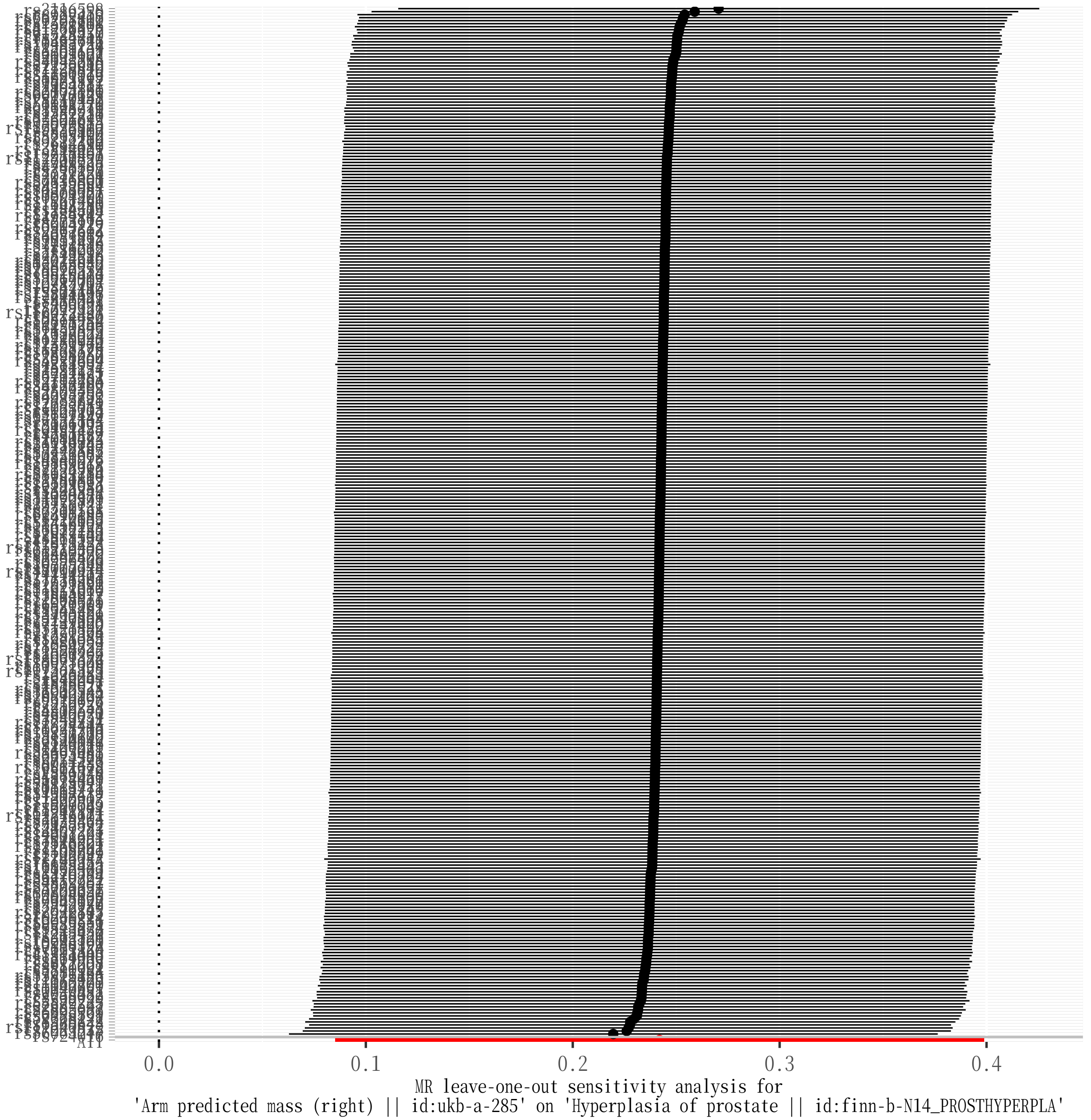
All
0.0
0.1
0.2
0.3
0.4
MR leave-one-out sensitivity analysis for
'Arm predicted mass (right) || id:ukb-a-285' on 'Hyperplasia of prostate || id:finn-b-N14_PROSTHYPERPLA'

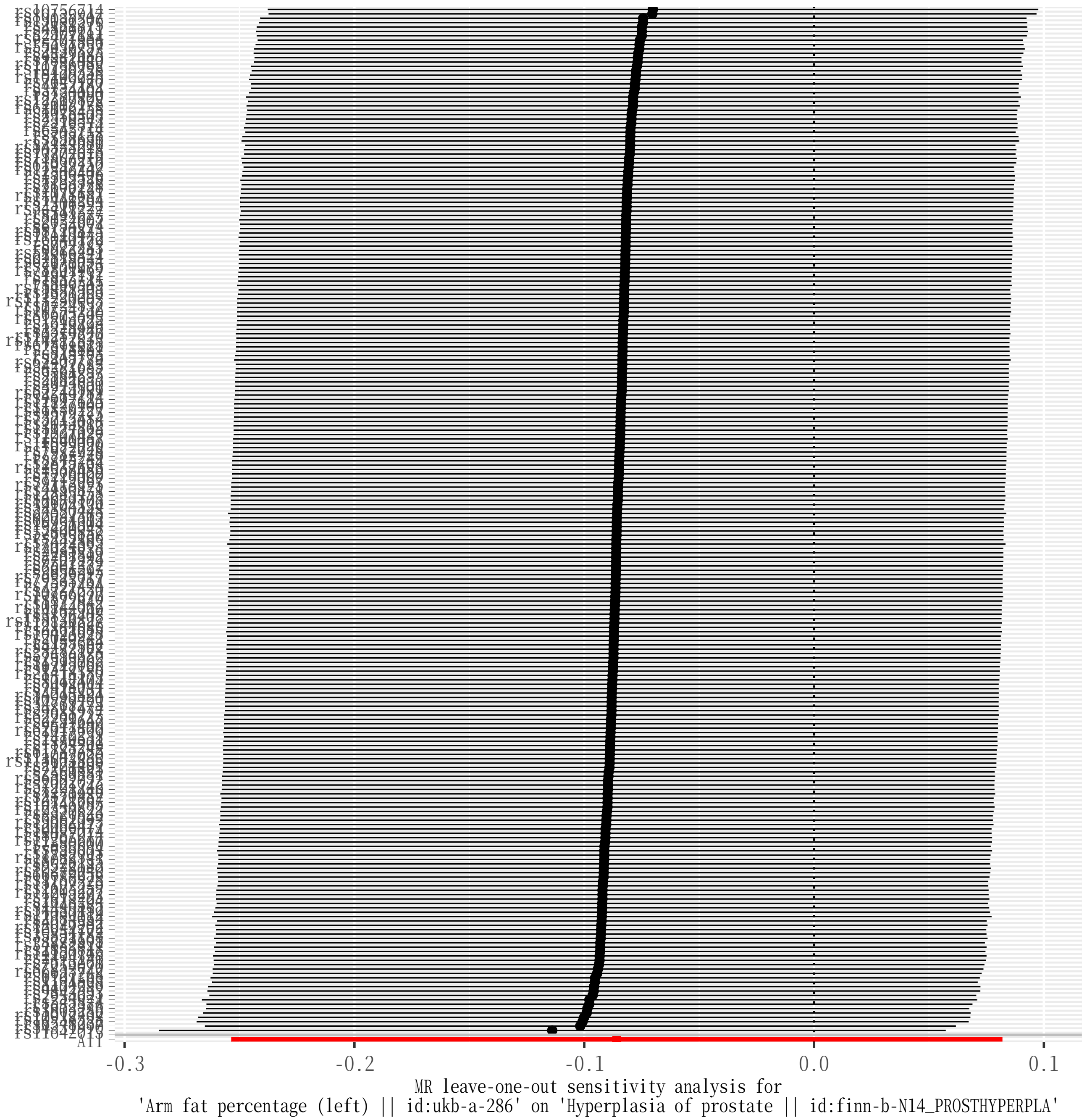
All
-0.3
-0.2
-0.1
0.0
0.1
MR leave-one-out sensitivity analysis for
'Arm fat percentage (left) || id:ukb-a-286' on 'Hyperplasia of prostate || id:finn-b-N14_PROSTHYPERPLA'

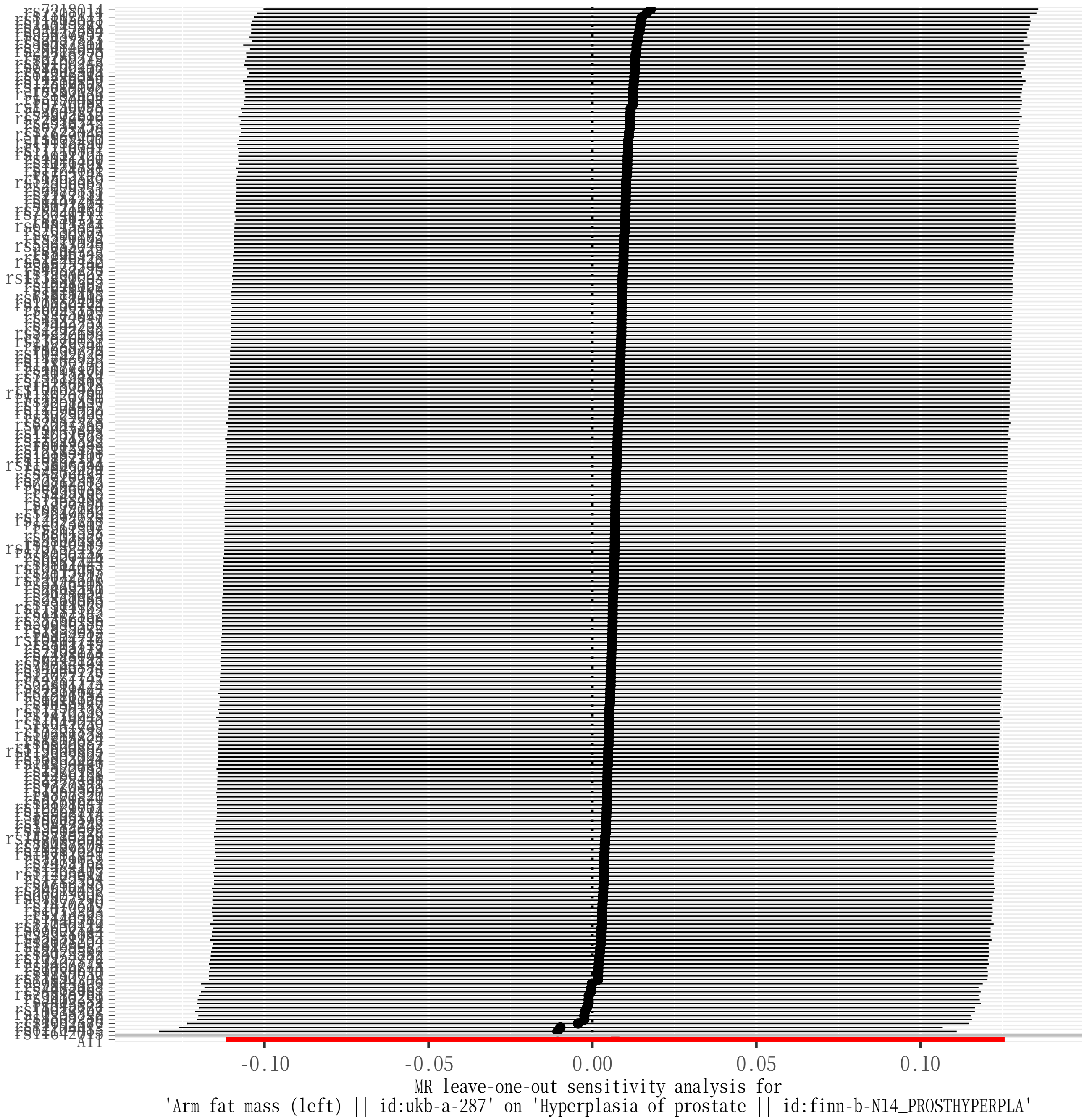
All
-0.10
-0.05
0.00
0.05
0.10
MR leave-one-out sensitivity analysis for
'Arm fat mass (left) || id:ukb-a-287' on 'Hyperplasia of prostate || id:finn-b-N14_PROSTHYPERPLA'

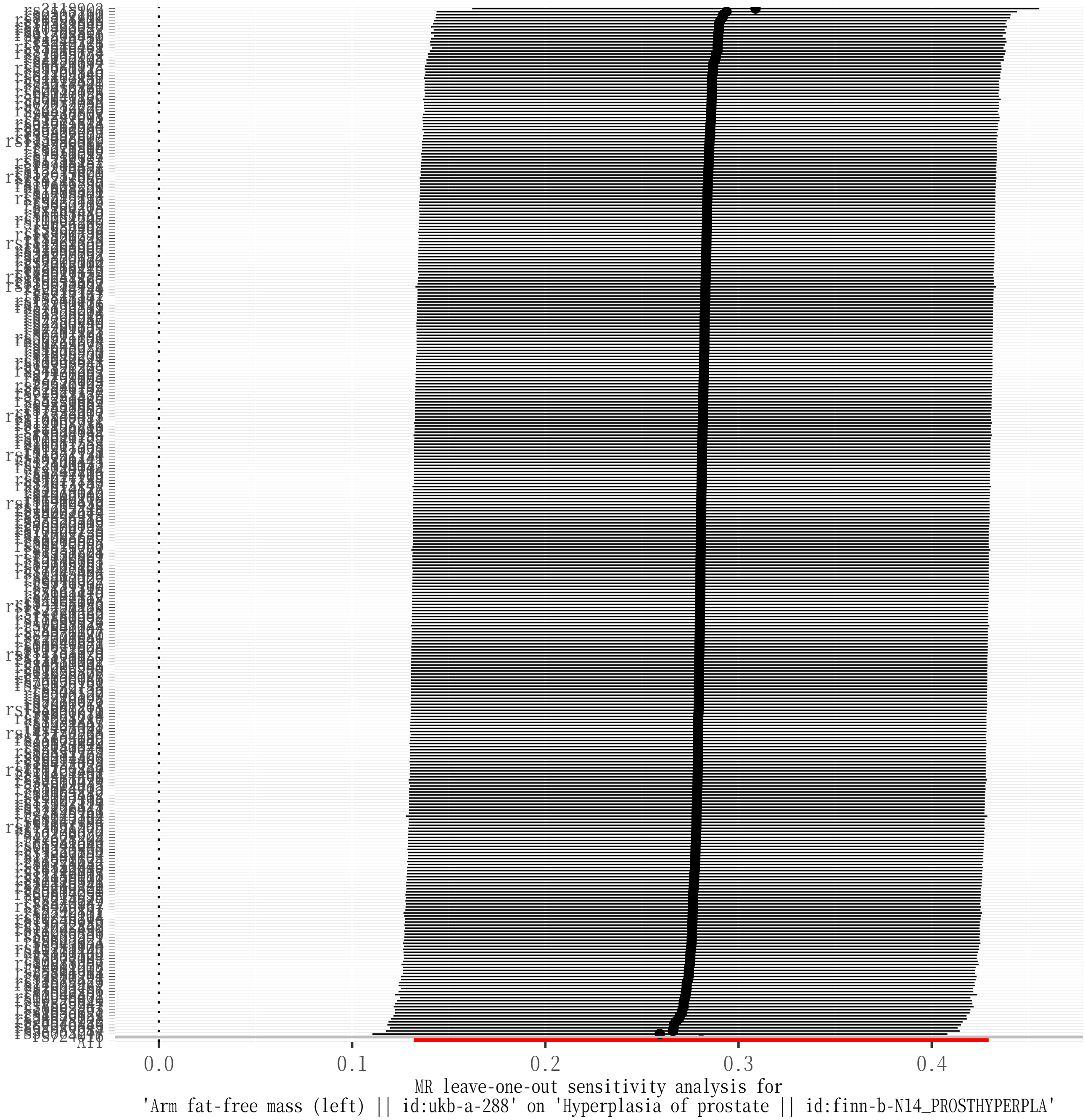

All
0.0
0.1
0.2
0.3
0.4
MR leave-one-out sensitivity analysis for
'Arm fat-free mass (left) || id:ukb-a-288' on 'Hyperplasia of prostate || id:finn-b-N14_PROSTHYPERPLA'

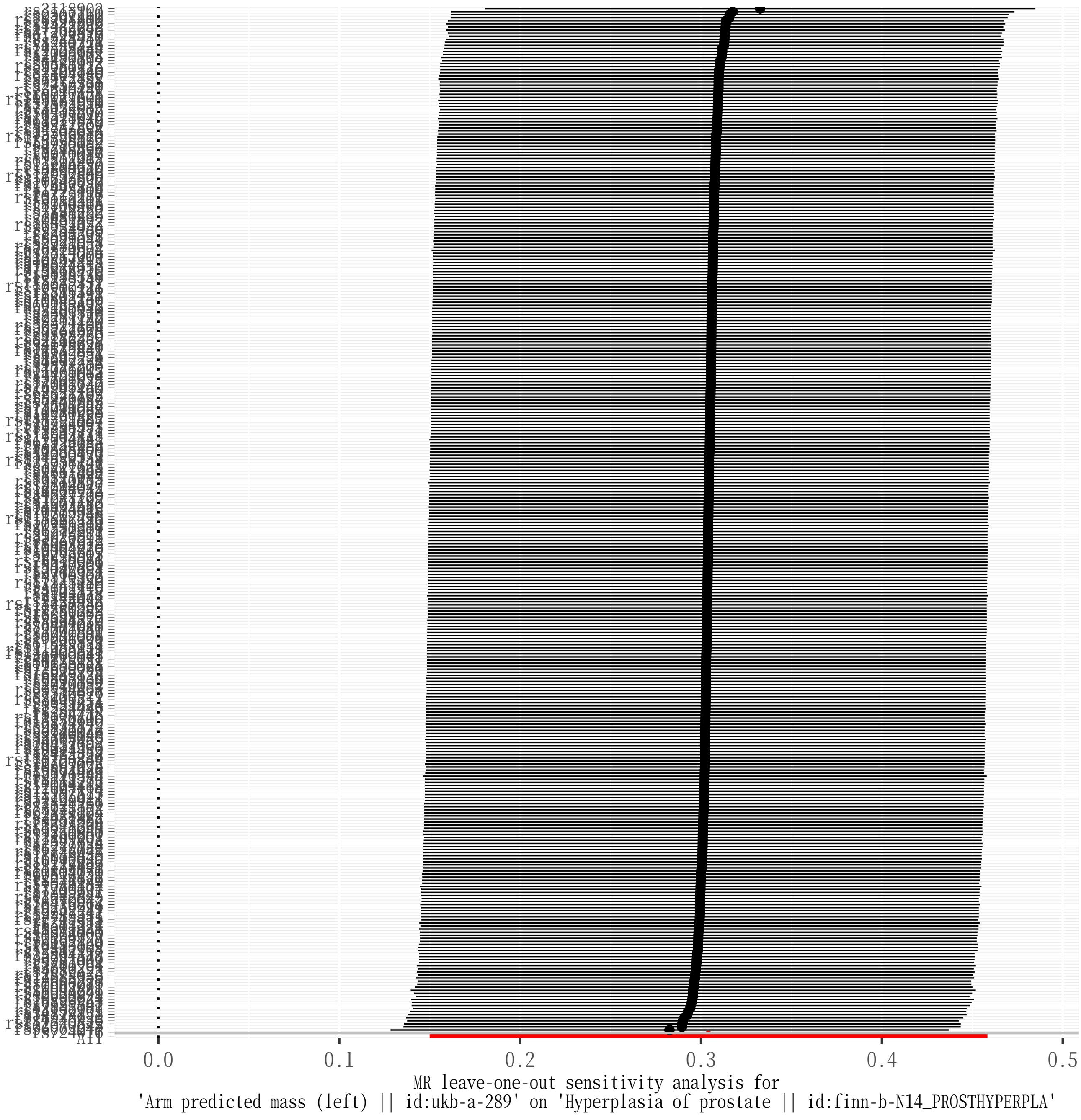
0.0
0.1
0.2
0.3
0.4
0.5
All
MR leave-one-out sensitivity analysis for
'Arm predicted mass (left) || id:ukb-a-289' on 'Hyperplasia of prostate || id:finn-b-N14_PROSTHYPERPLA'

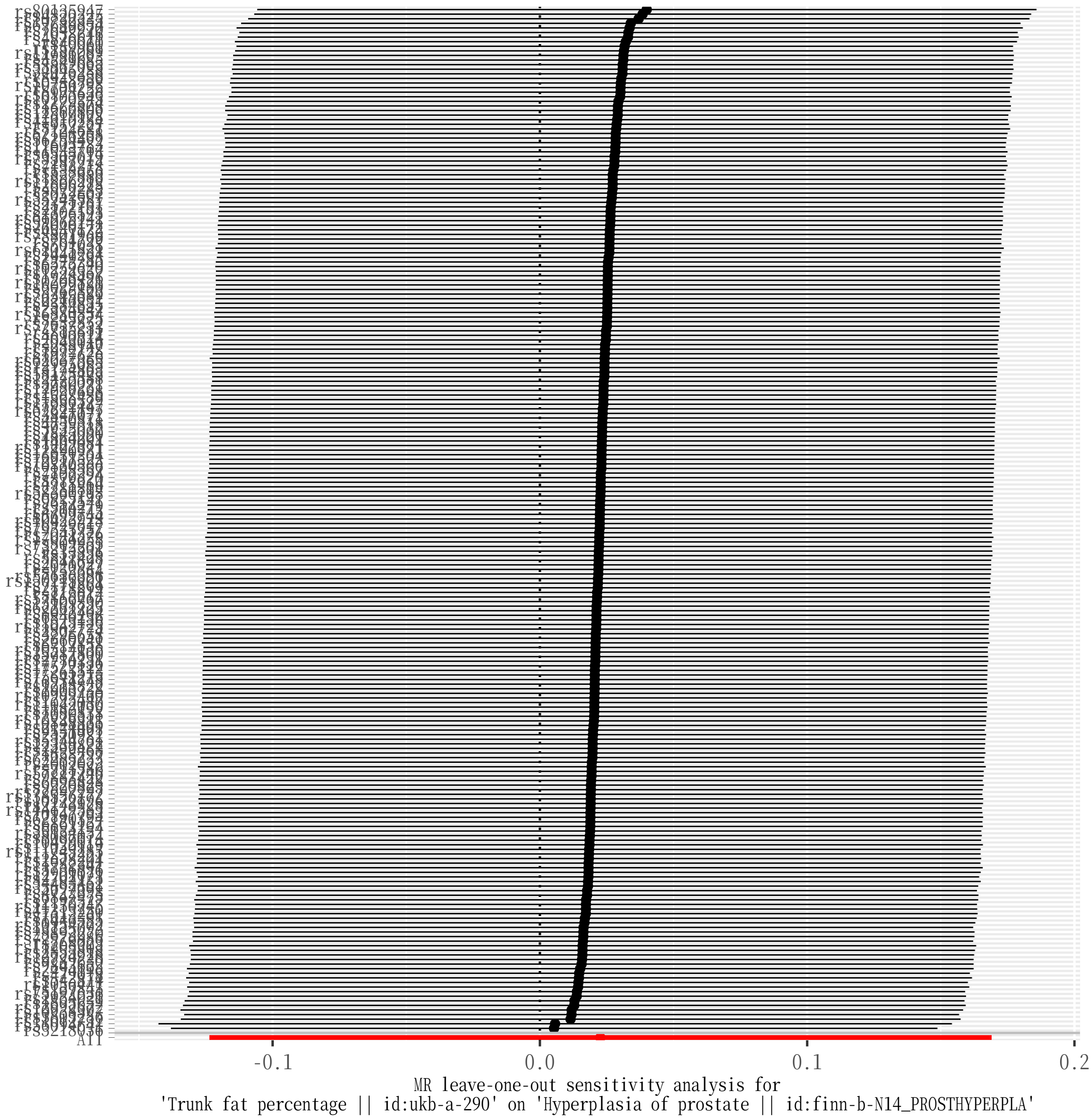

All
-0.1
0.0
0.1
0.2
MR leave-one-out sensitivity analysis for
'Trunk fat percentage || id:ukb-a-290' on 'Hyperplasia of prostate || id:finn-b-N14_PROSTHYPERPLA'

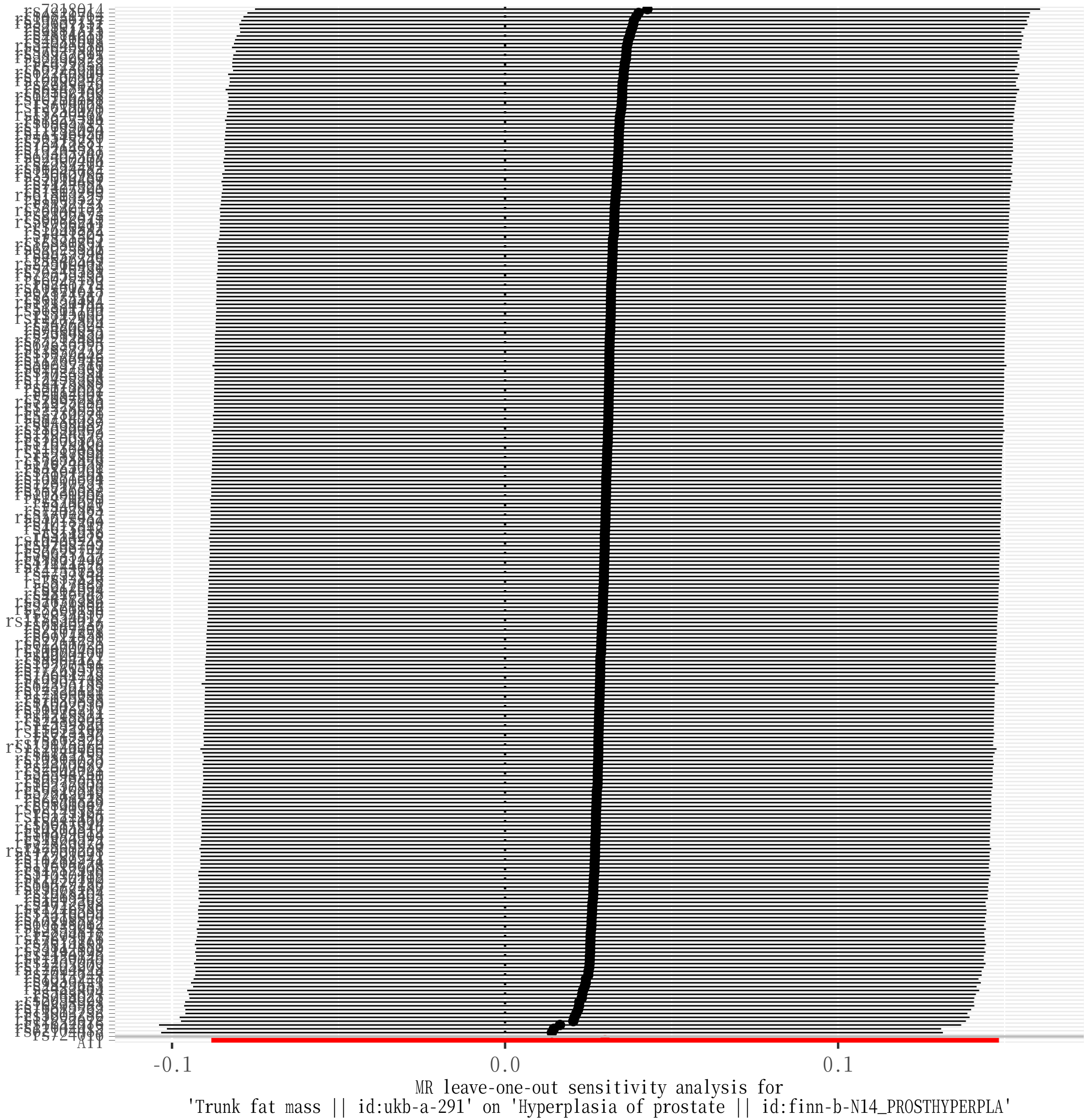
All
-0.1
0.0
0.1
MR leave-one-out sensitivity analysis for
'Trunk fat mass || id:ukb-a-291' on 'Hyperplasia of prostate || id:finn-b-N14_PROSTHYPERPLA'

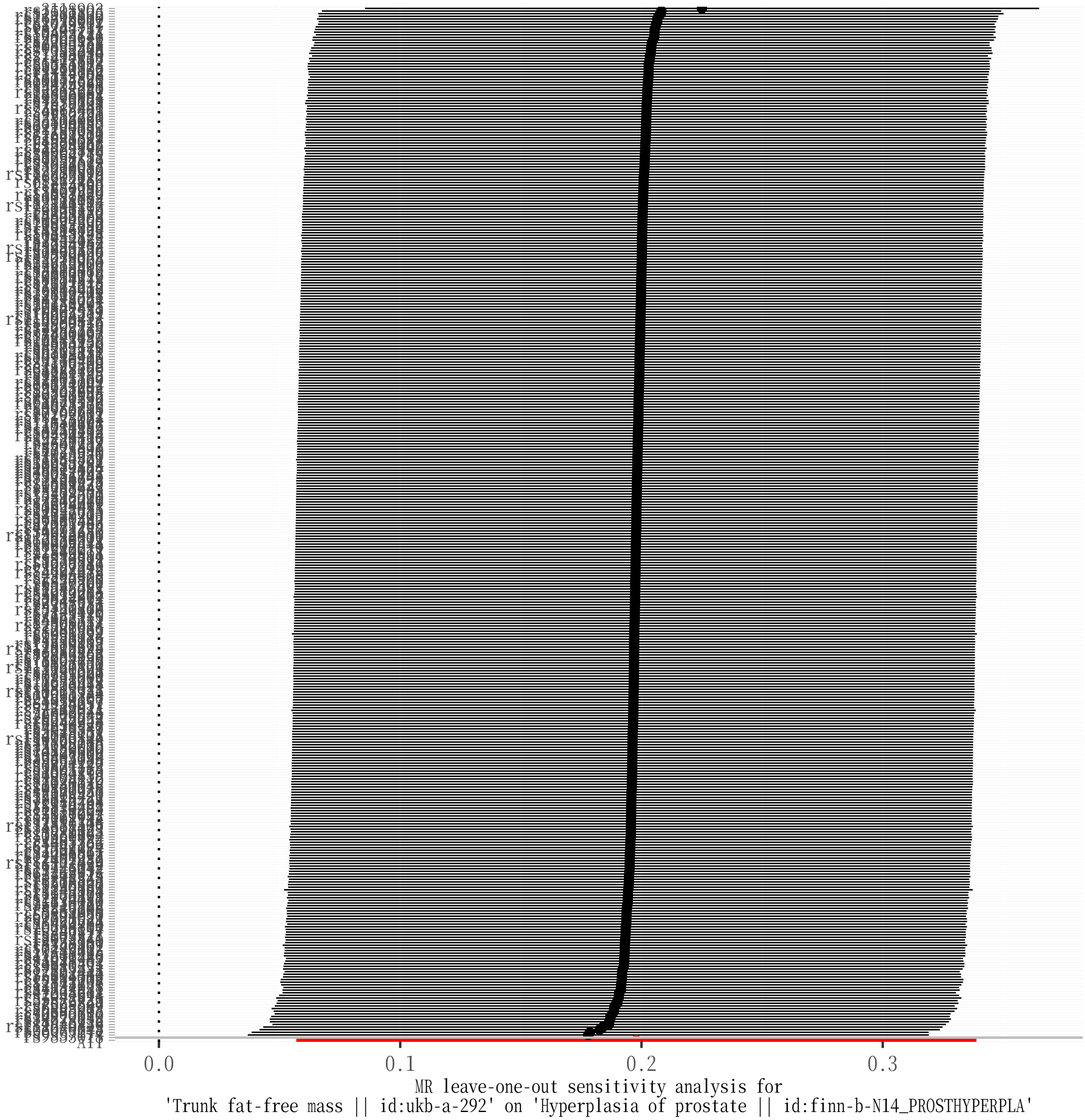
All
0.0
0.1
0.2
0.3
MR leave-one-out sensitivity analysis for
'Trunk fat-free mass || id:ukb-a-292' on 'Hyperplasia of prostate || id:finn-b-N14_PROSTHYPERPLA'

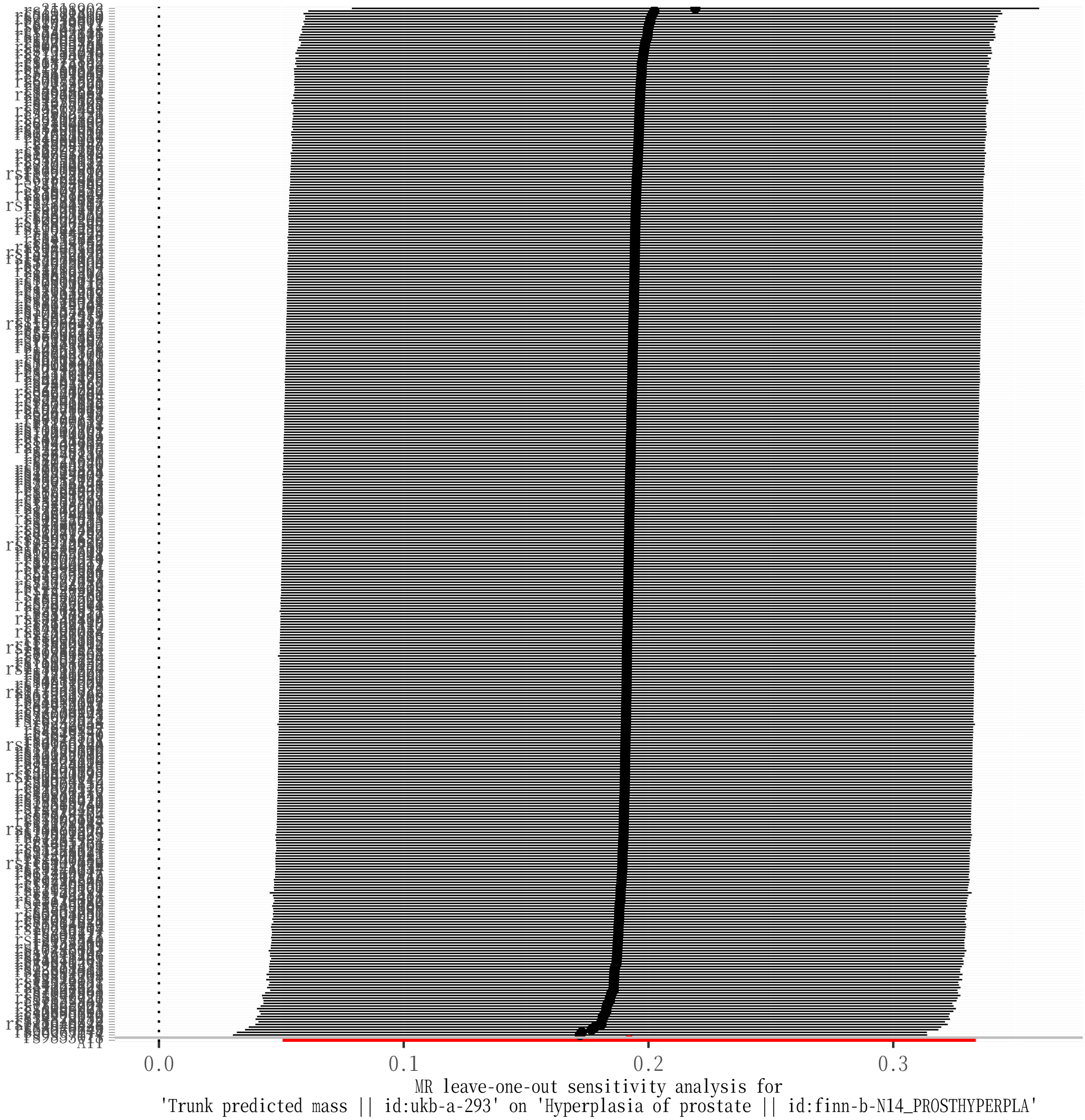
All
0.0
0.1
0.2
0.3
MR leave-one-out sensitivity analysis for
'Trunk predicted mass || id:ukb-a-293' on 'Hyperplasia of prostate || id:finn-b-N14_PROSTHYPERPLA'
