# Supplementary material for: The causal relationship between sarcopenic obesity factors and benign prostate hyperplasia
Source: Front Endocrinol (Lausanne). 2023 Nov 8;14:1290639. doi: 10.3389/fendo.2023.1290639 (PMC10663947; doi:10.3389/fendo.2023.1290639)
Supplement: Supplementary file 3 [file DataSheet_4.pdf]

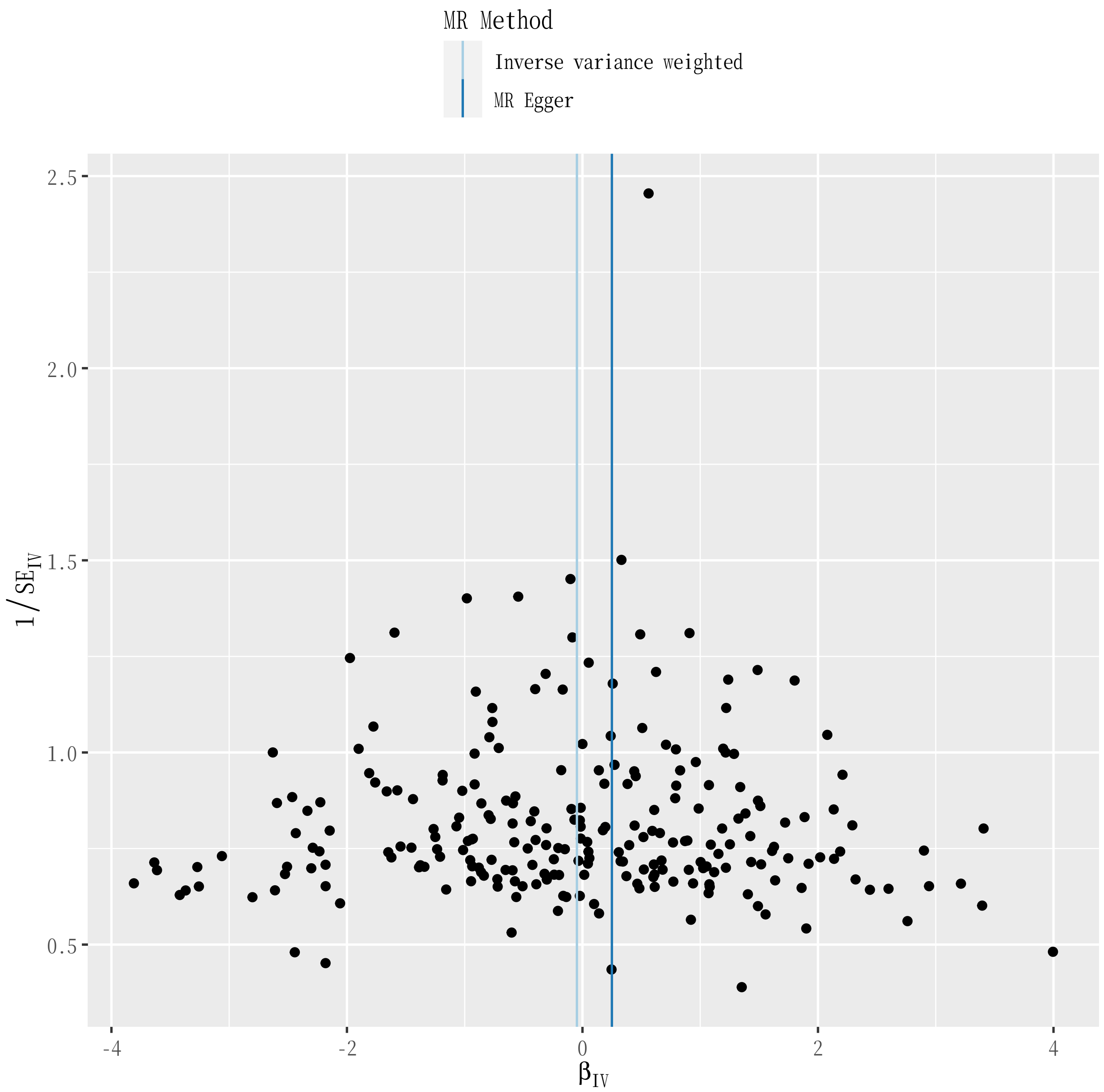
MR Method
Inverse variance weighted
MR Egger
2.5
2.0
1.5
1.0
0.5
$1/SE_{IV}$
-4
-2
0
2
4
β_{IV}

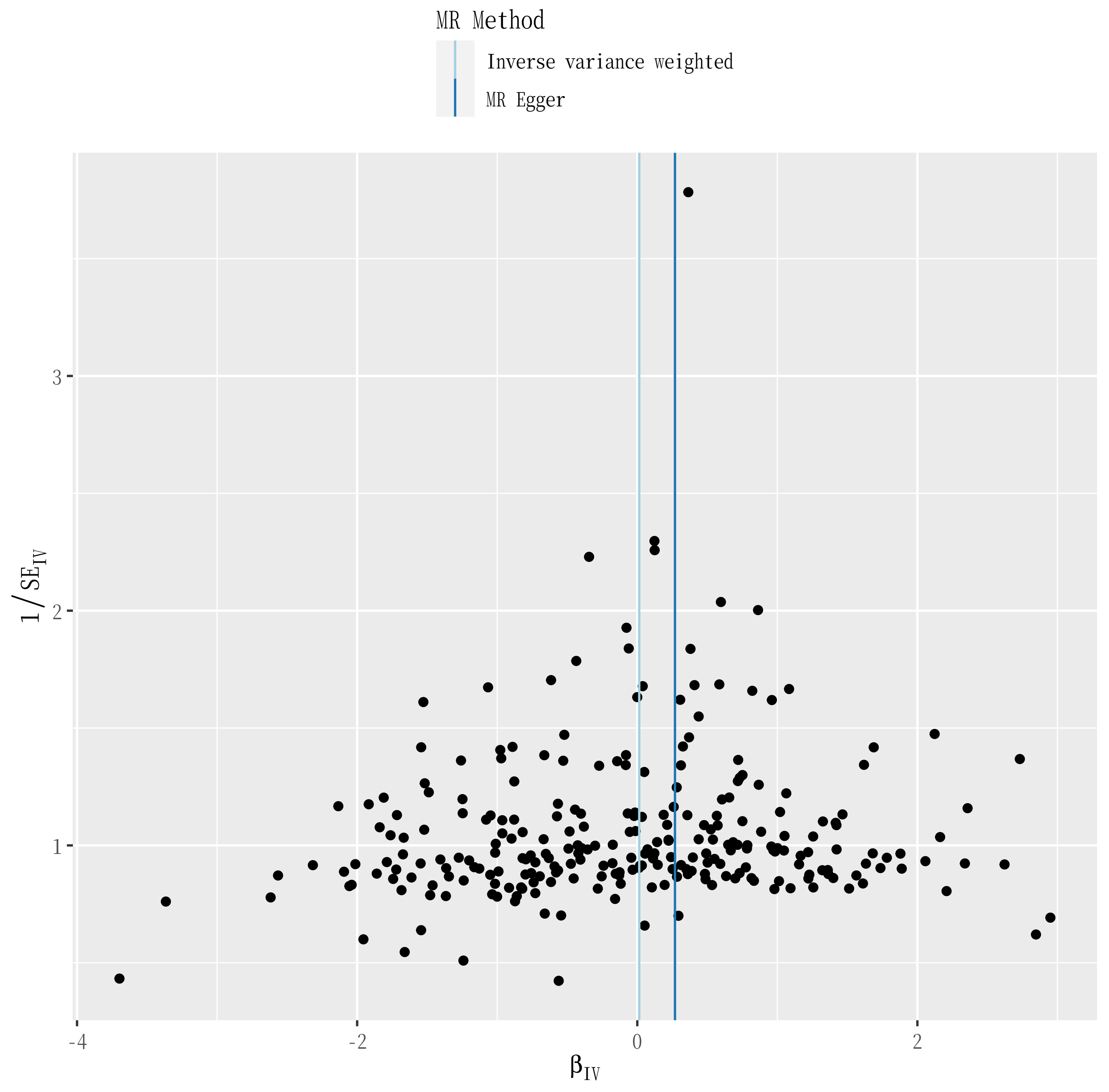
MR Method
Inverse variance weighted
MR Egger
1/SE_IV
3
2
1
-4
-2
0
2
β_IV

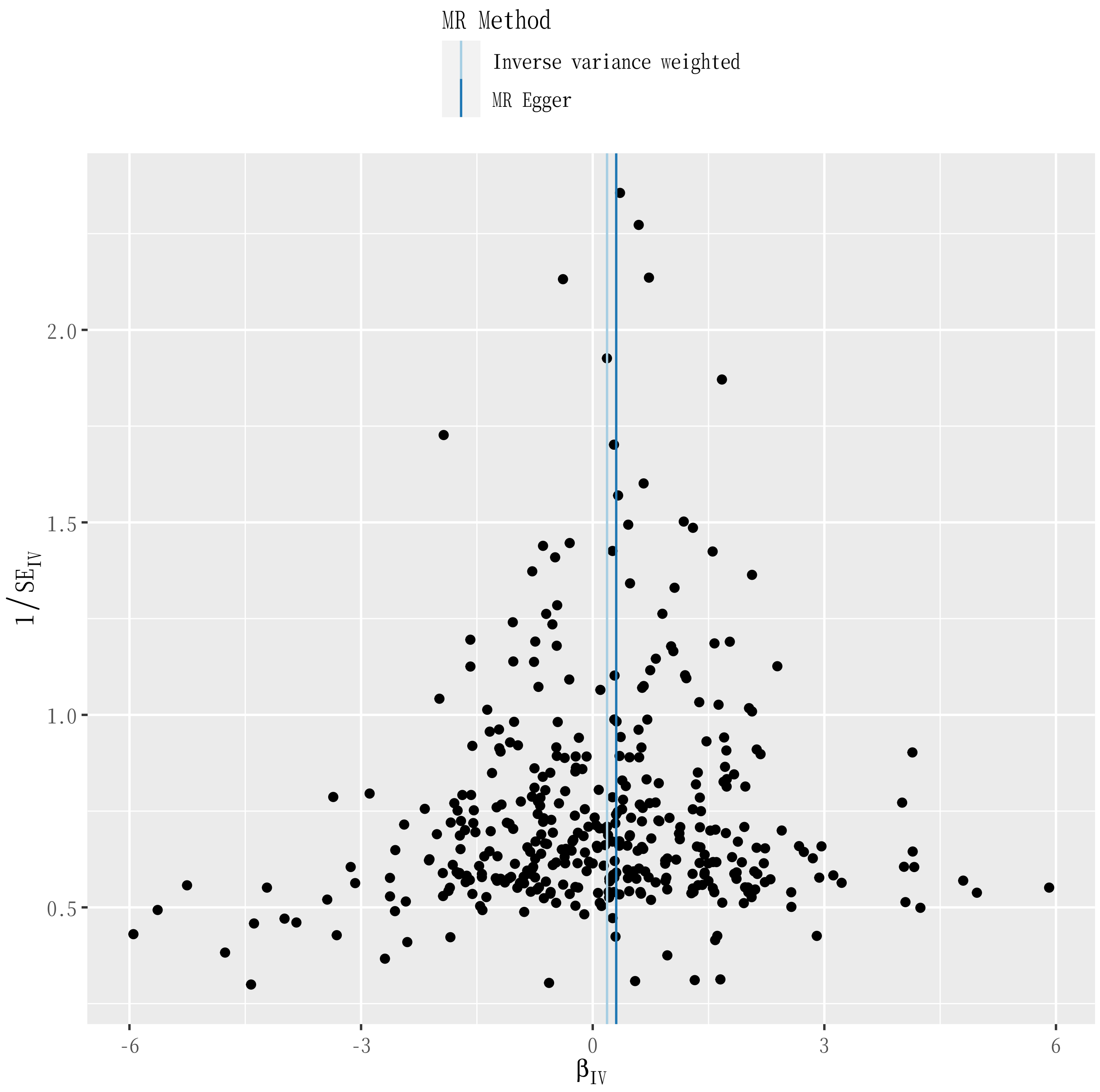

MR Method
Inverse variance weighted
MR Egger
2.0
1.5
1.0
0.5
-6
-3
0
3
6
1/SE_IV
β_IV

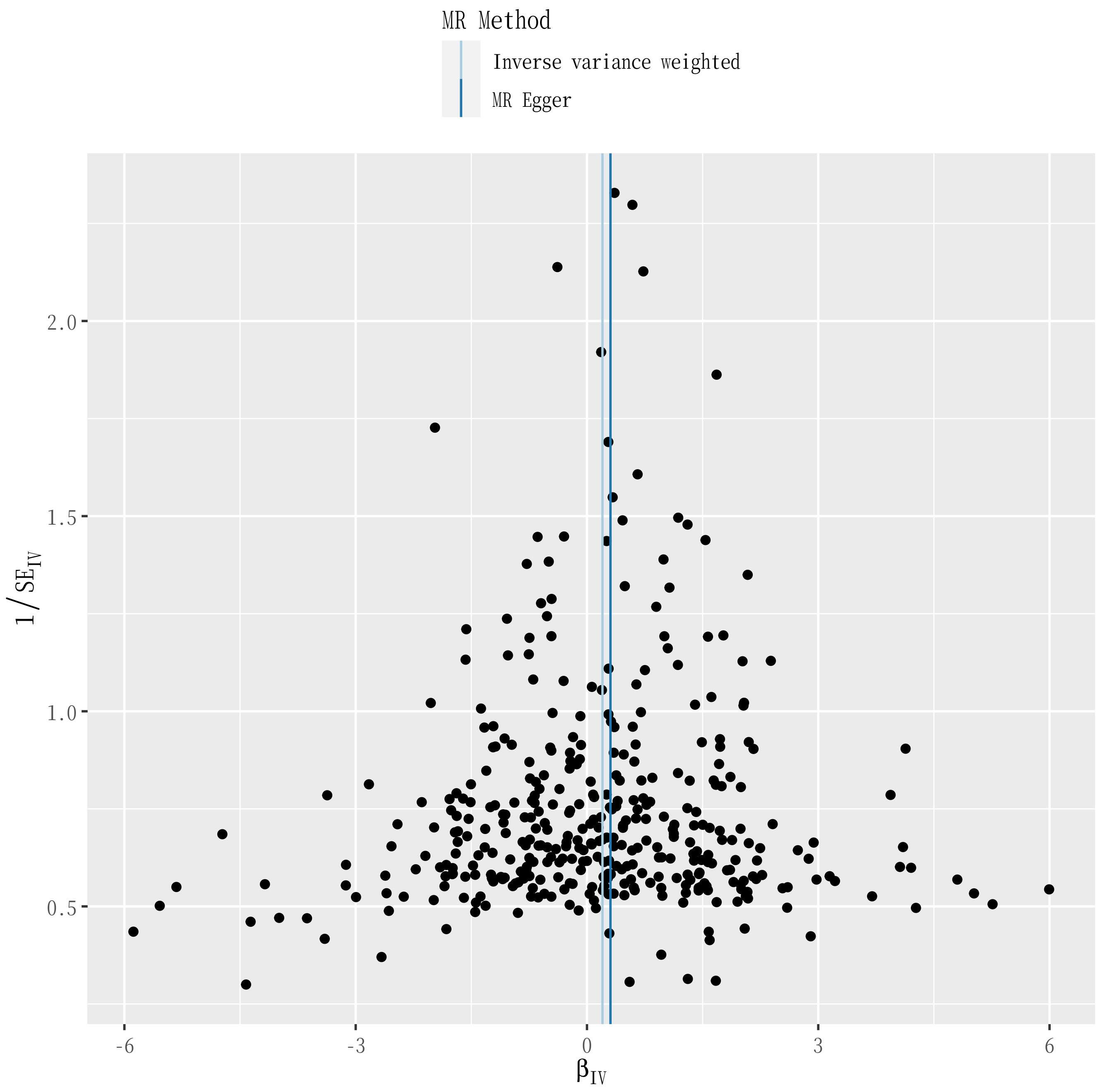
MR Method
Inverse variance weighted
MR Egger
1/SE_IV
2.0
1.5
1.0
0.5
-6
-3
0
3
6
β_IV

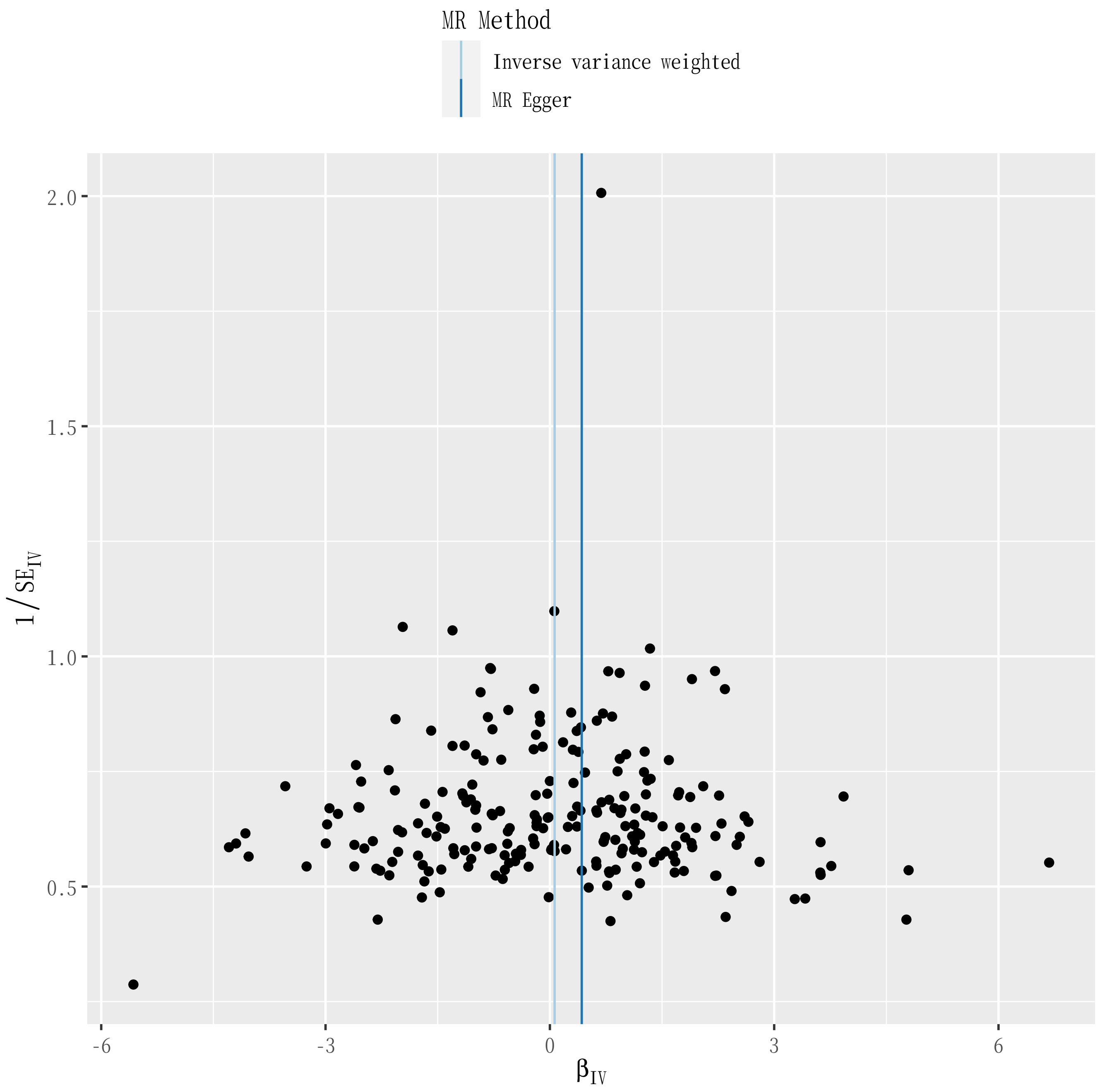
MR Method
Inverse variance weighted
MR Egger
2.0
1.5
1.0
0.5
1/SE_IV
-6
-3
0
3
6
β_IV

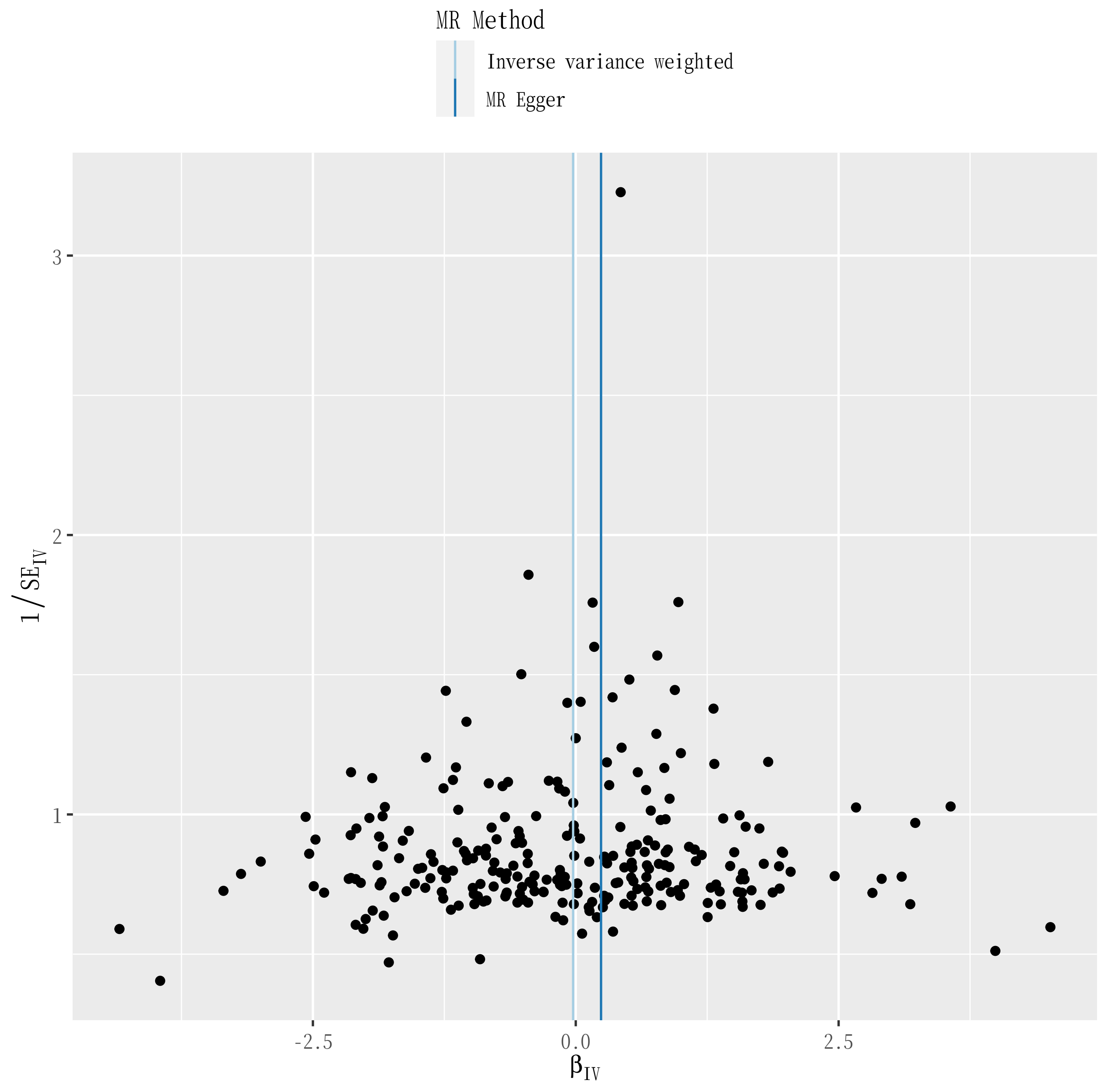
MR Method
Inverse variance weighted
MR Egger
3
2
1
$1/SE_{IV}$
-2.5
0.0
2.5
β_{IV}

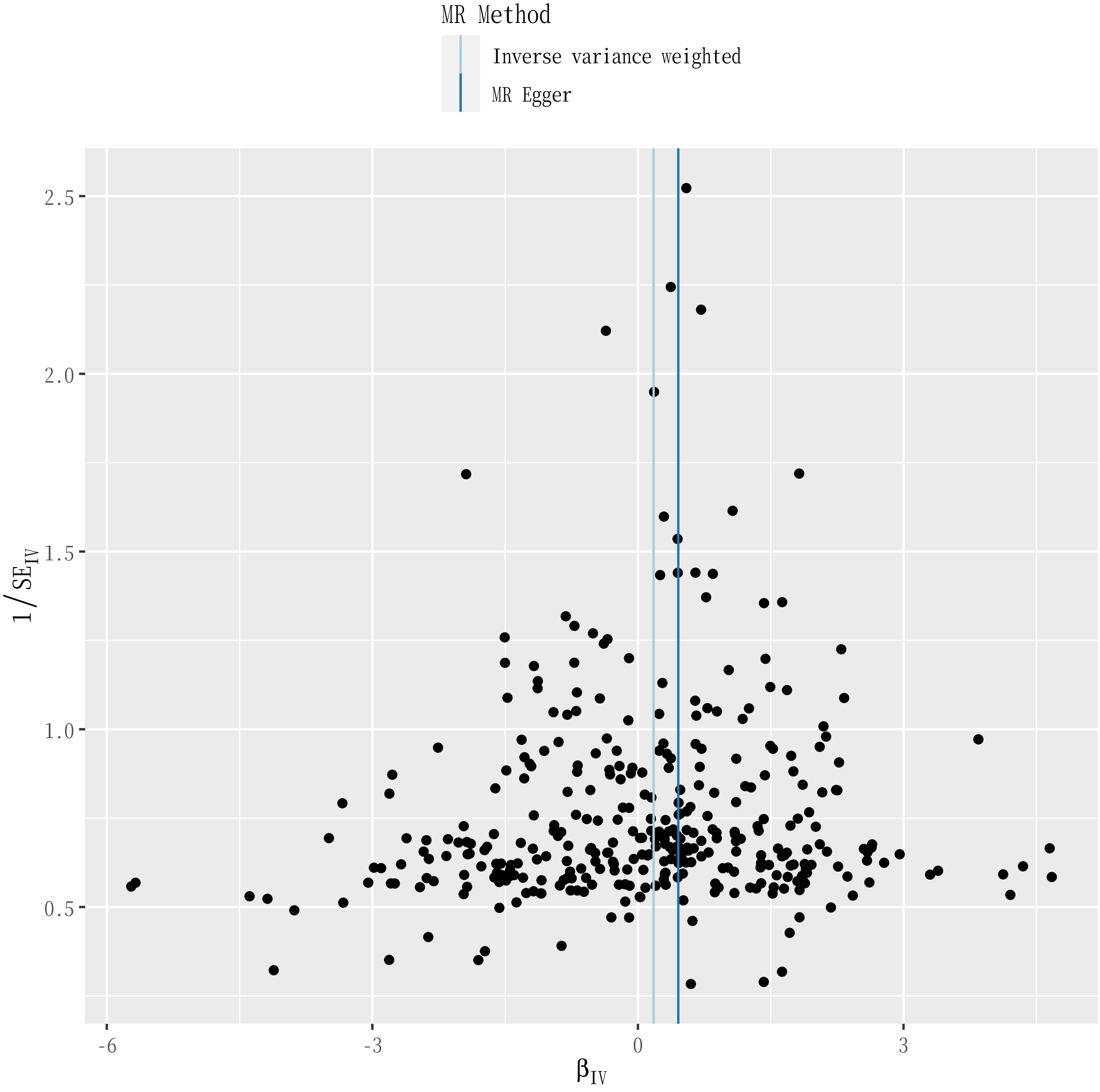

MR Method
Inverse variance weighted
MR Egger
2.5
2.0
1.5
1.0
0.5
1/SE_IV
-6
-3
0
3
β_IV

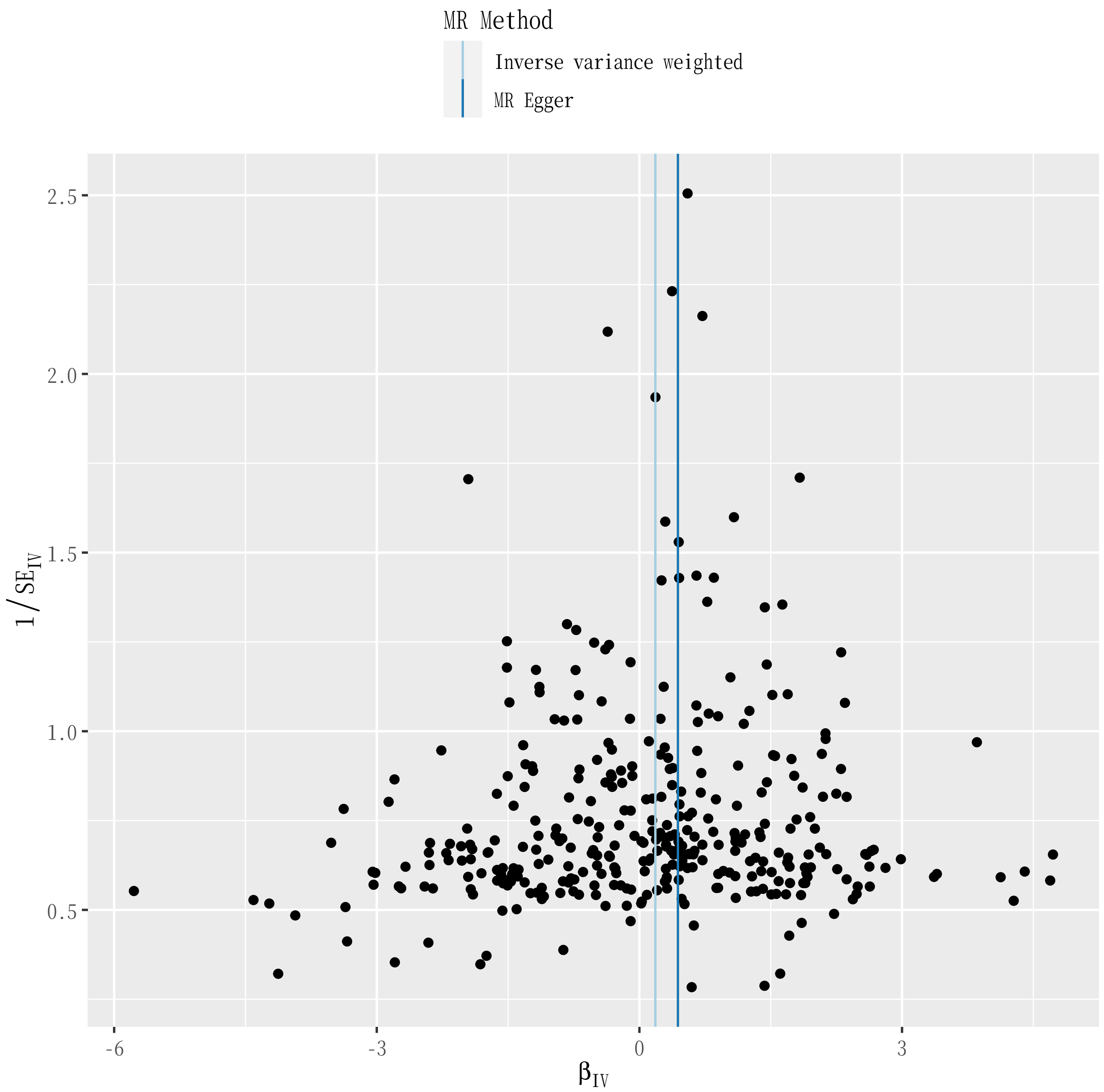
MR Method
Inverse variance weighted
MR Egger
2.5
2.0
1.5
1.0
0.5
1/SE$_{IV}$
-6
-3
0
3
β$_{IV}$

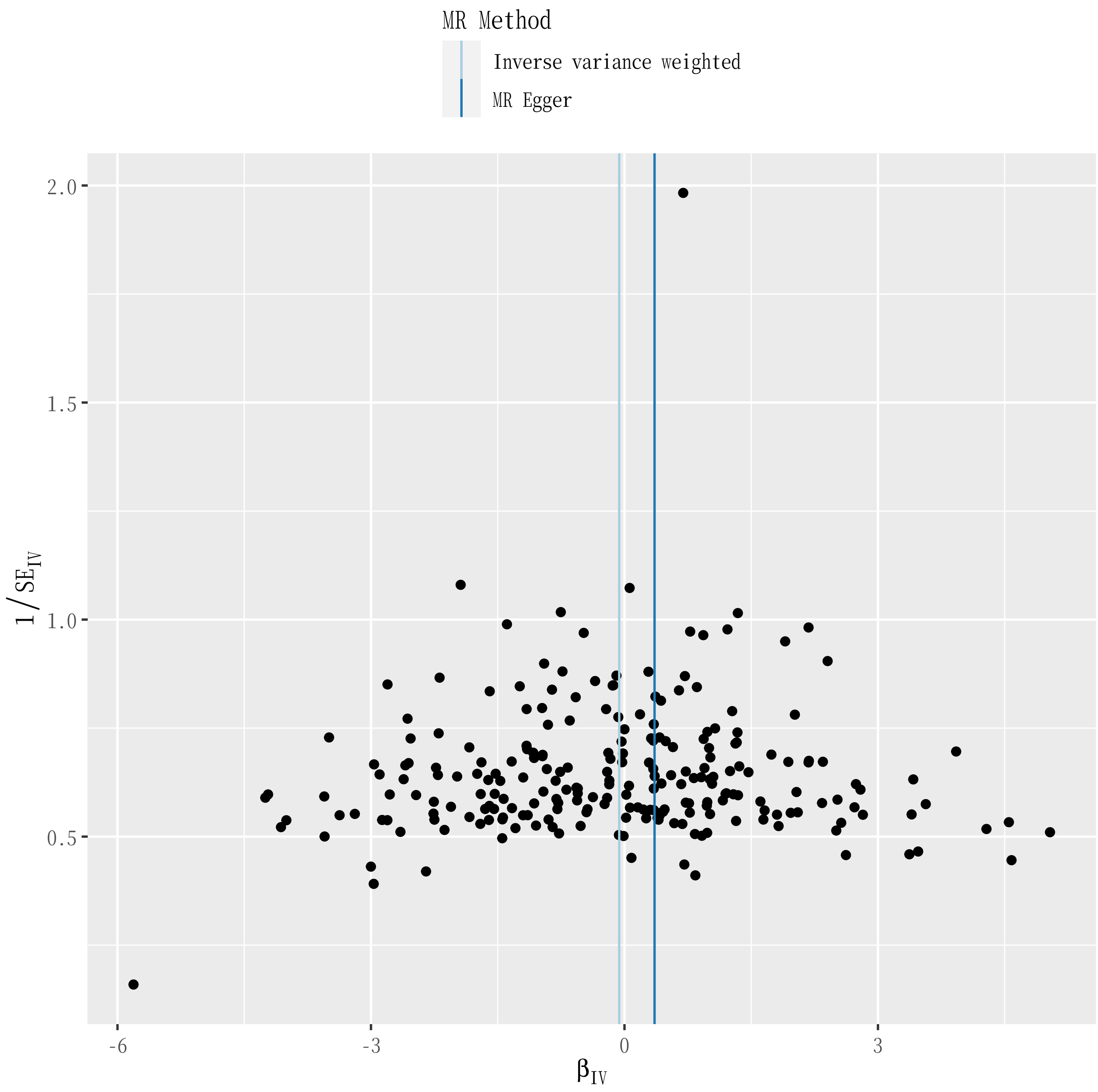

MR Method
Inverse variance weighted
MR Egger
2.0
1.5
1.0
0.5
$1/SE_{IV}$
-6
-3
0
3
β_{IV}

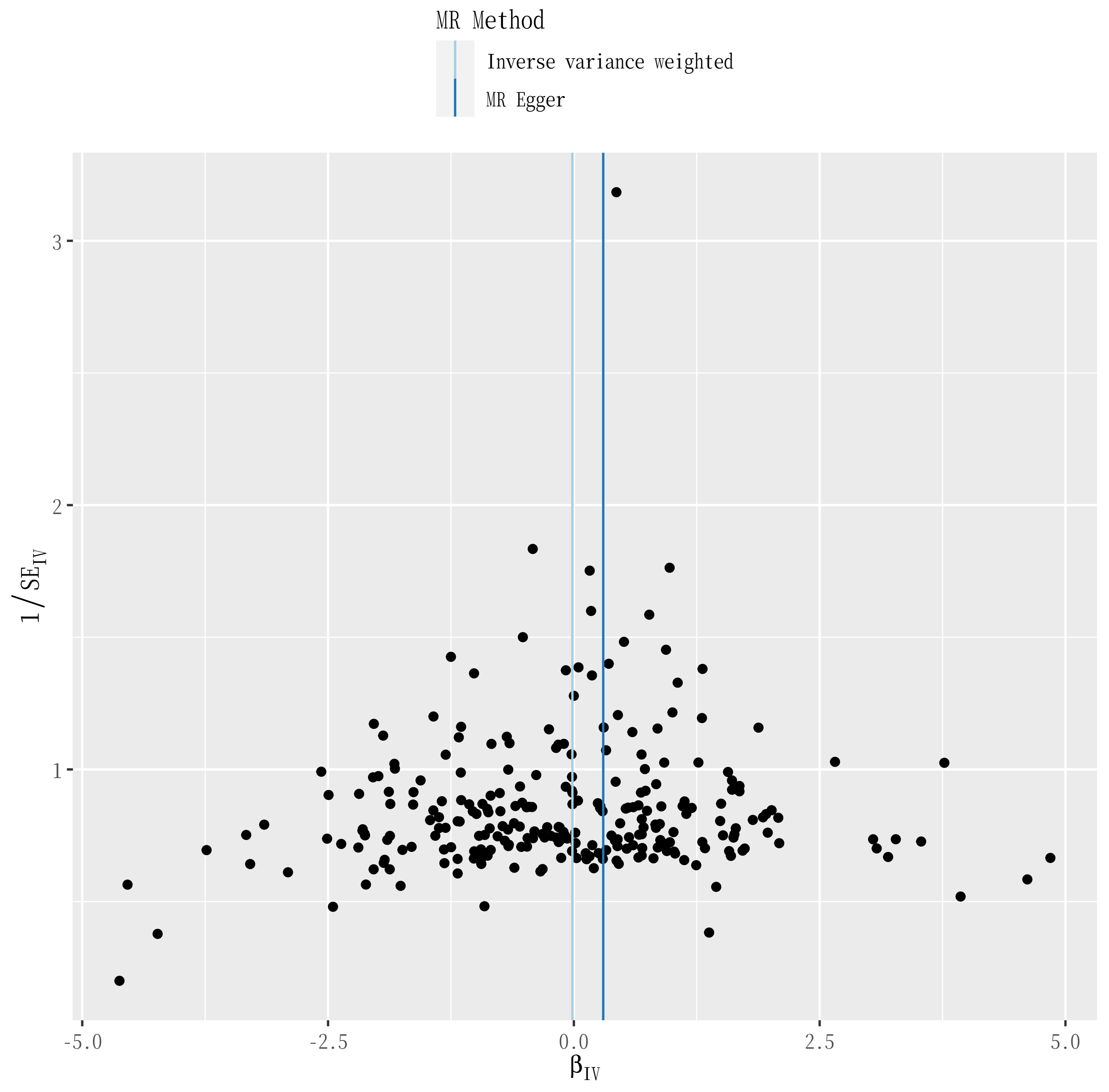
MR Method
Inverse variance weighted
MR Egger
1/SE_IV
3
2
1
-5.0
-2.5
0.0
2.5
5.0
β_IV

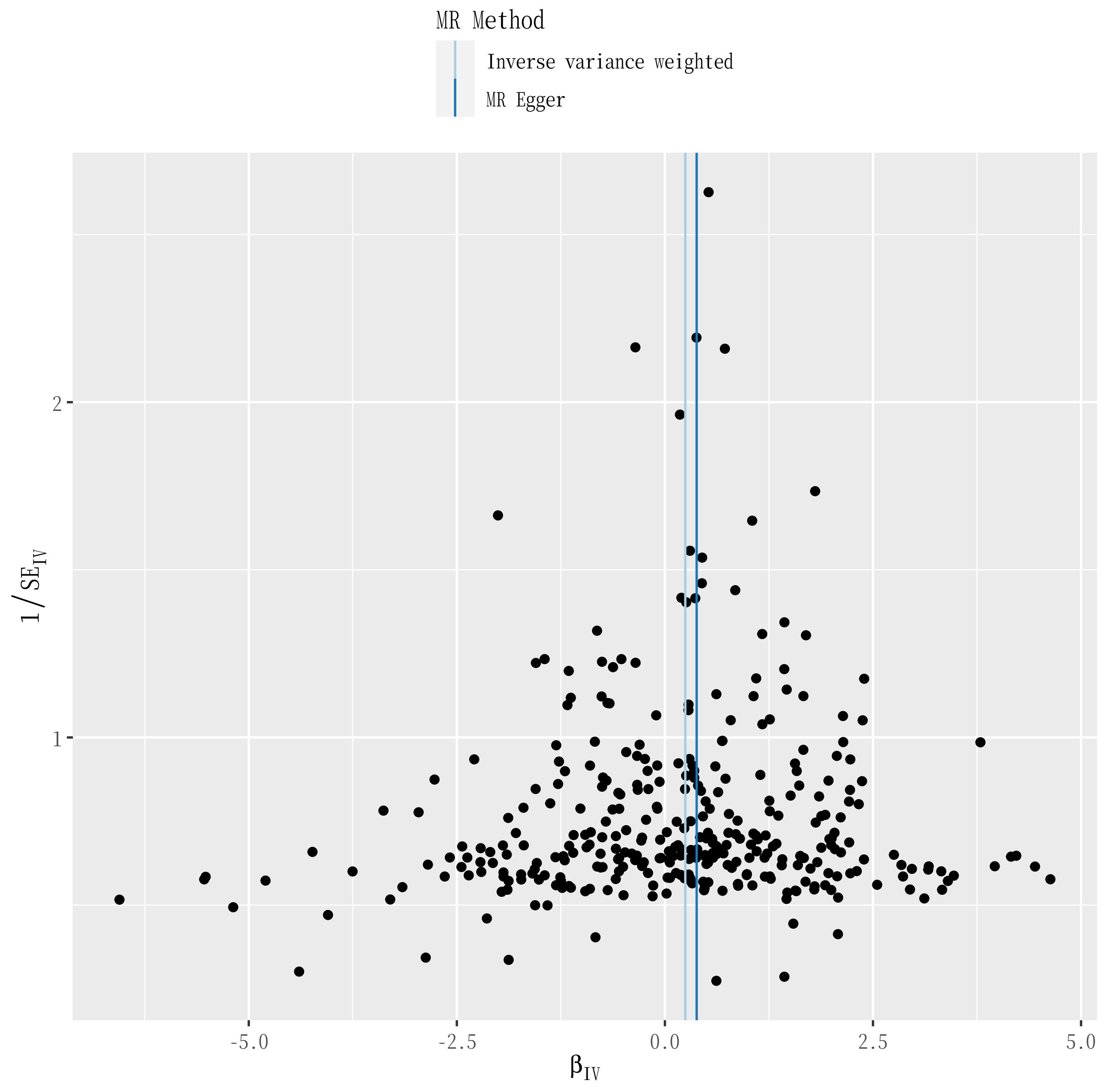
MR Method
Inverse variance weighted
MR Egger
2
1
1/SE_IV
-5.0
-2.5
0.0
2.5
5.0
β_IV

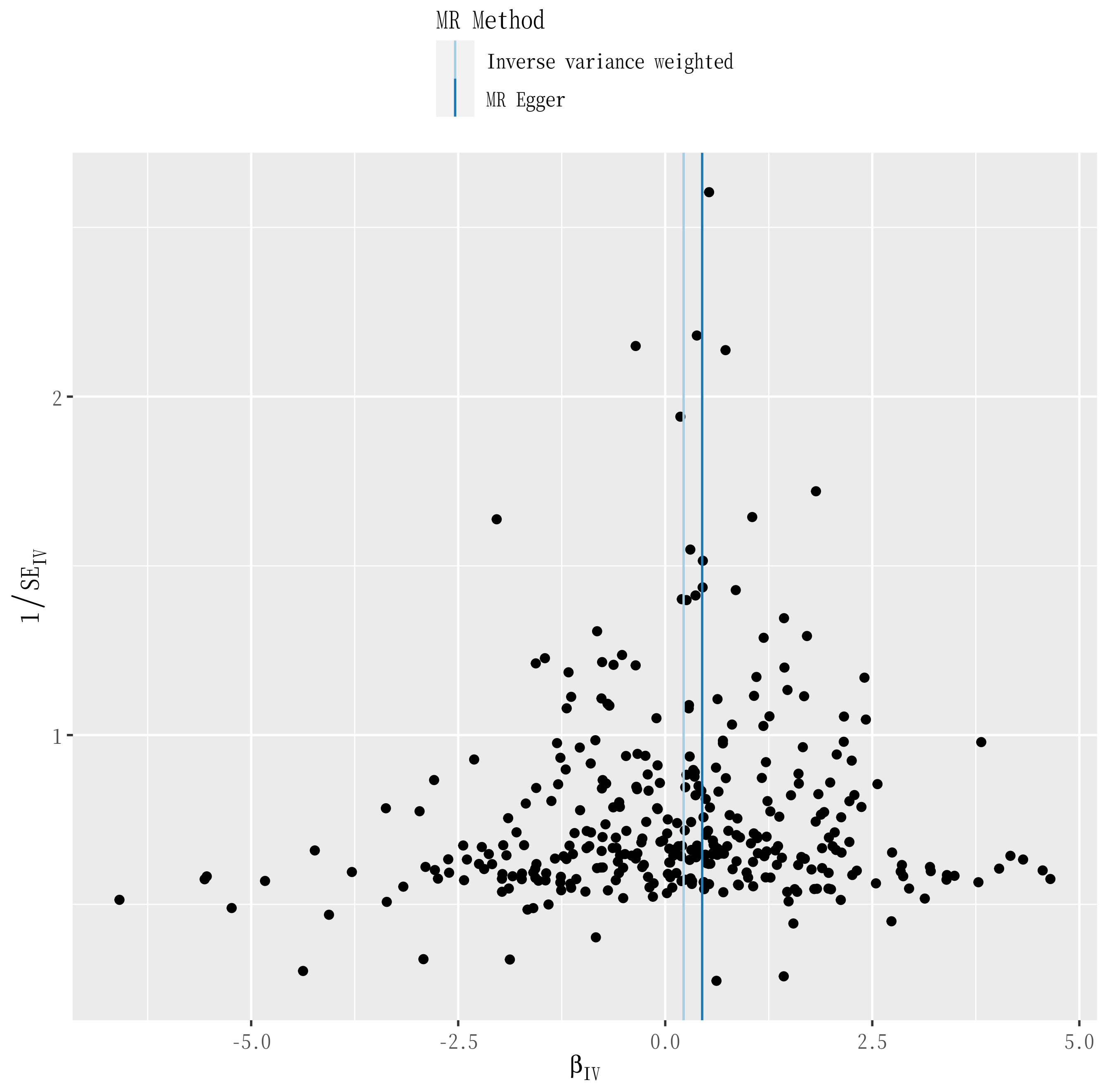
MR Method
Inverse variance weighted
MR Egger
2
1
$1/SE_{IV}$
-5.0
-2.5
0.0
2.5
5.0
β_{IV}

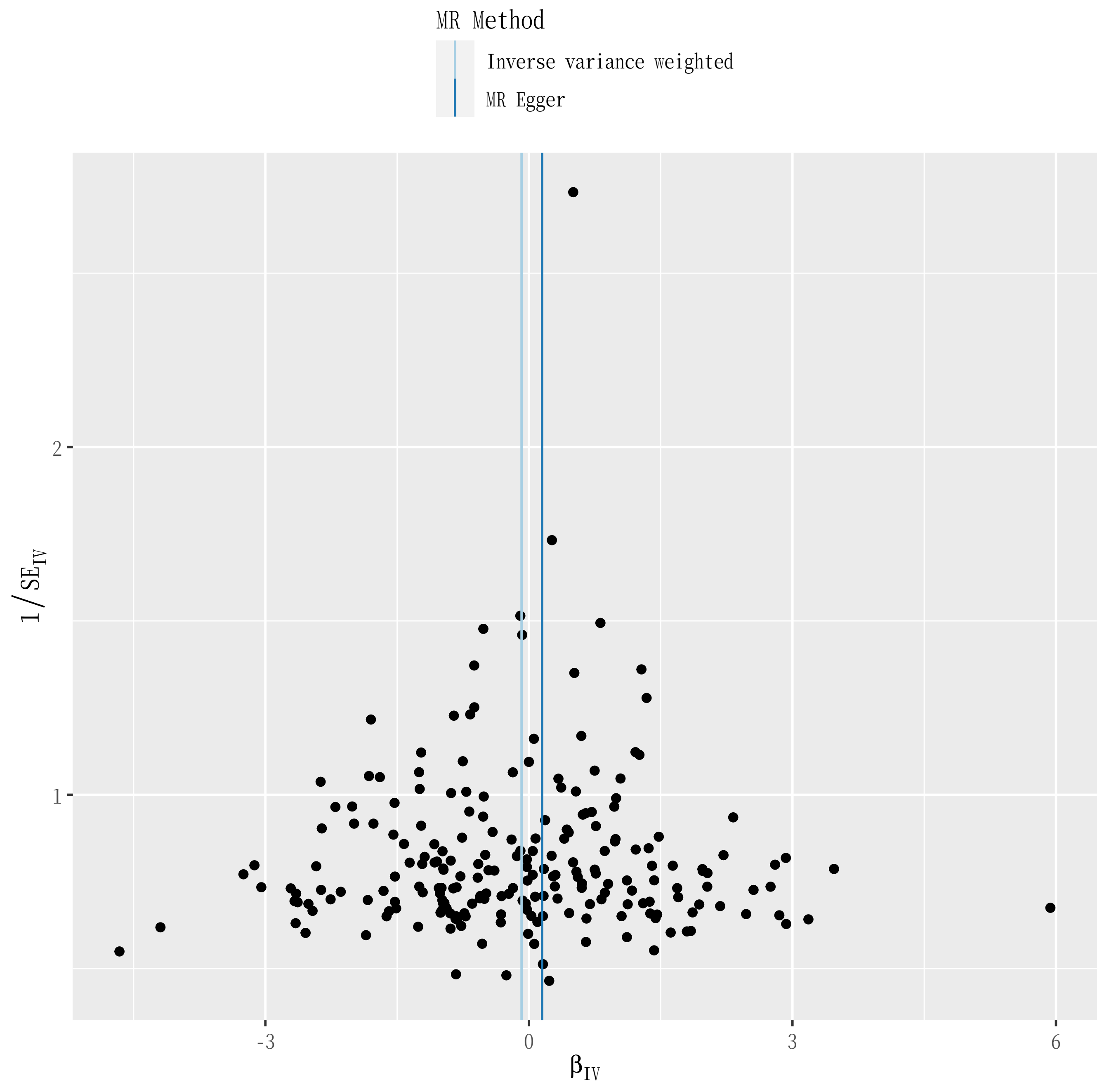

MR Method
Inverse variance weighted
MR Egger
1/SE_IV
2
1
-3
0
3
6
β_IV

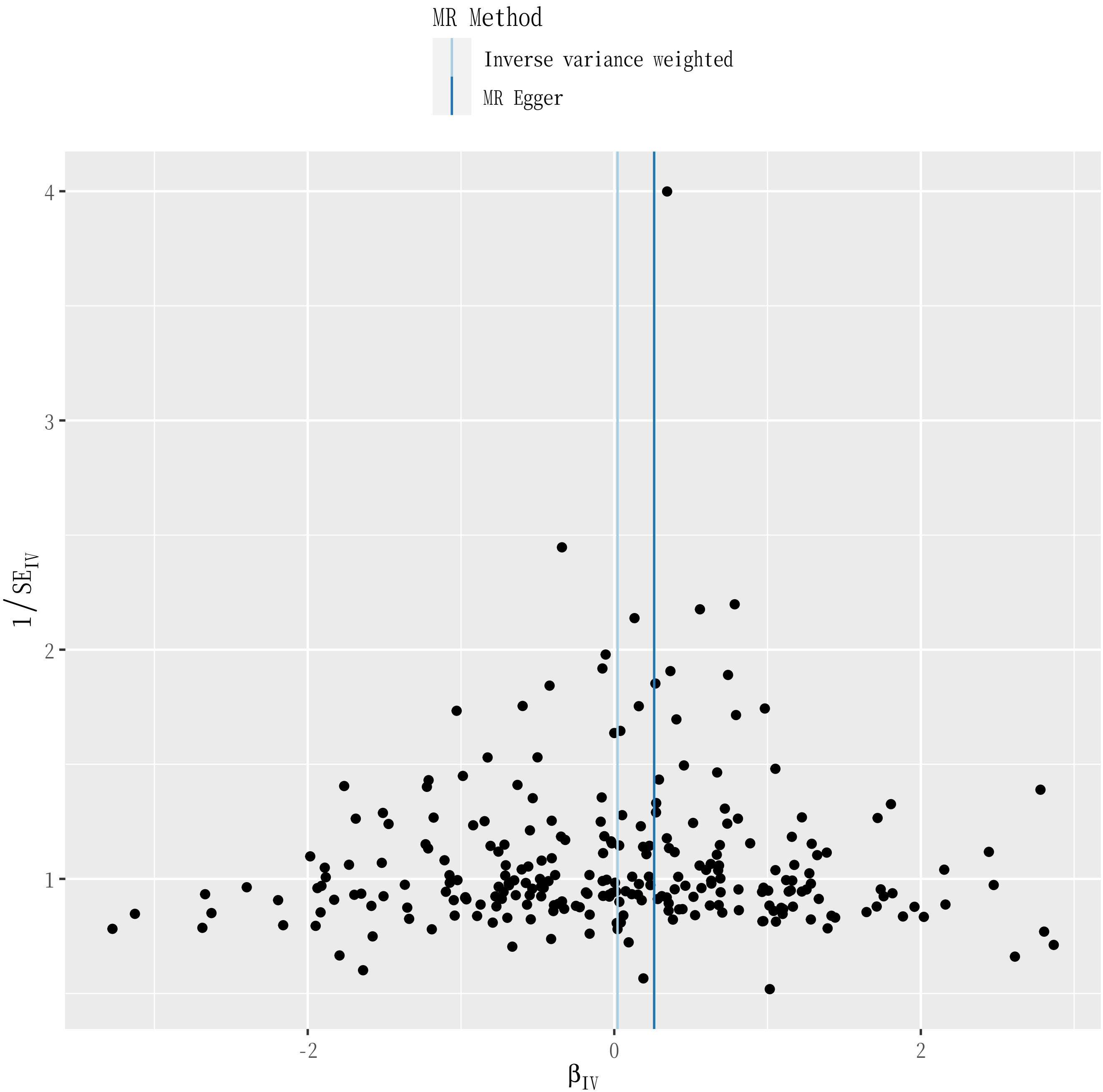
MR Method
Inverse variance weighted
MR Egger
4
3
2
1
-2
0
2
$1/SE_{IV}$
β_{IV}

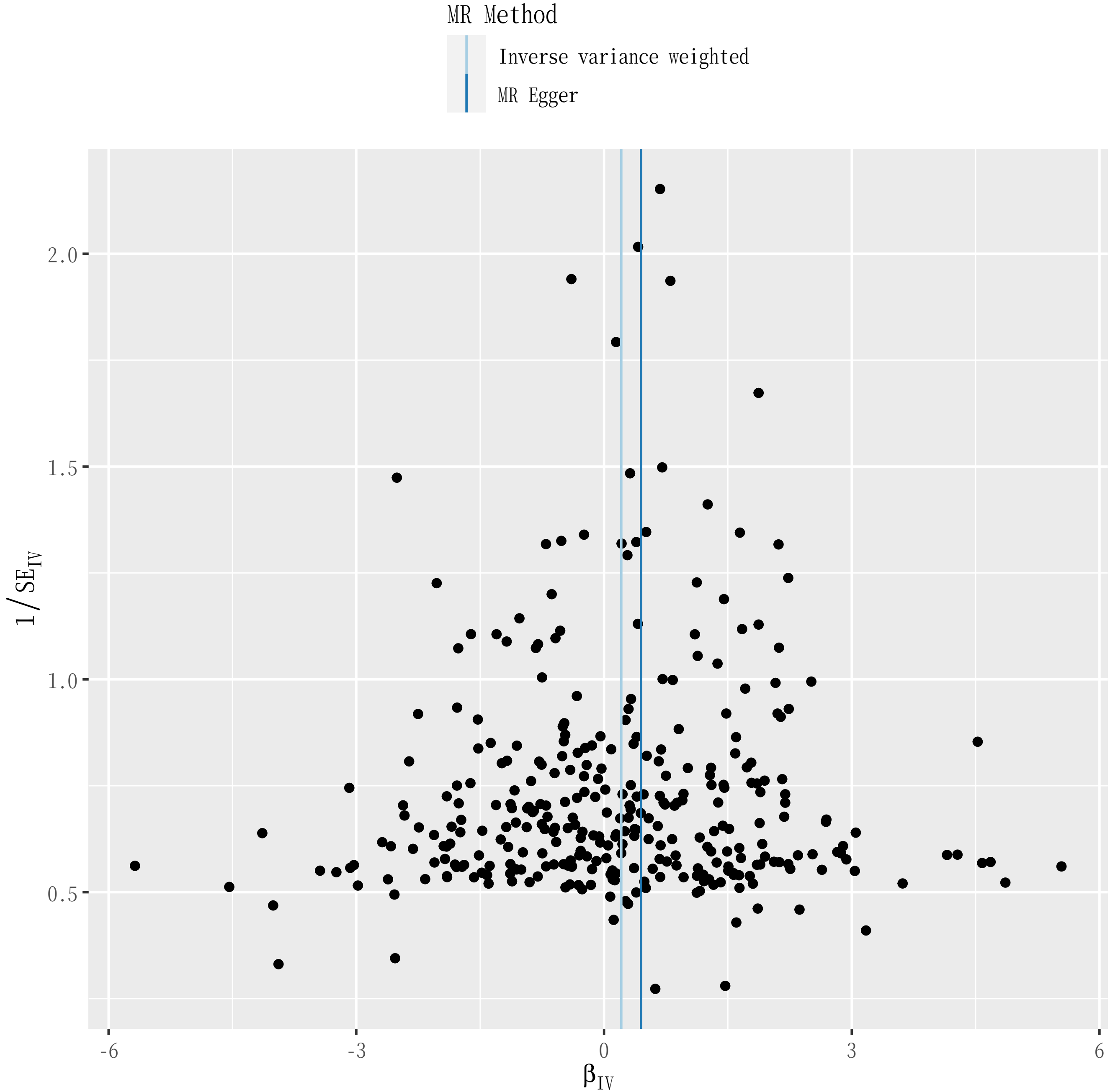

MR Method
Inverse variance weighted
MR Egger
2.0
1.5
1.0
0.5
$1/SE_{IV}$
-6
-3
0
3
6
β_{IV}

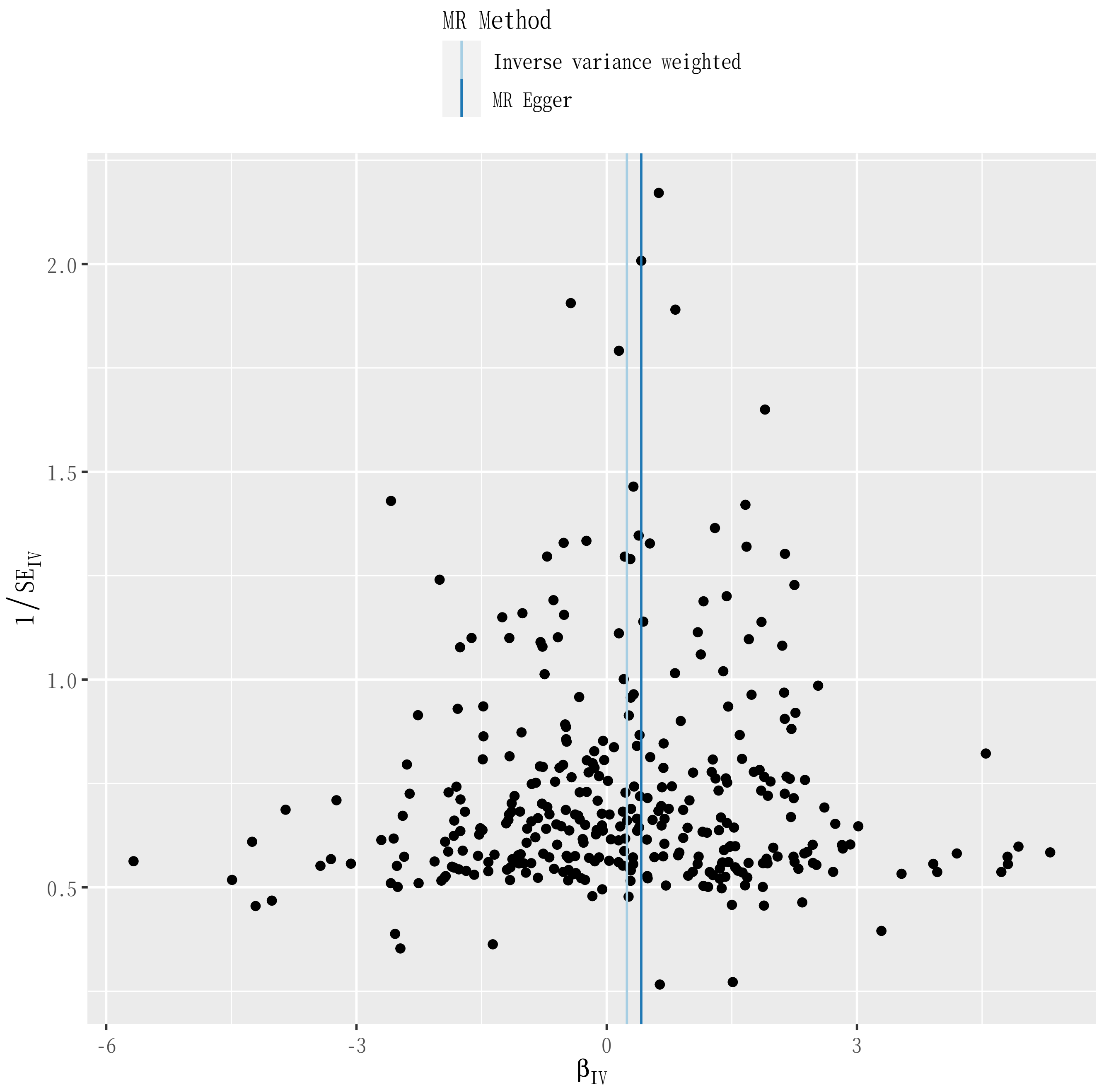

MR Method
Inverse variance weighted
MR Egger
2.0
1.5
1.0
0.5
-6
-3
0
3
1/SE$_{IV}$
β$_{IV}$

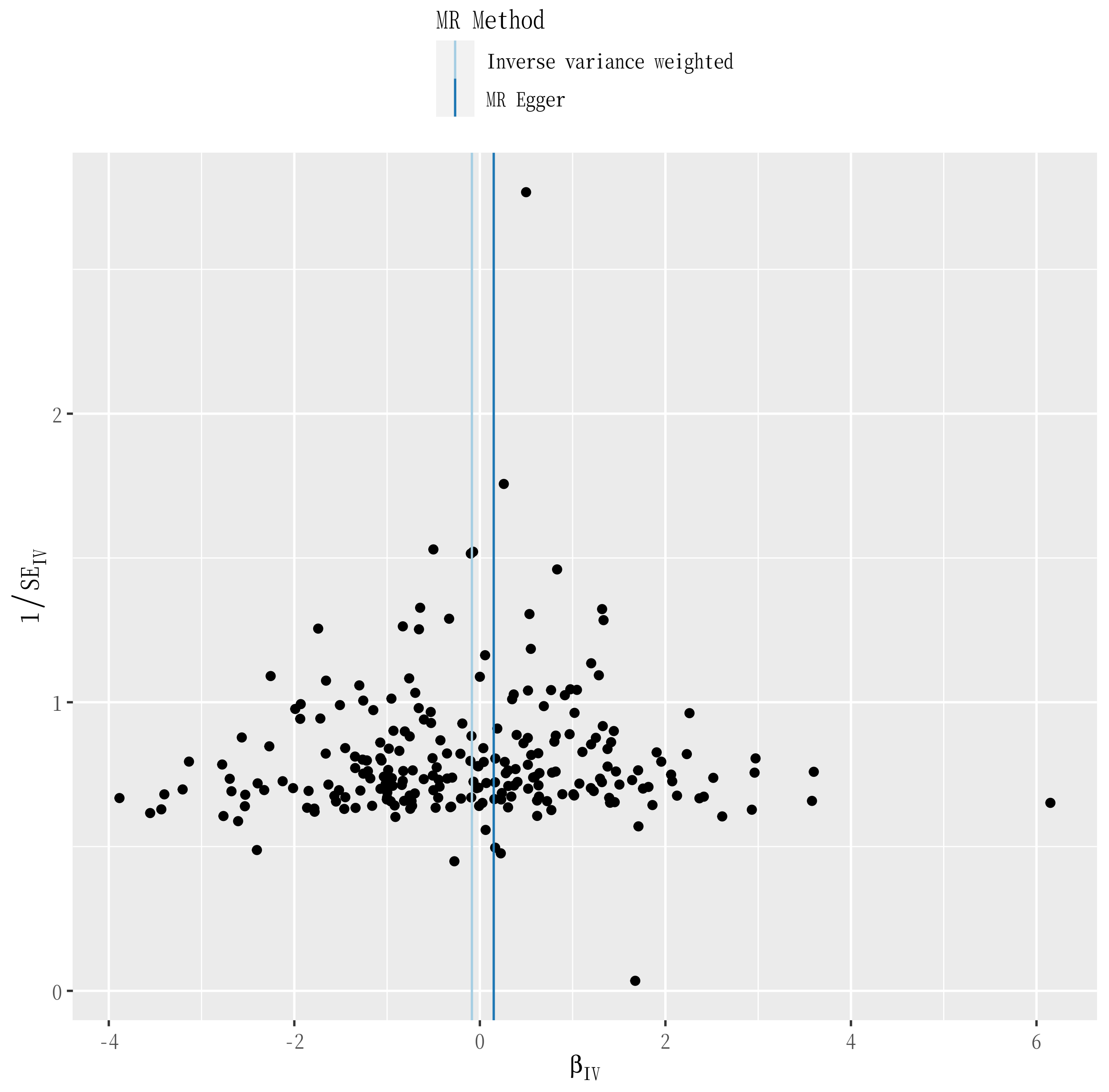
MR Method
Inverse variance weighted
MR Egger
1/SE_IV
β_IV
-4
-2
0
2
4
6
0
1
2

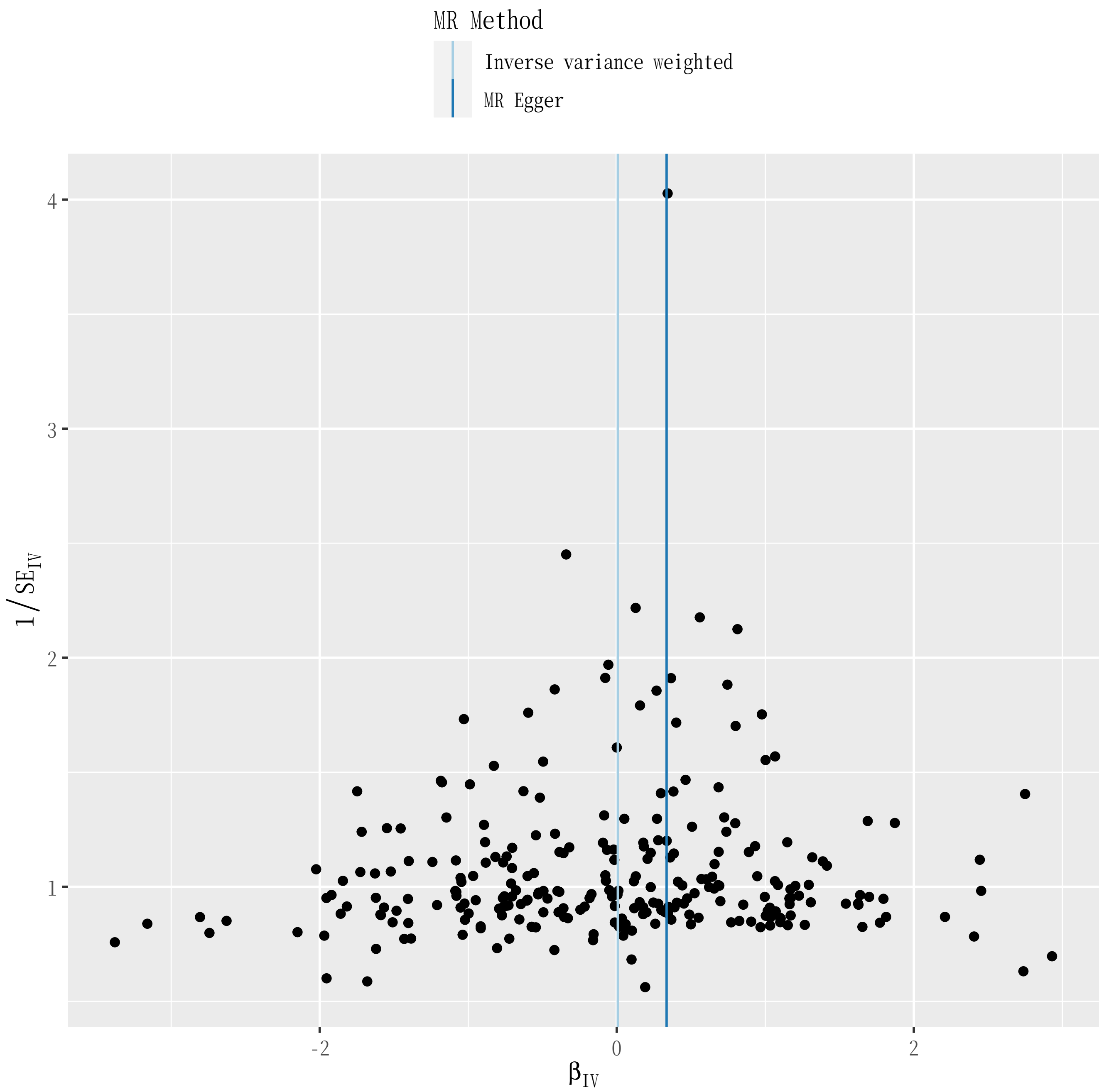

MR Method
Inverse variance weighted
MR Egger
1/SE_IV
4
3
2
1
-2
0
2
β_IV

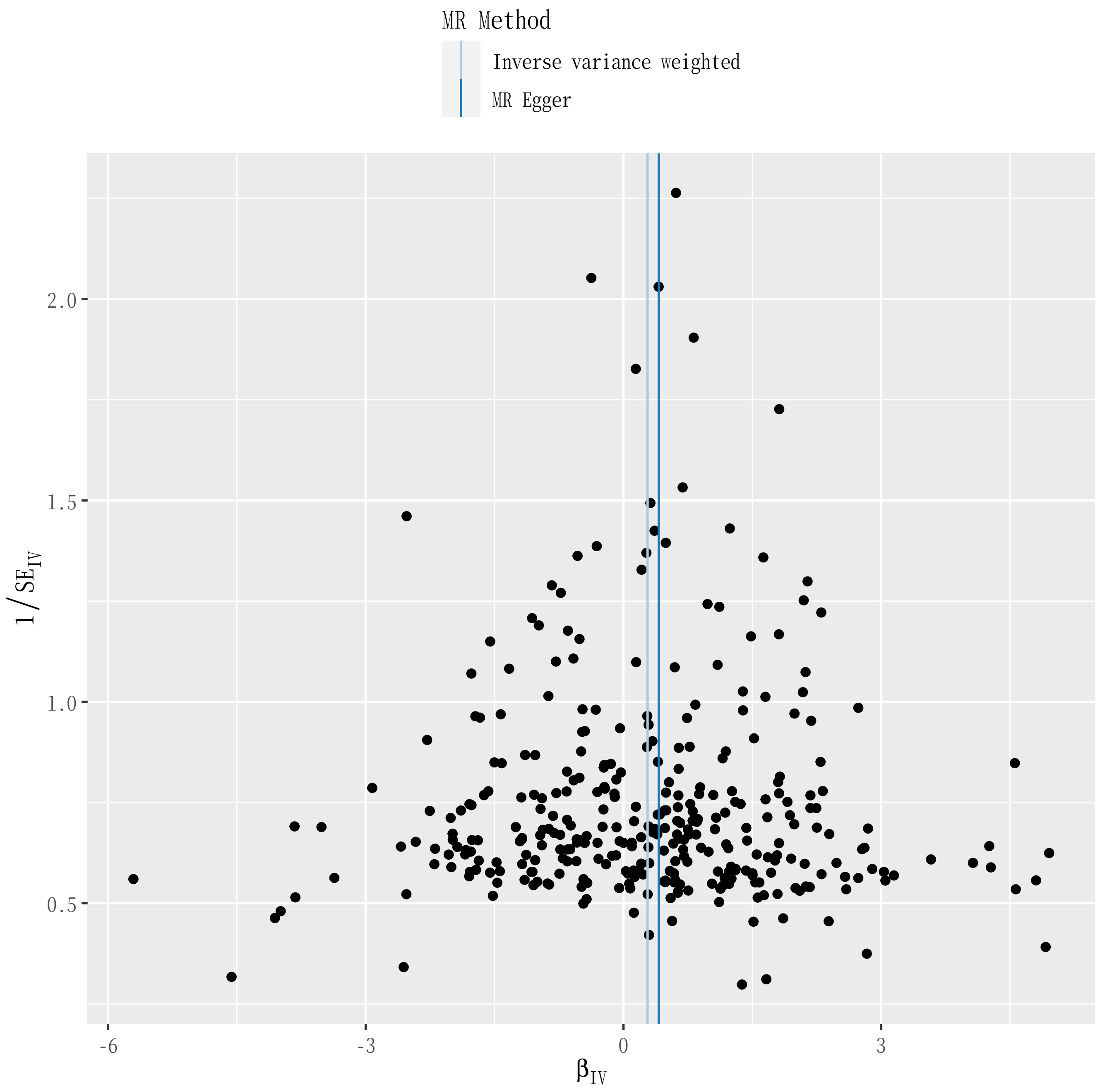

MR Method
Inverse variance weighted
MR Egger
2.0
1.5
1.0
0.5
$1/SE_{IV}$
-6
-3
0
3
β_{IV}

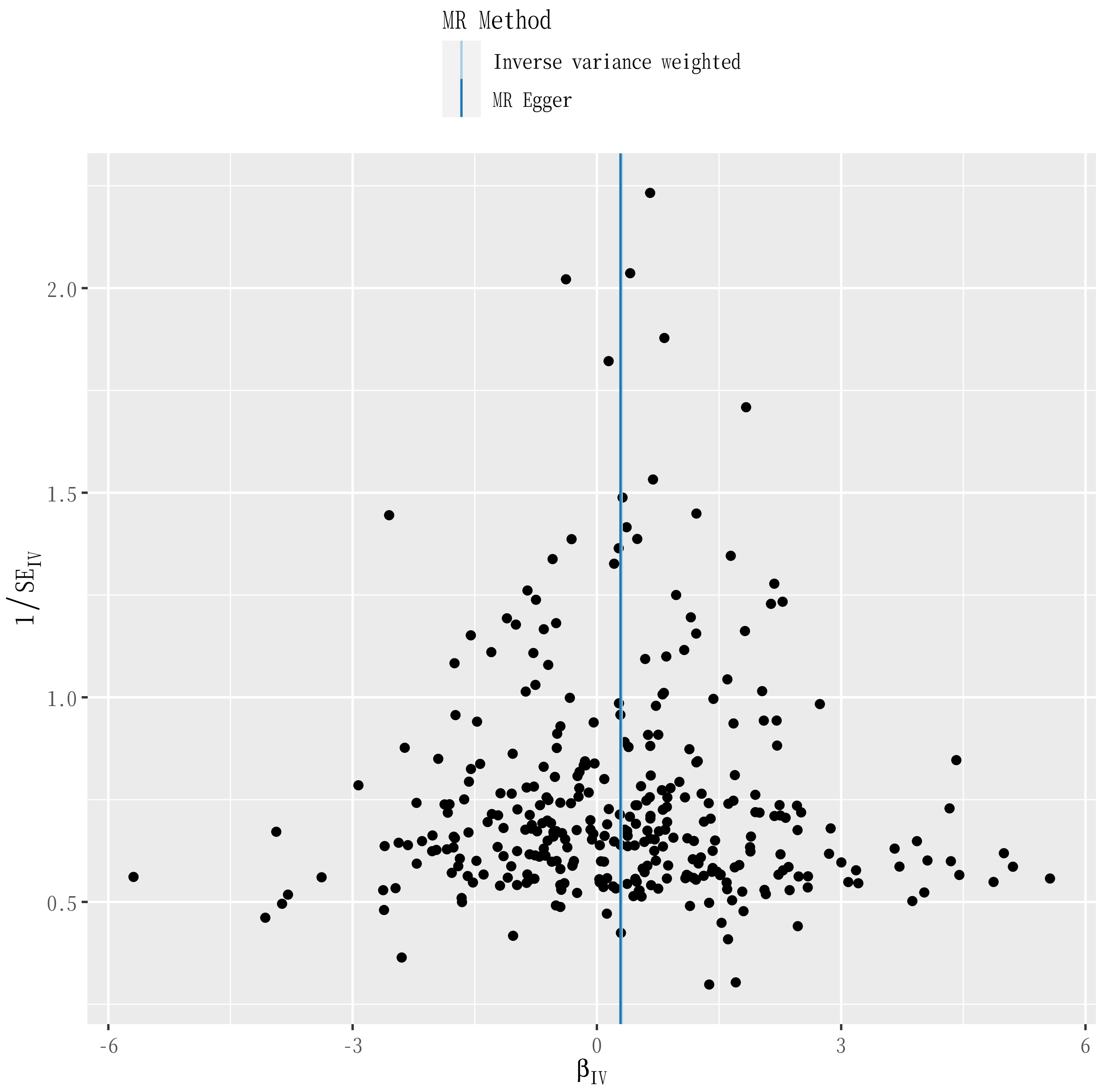
MR Method
Inverse variance weighted
MR Egger
2.0
1.5
1.0
0.5
1/SE_IV
-6
-3
0
3
6
β_IV

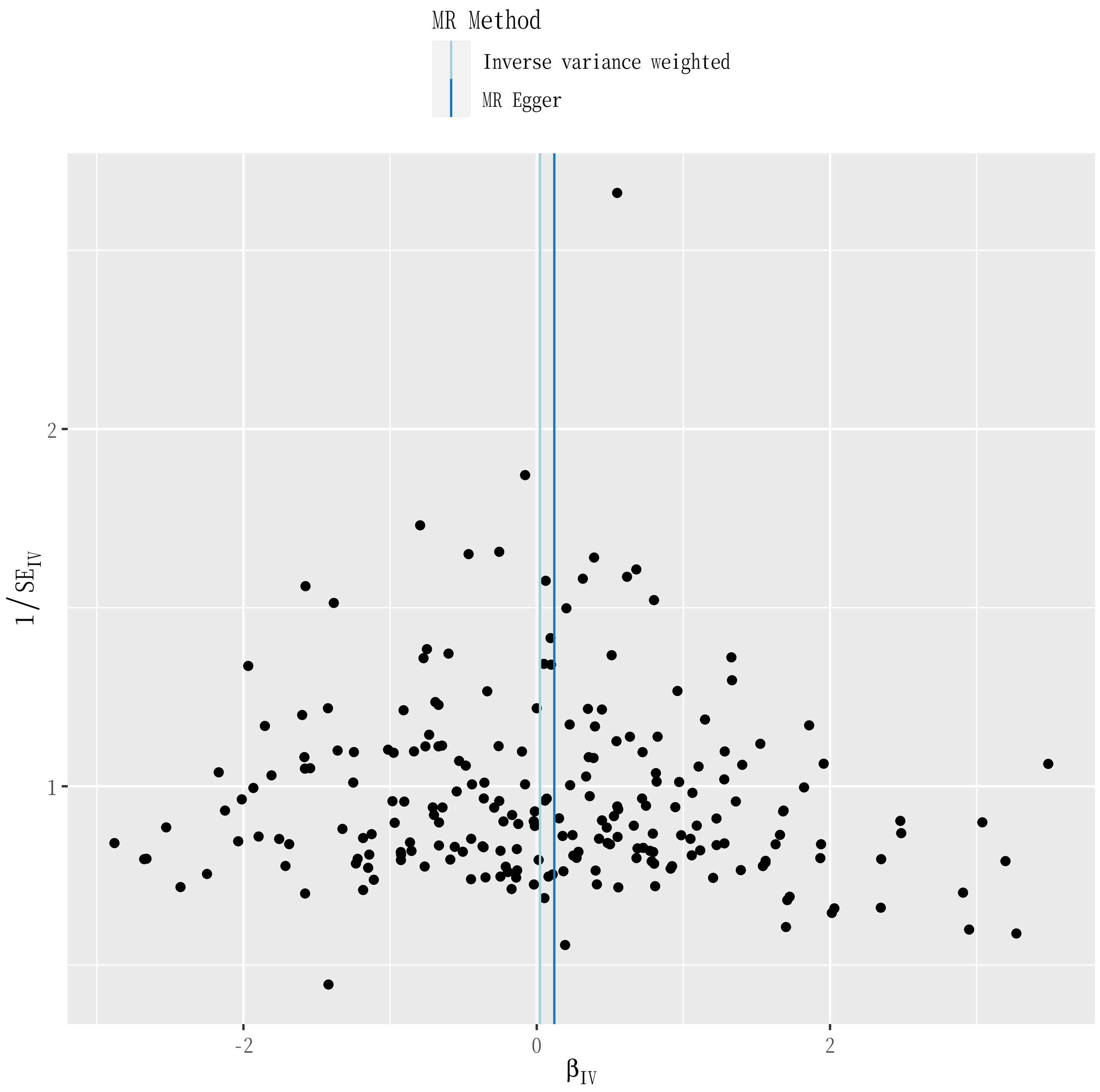
MR Method
Inverse variance weighted
MR Egger
2
1
-2
0
2
$1/SE_{IV}$
β_{IV}

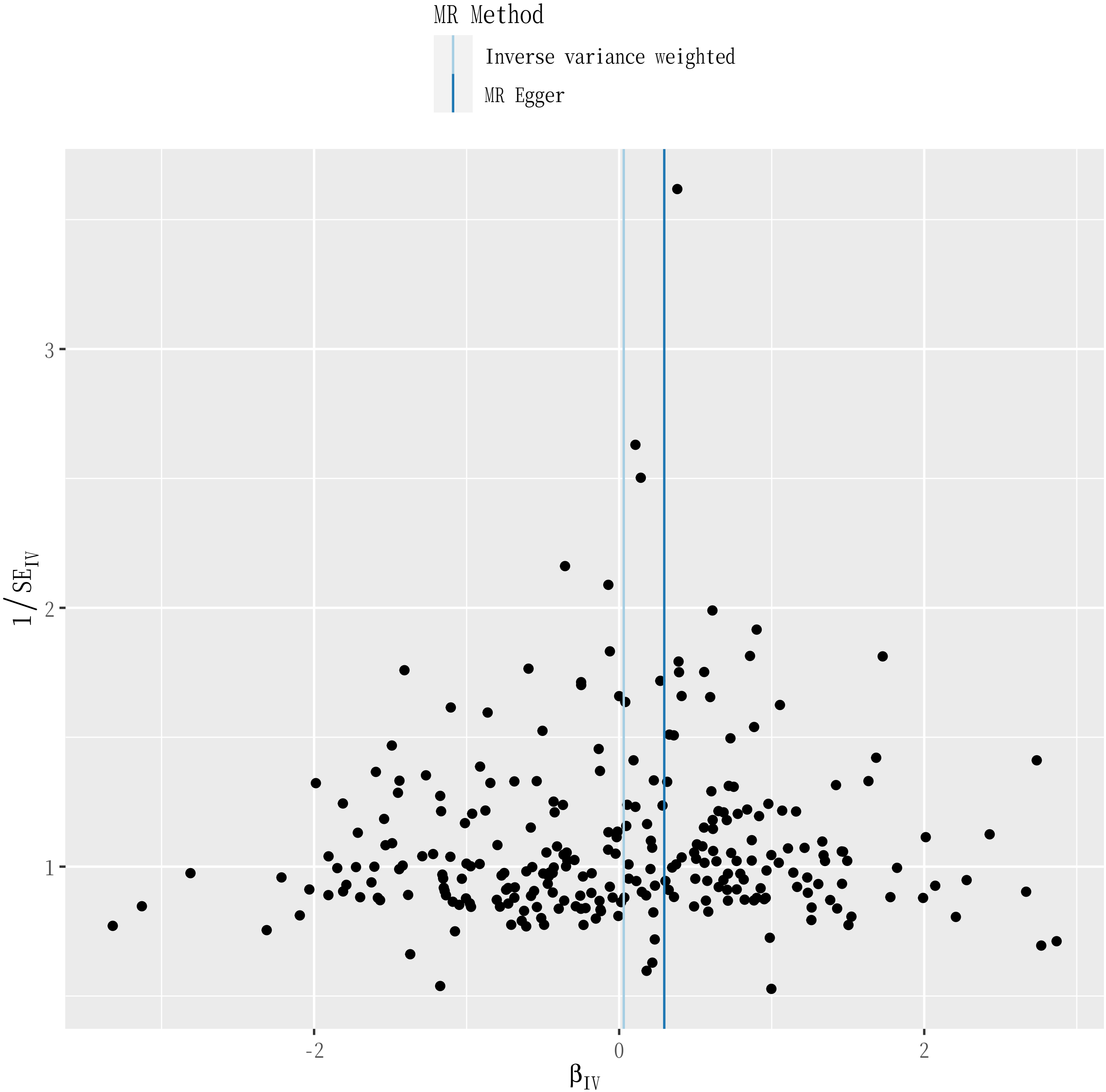
MR Method
Inverse variance weighted
MR Egger
1/SE_IV
β_IV
-2
0
2
1
2
3

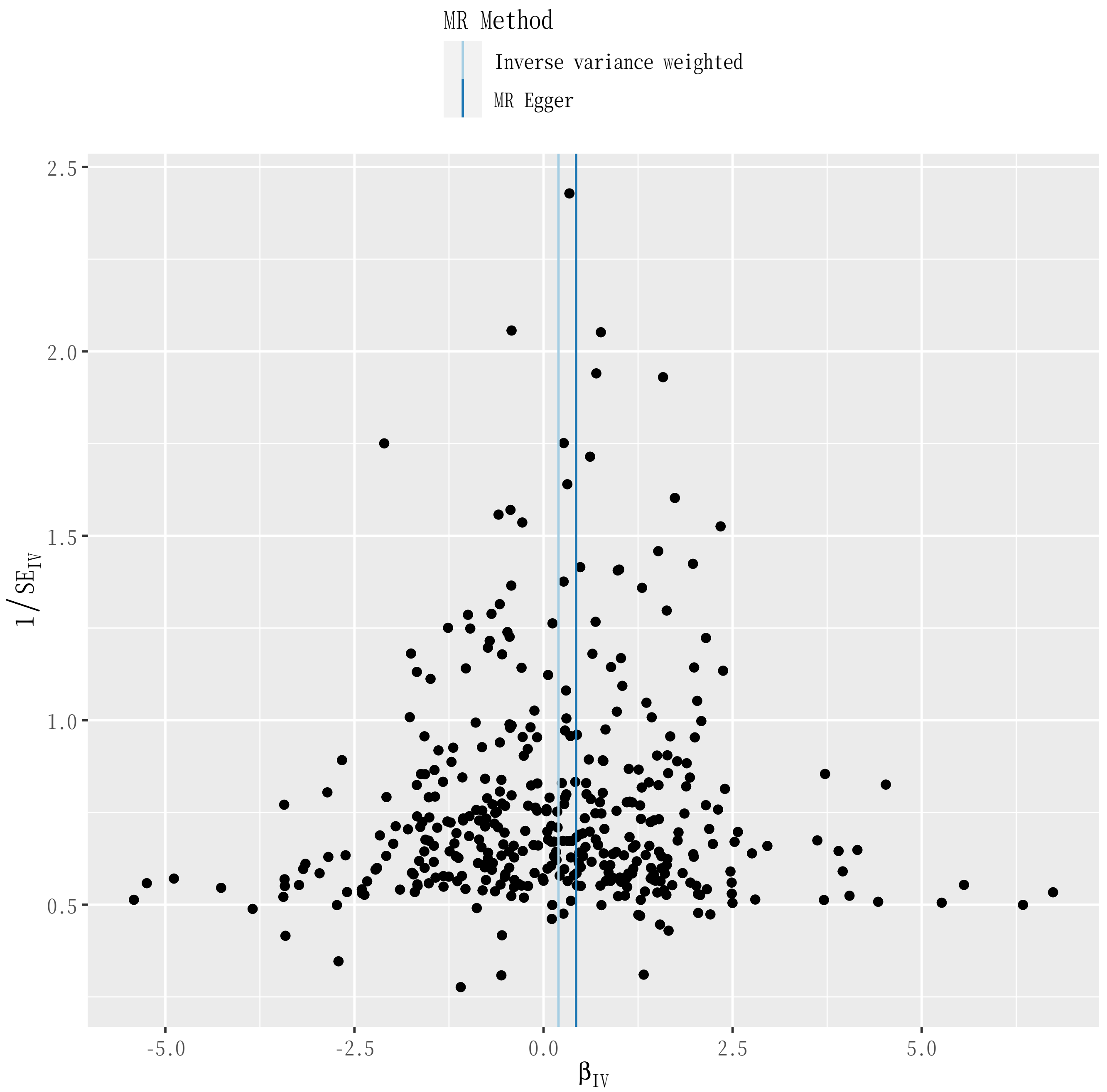

MR Method
Inverse variance weighted
MR Egger
2.5
2.0
1.5
1.0
0.5
$1/SE_{IV}$
-5.0
-2.5
0.0
2.5
5.0
β_{IV}

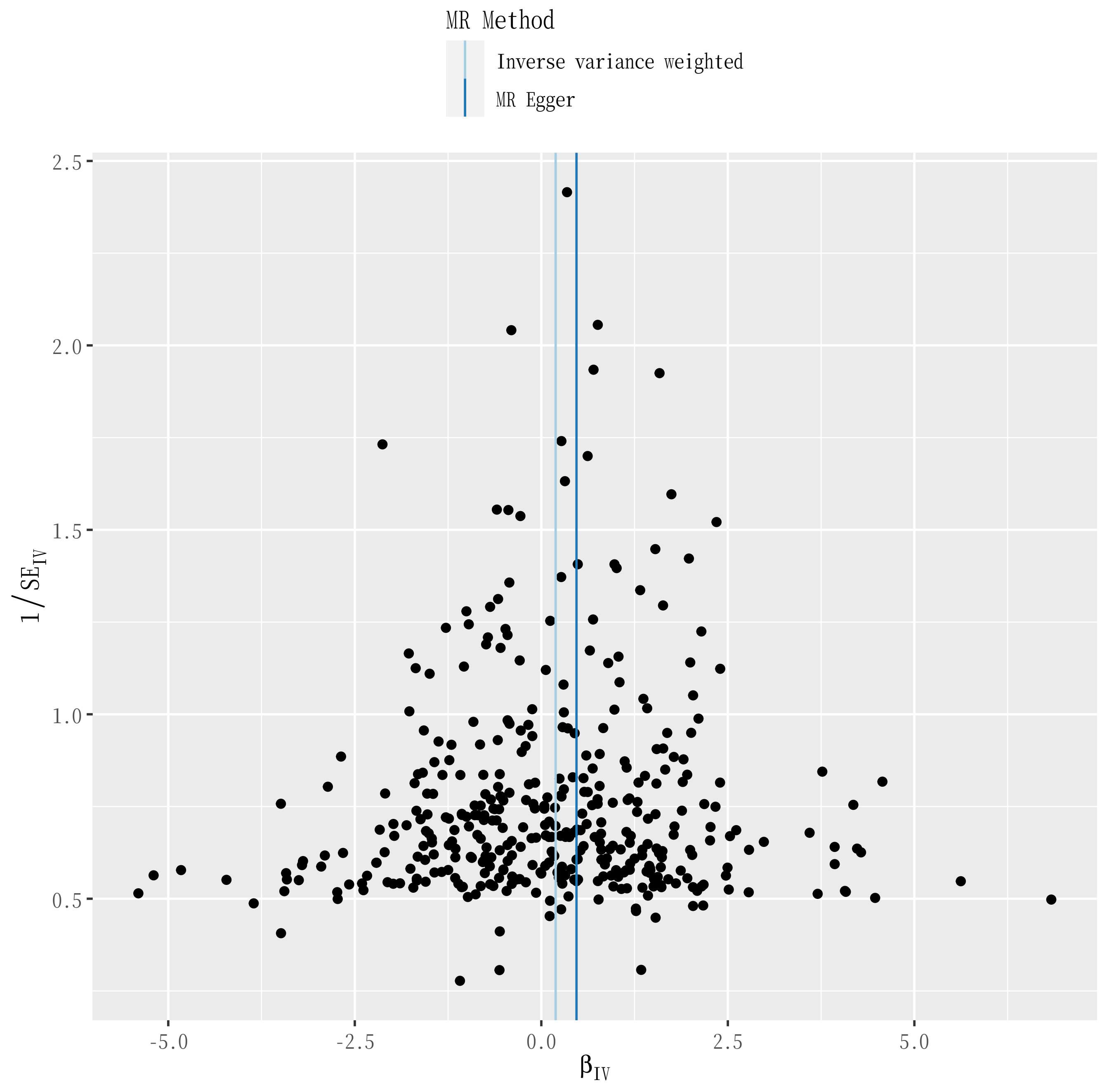

MR Method
Inverse variance weighted
MR Egger
2.5
2.0
1.5
1.0
0.5
$1/SE_{IV}$
-5.0
-2.5
0.0
2.5
5.0
β_{IV}
