# Supplementary material for: The causal relationship between sarcopenic obesity factors and benign prostate hyperplasia
Source: Front Endocrinol (Lausanne). 2023 Nov 8;14:1290639. doi: 10.3389/fendo.2023.1290639 (PMC10663947; doi:10.3389/fendo.2023.1290639)
Supplement: Supplementary file 4 [file DataSheet_5.pdf]

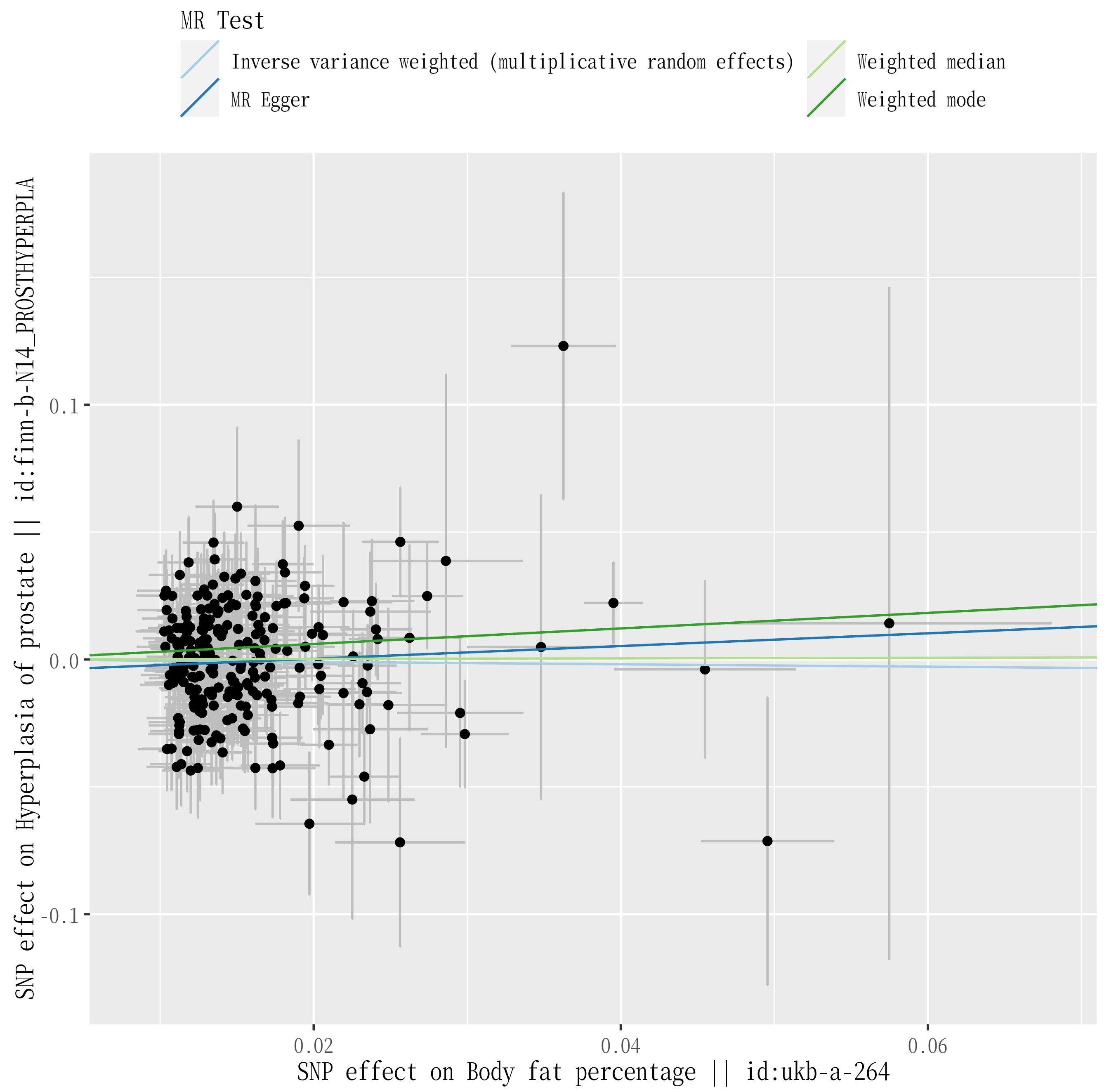
MR Test
Inverse variance weighted (multiplicative random effects)
MR Egger
Weighted median
Weighted mode
SNP effect on Hyperplasia of prostate || id:finn-b-N14_PROSTHYPERPLA
0.1
0.0
-0.1
0.02
0.04
0.06
SNP effect on Body fat percentage || id:ukb-a-264

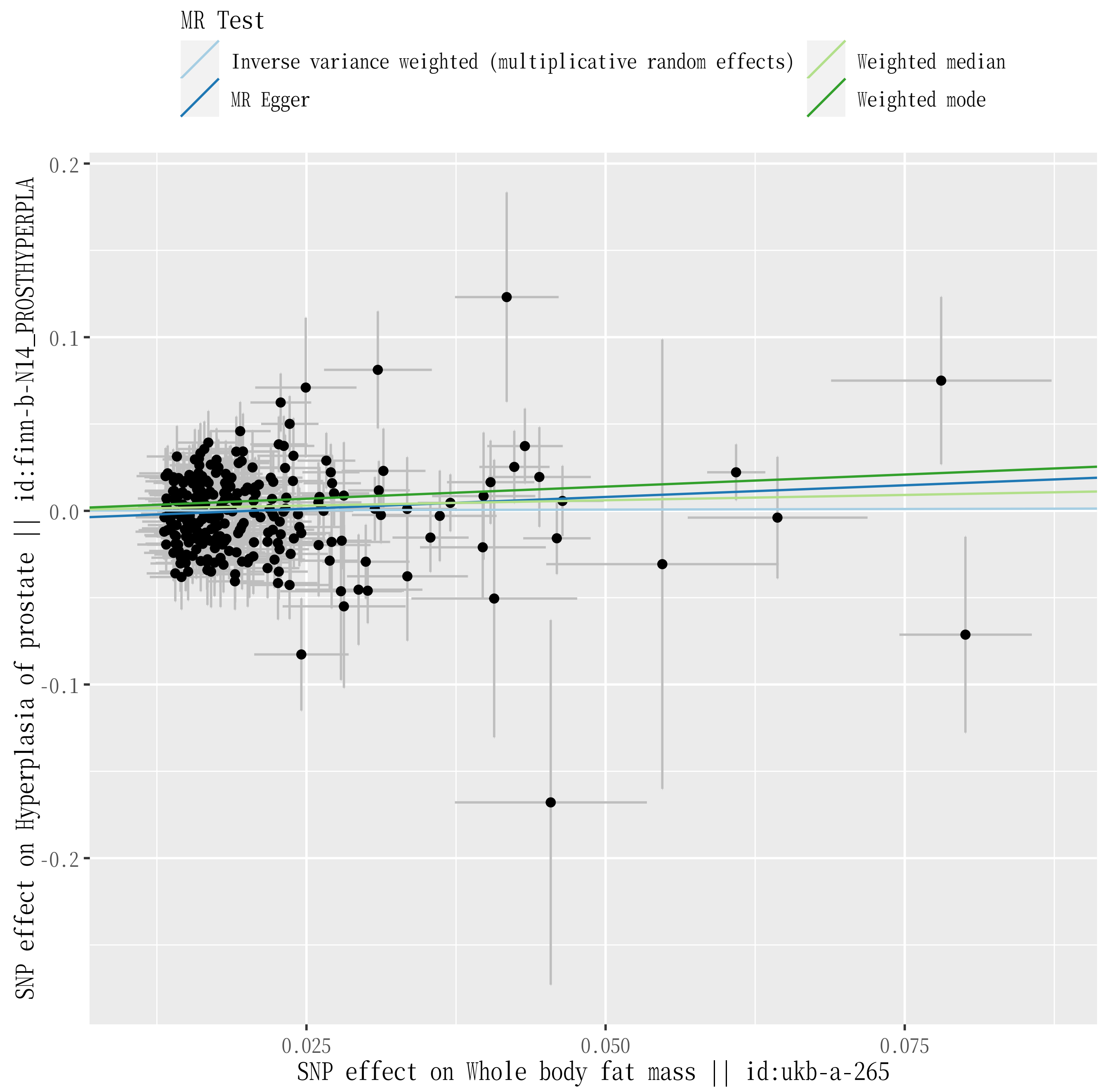
MR Test
Inverse variance weighted (multiplicative random effects)
MR Egger
Weighted median
Weighted mode
0.2
0.1
0.0
-0.1
-0.2
0.025
0.050
0.075
SNP effect on Hyperplasia of prostate || id:finn-b-N14_PROSTHYPERPLA
SNP effect on Whole body fat mass || id:ukb-a-265

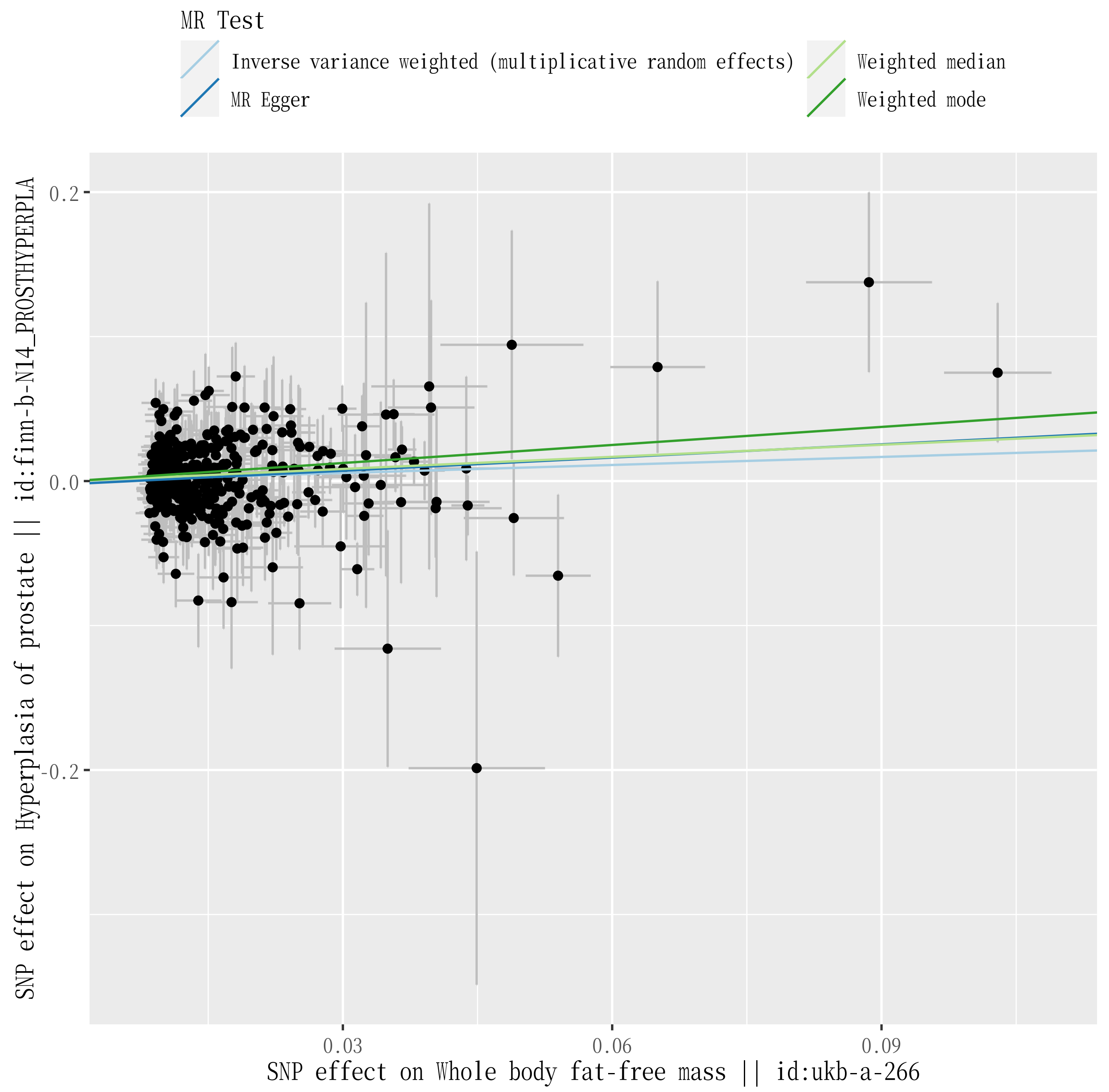
MR Test
Inverse variance weighted (multiplicative random effects)
MR Egger
Weighted median
Weighted mode
0.2
0.0
-0.2
0.03
0.06
0.09
SNP effect on Hyperplasia of prostate || id:finn-b-N14_PROSTHYPERPLA
SNP effect on Whole body fat-free mass || id:ukb-a-266

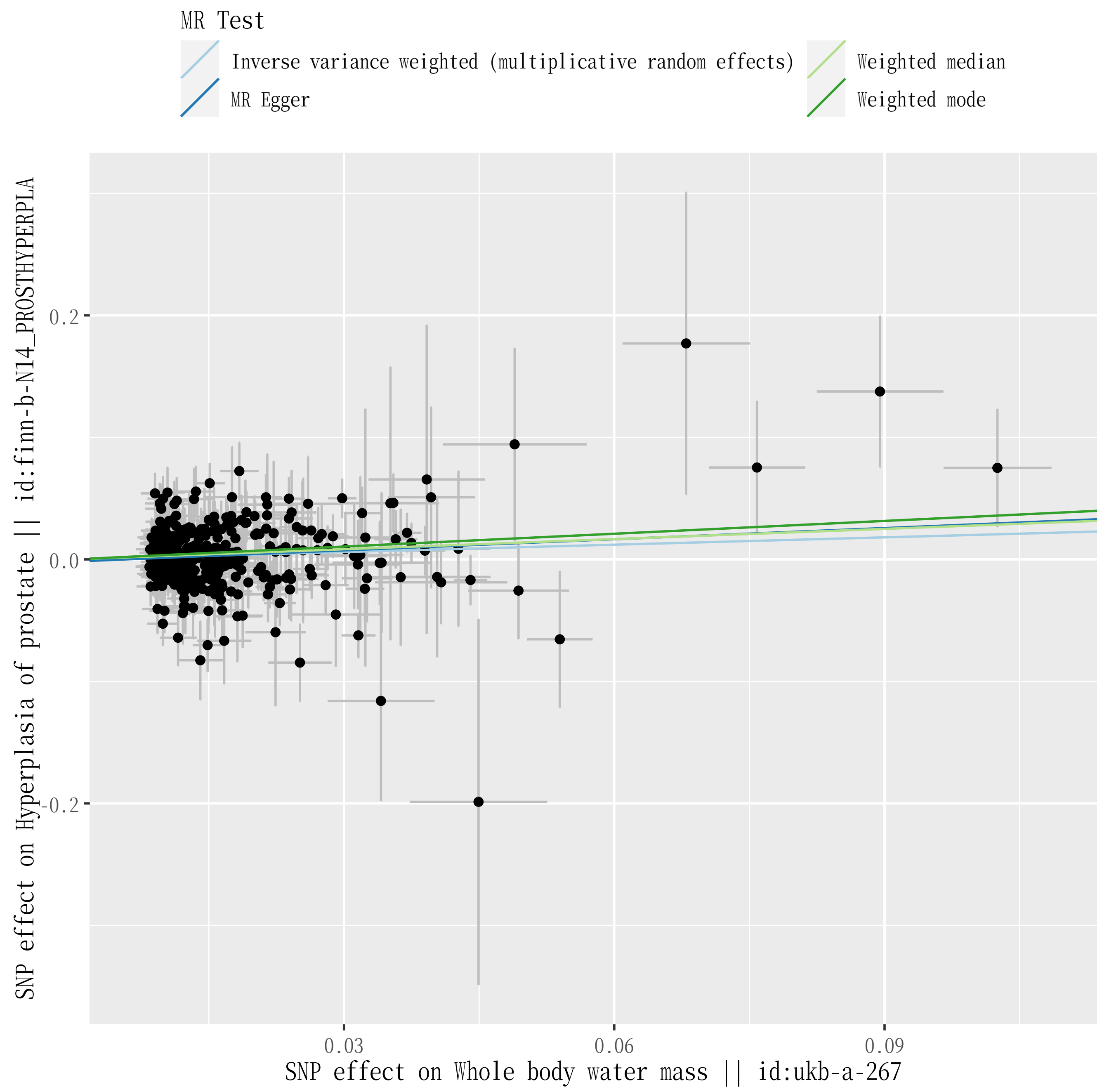

MR Test
Inverse variance weighted (multiplicative random effects)
MR Egger
Weighted median
Weighted mode
0.2
0.0
-0.2
0.03
0.06
0.09
SNP effect on Whole body water mass || id:ukb-a-267
SNP effect on Hyperplasia of prostate || id:finn-b-N14_PROSTHYPERPLA

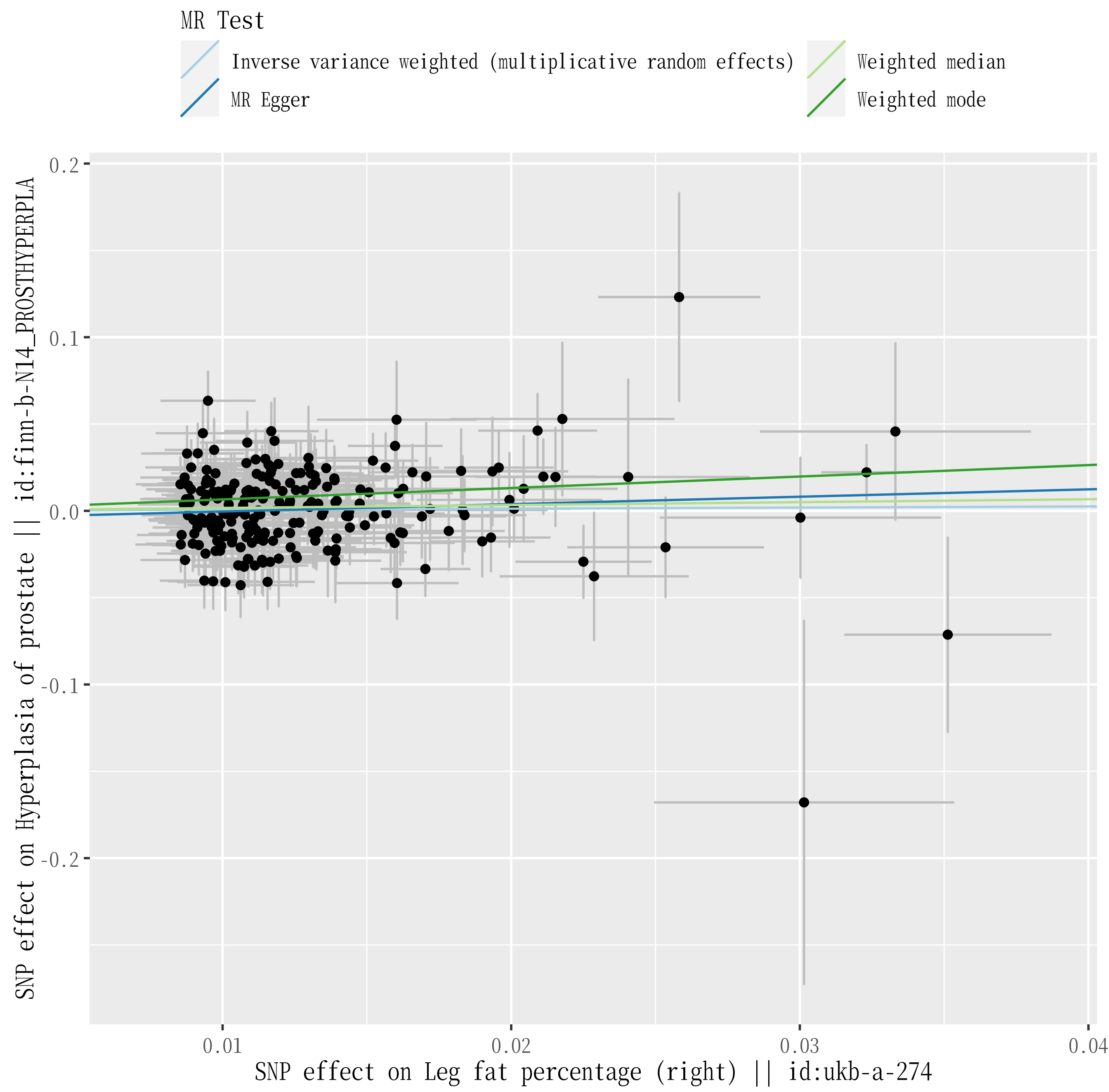
MR Test
Inverse variance weighted (multiplicative random effects)
MR Egger
Weighted median
Weighted mode
0.2
0.1
0.0
-0.1
-0.2
SNP effect on Hyperplasia of prostate || id:finn-b-N14_PROSTHYPERPLA
0.01
0.02
0.03
0.04
SNP effect on Leg fat percentage (right) || id:ukb-a-274

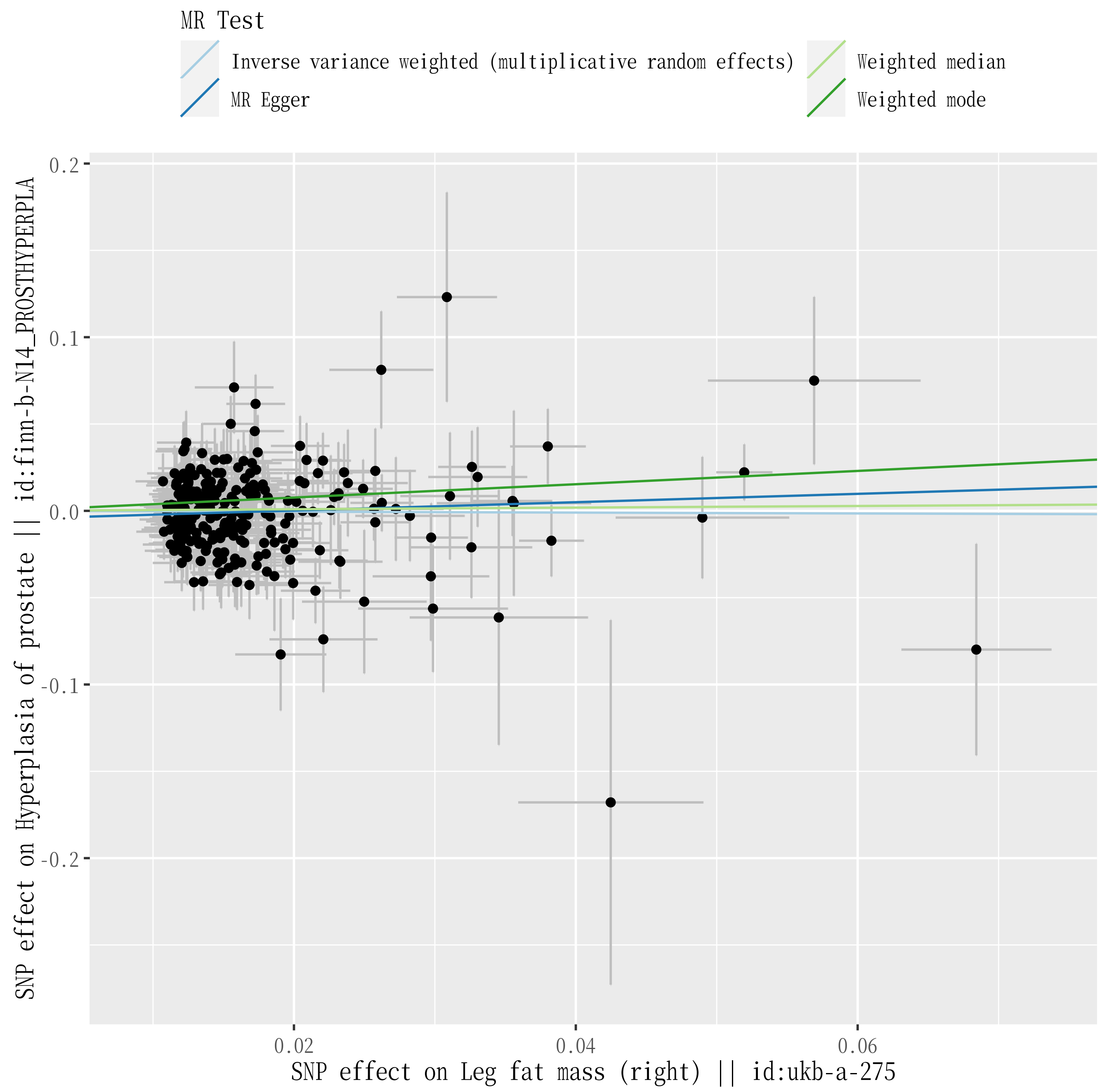

MR Test
Inverse variance weighted (multiplicative random effects)
MR Egger
Weighted median
Weighted mode
SNP effect on Hyperplasia of prostate || id:finn-b-N14_PROSTHYPERPLA
0.2
0.1
0.0
-0.1
-0.2
0.02
0.04
0.06
SNP effect on Leg fat mass (right) || id:ukb-a-275

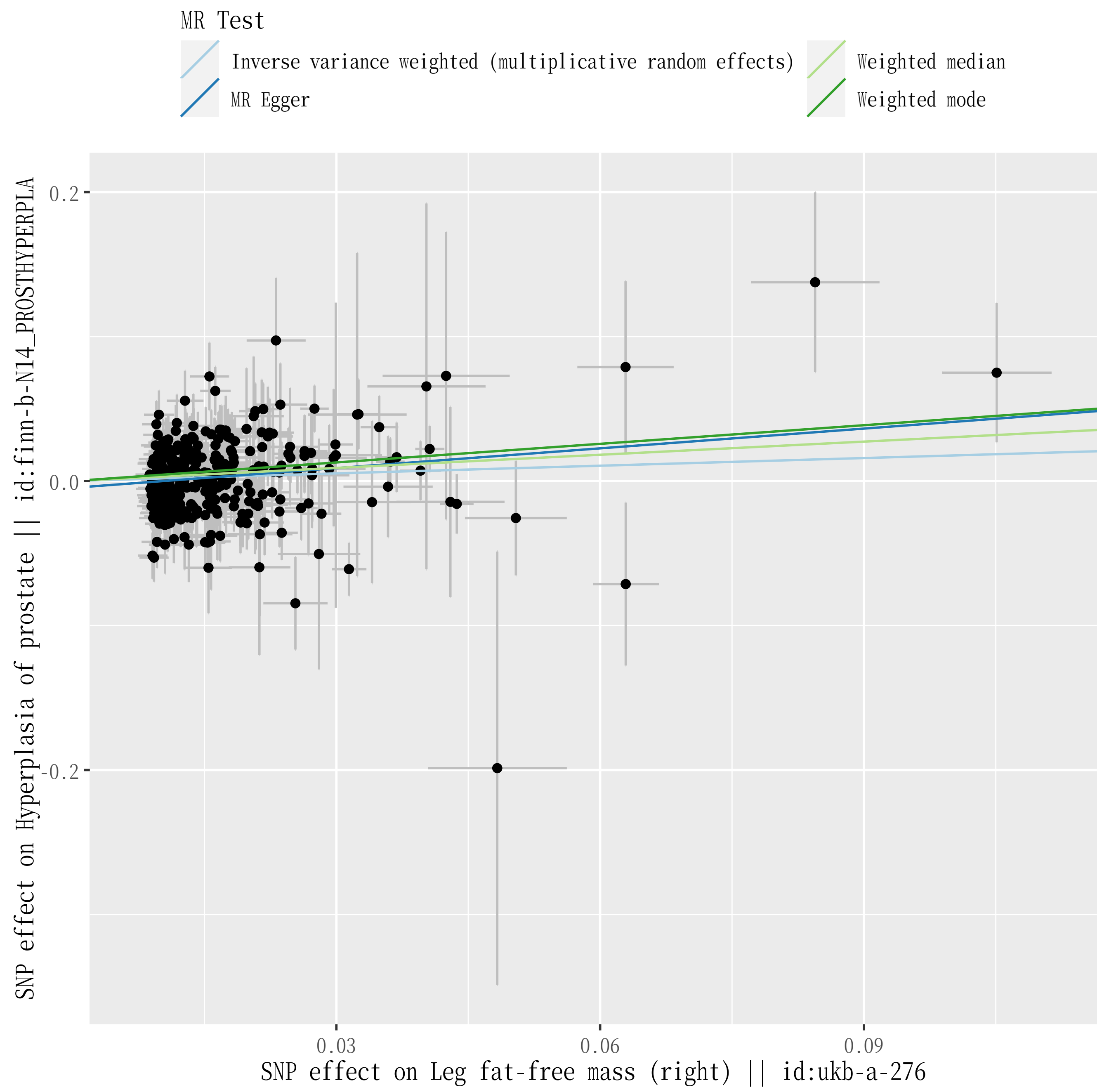
MR Test
Inverse variance weighted (multiplicative random effects)
MR Egger
Weighted median
Weighted mode
SNP effect on Hyperplasia of prostate || id:finn-b-N14_PROSTHYPERPLA
0.2
0.0
-0.2
0.03
0.06
0.09
SNP effect on Leg fat-free mass (right) || id:ukb-a-276

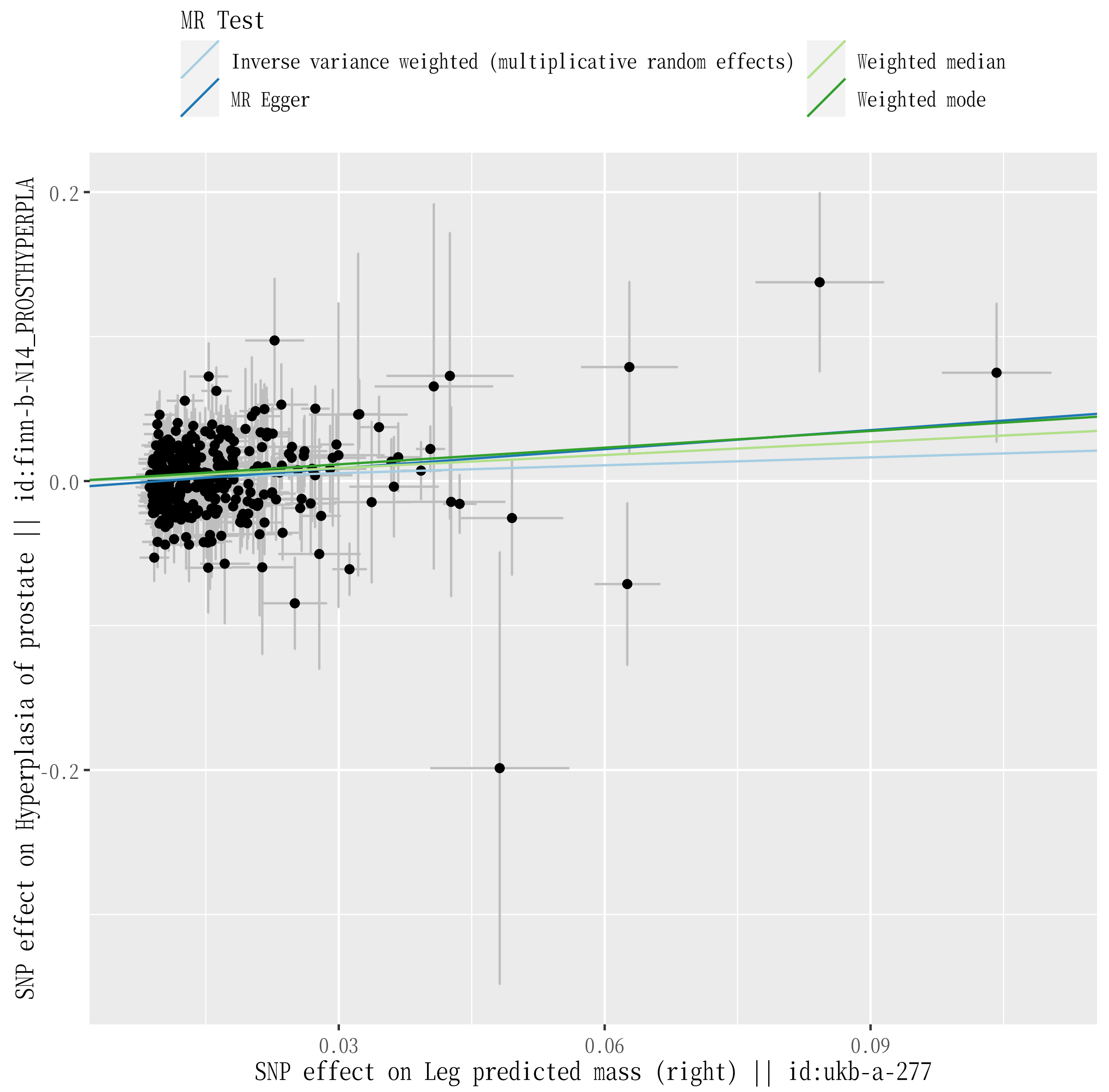

MR Test
Inverse variance weighted (multiplicative random effects)
MR Egger
Weighted median
Weighted mode
0.2
0.0
-0.2
0.03
0.06
0.09
SNP effect on Hyperplasia of prostate || id:finn-b-N14_PROSTHYPERPLA
SNP effect on Leg predicted mass (right) || id:ukb-a-277

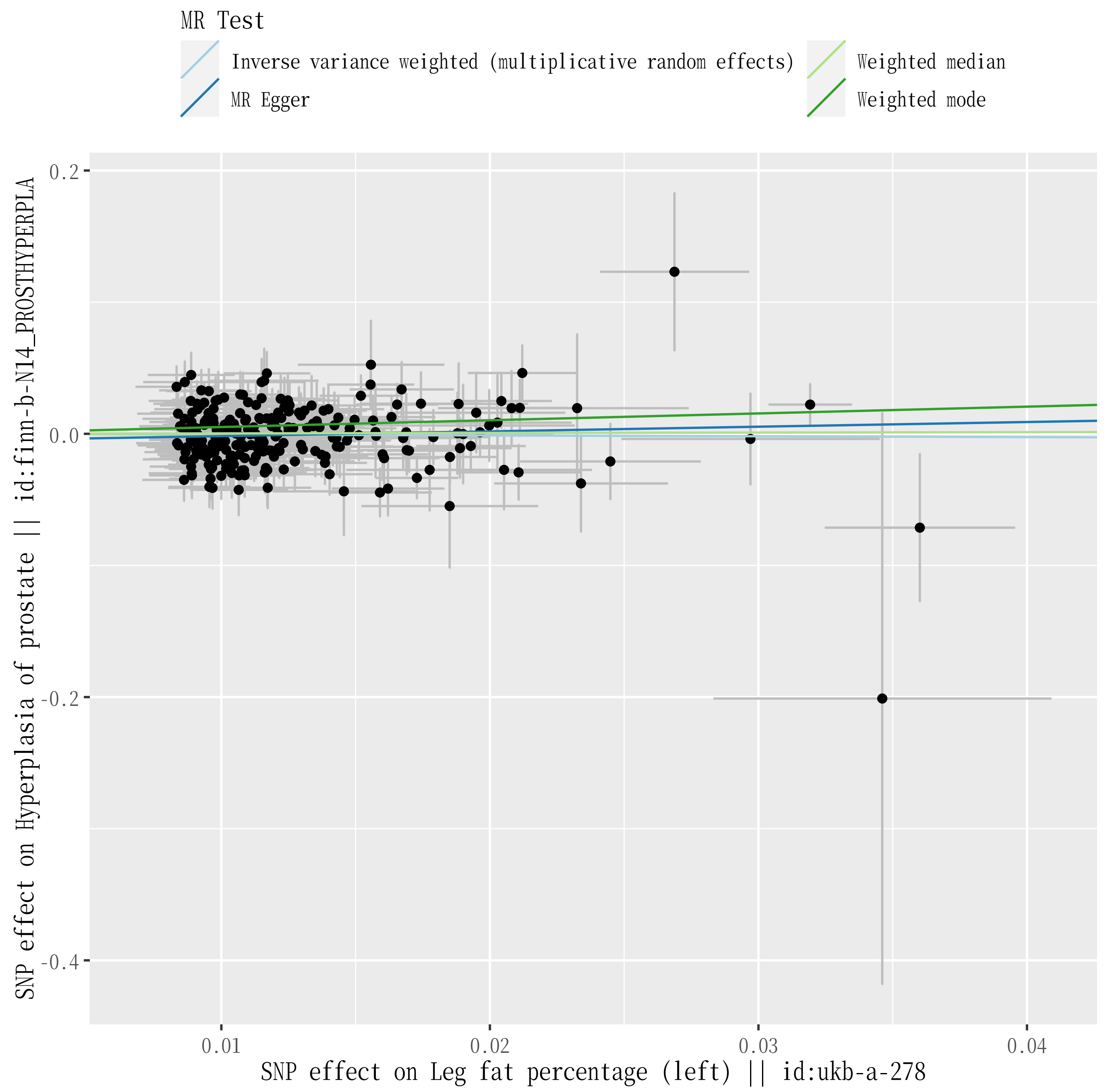
MR Test
Inverse variance weighted (multiplicative random effects)
MR Egger
Weighted median
Weighted mode
0.2
0.0
-0.2
-0.4
0.01
0.02
0.03
0.04
SNP effect on Hyperplasia of prostate || id:finn-b-N14_PROSTHYPERPLA
SNP effect on Leg fat percentage (left) || id:ukb-a-278

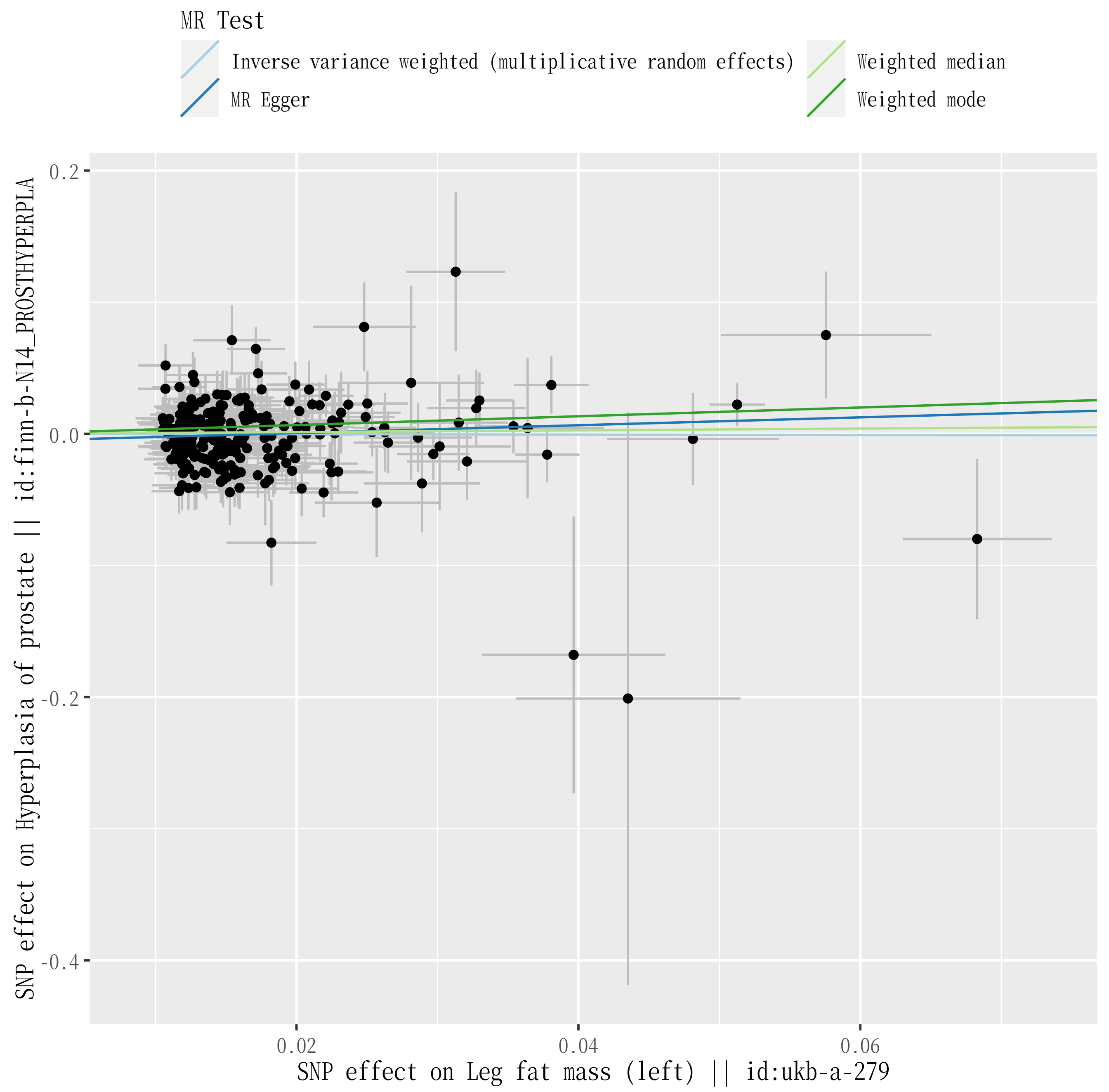

MR Test
Inverse variance weighted (multiplicative random effects)
MR Egger
Weighted median
Weighted mode
0.2
0.0
-0.2
-0.4
0.02
0.04
0.06
SNP effect on Leg fat mass (left) || id:ukb-a-279
SNP effect on Hyperplasia of prostate || id:finn-b-N14_PROSTHYPERPLA

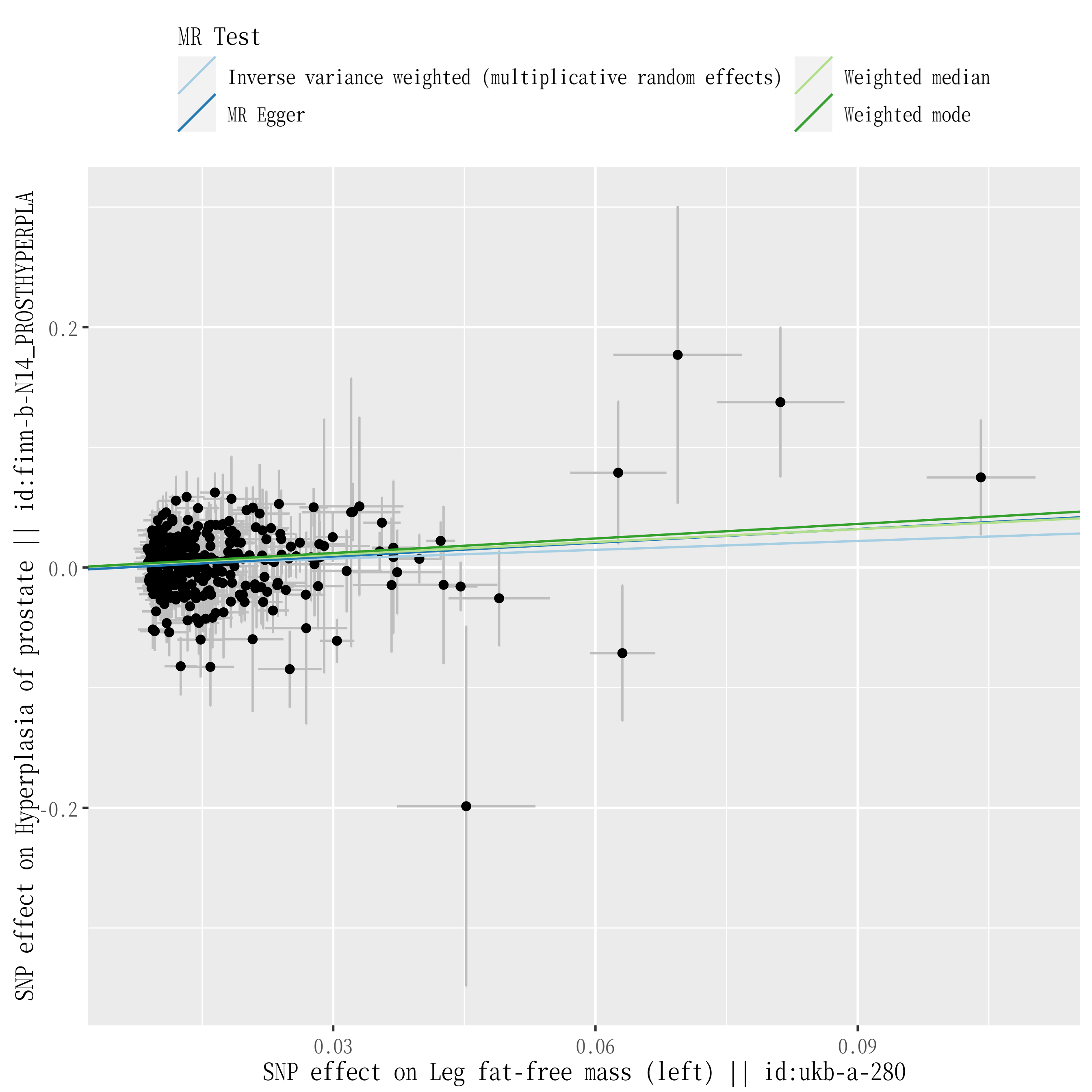
MR Test
Inverse variance weighted (multiplicative random effects)
MR Egger
Weighted median
Weighted mode
0.2
0.0
-0.2
0.03
0.06
0.09
SNP effect on Hyperplasia of prostate || id:finn-b-N14_PROSTHYPERPLA
SNP effect on Leg fat-free mass (left) || id:ukb-a-280

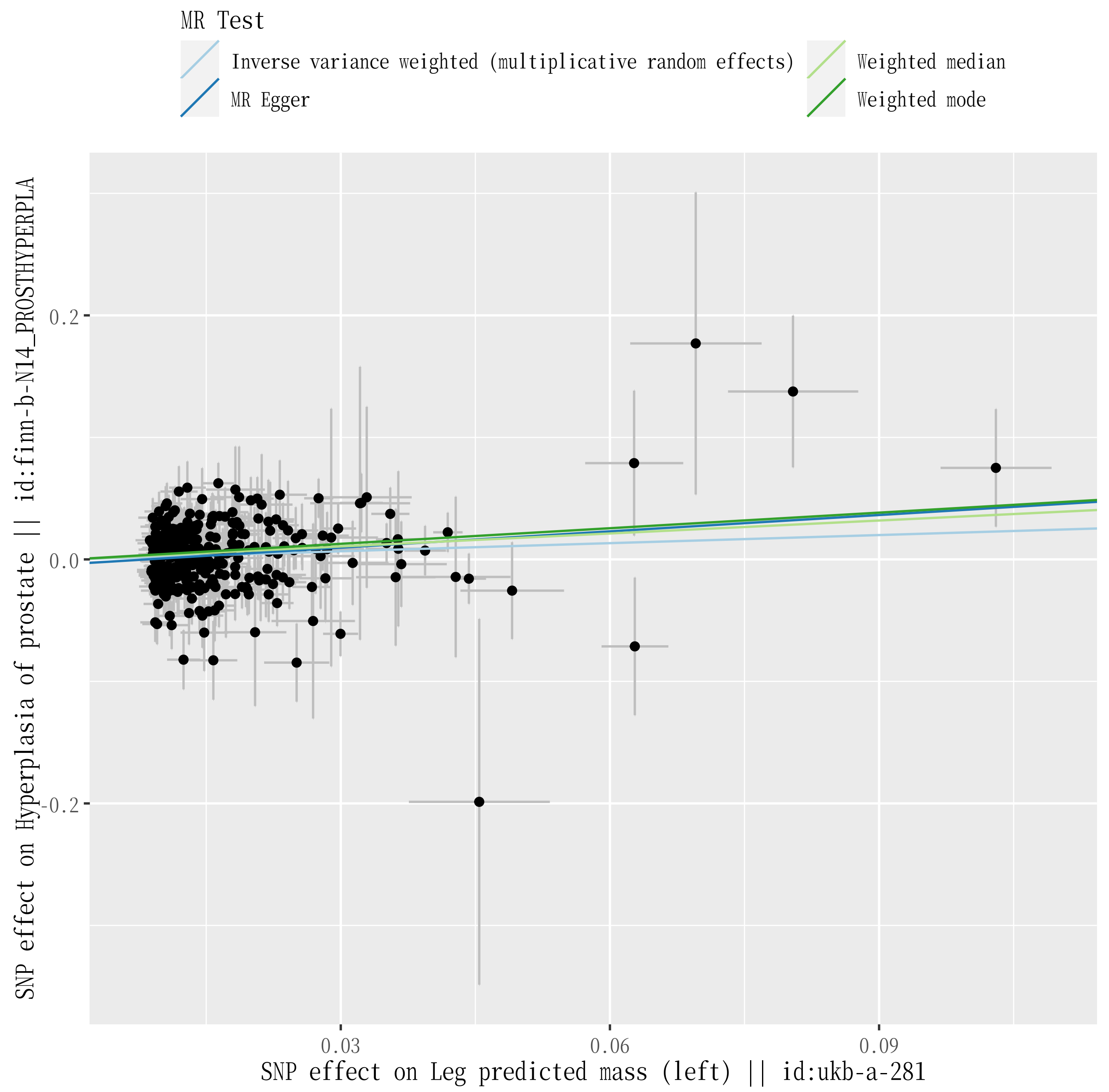

MR Test
Inverse variance weighted (multiplicative random effects)
MR Egger
Weighted median
Weighted mode
SNP effect on Hyperplasia of prostate || id:finn-b-N14_PROSTHYPERPLA
0.2
0.0
-0.2
0.03
0.06
0.09
SNP effect on Leg predicted mass (left) || id:ukb-a-281

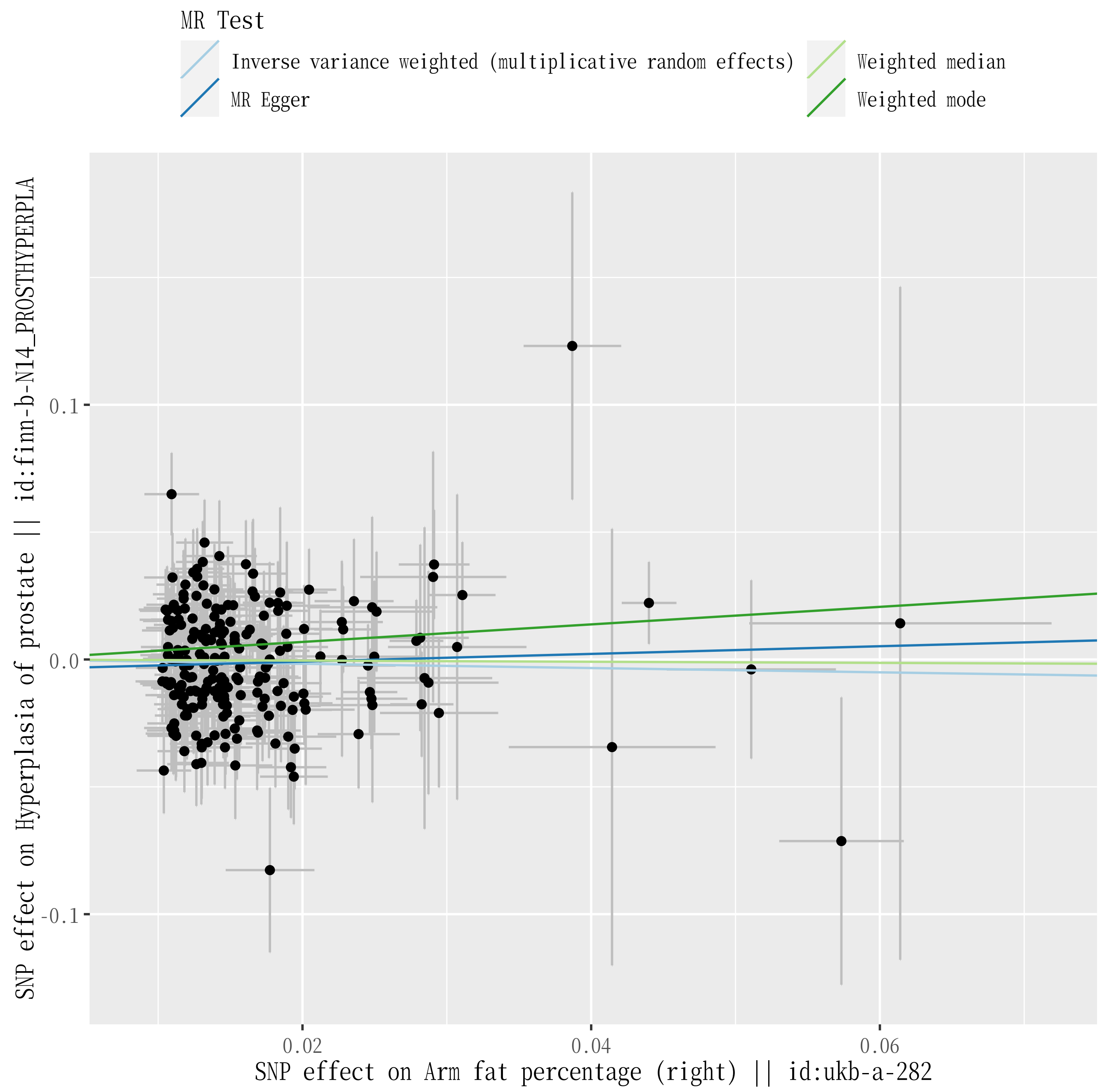

MR Test
Inverse variance weighted (multiplicative random effects)
MR Egger
Weighted median
Weighted mode
0.1
0.0
-0.1
0.02
0.04
0.06
SNP effect on Hyperplasia of prostate || id:finn-b-N14_PROSTHYPERPLA
SNP effect on Arm fat percentage (right) || id:ukb-a-282

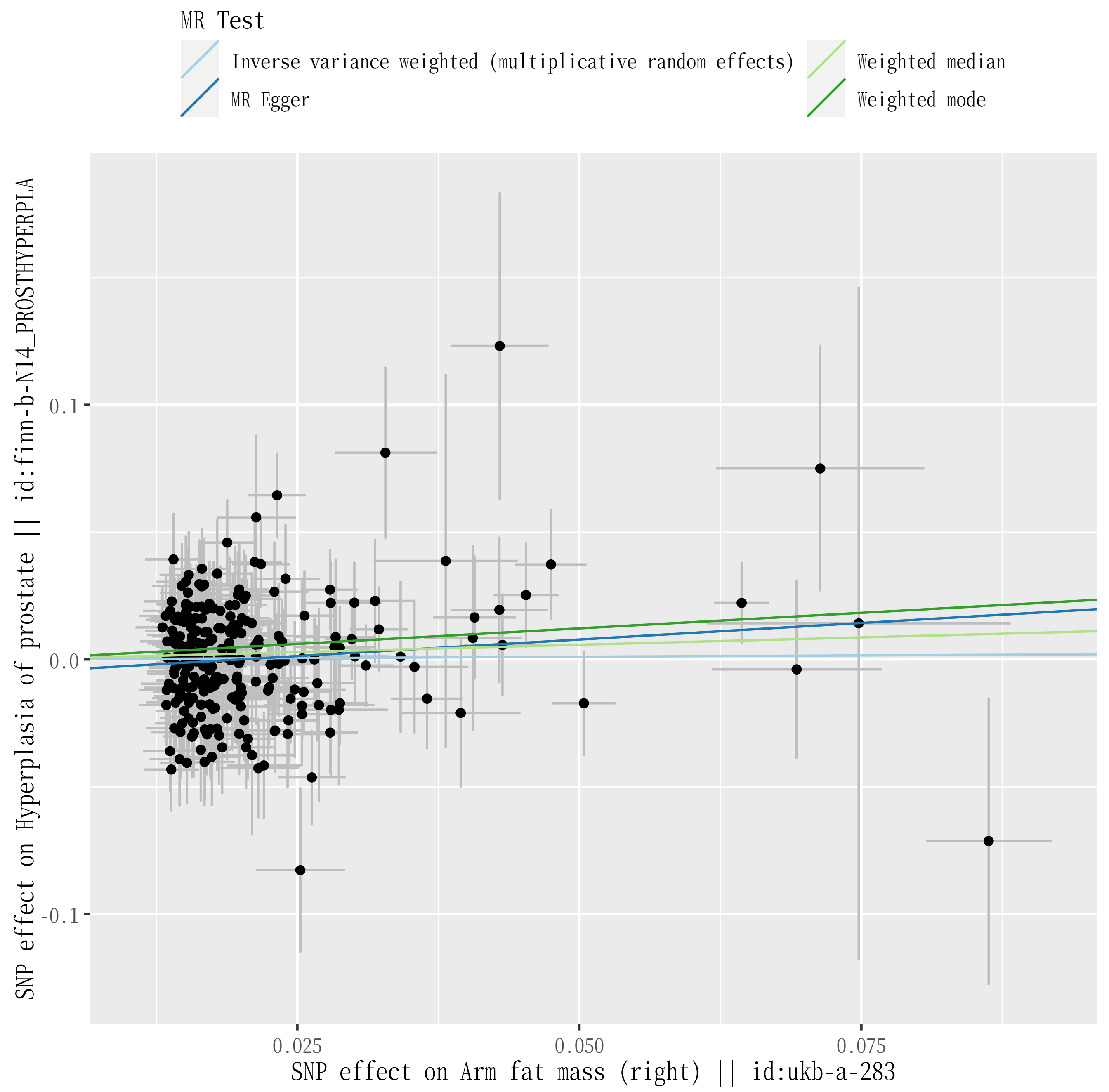
MR Test
Inverse variance weighted (multiplicative random effects)
MR Egger
Weighted median
Weighted mode
SNP effect on Hyperplasia of prostate || id:finn-b-N14_PROSTHYPERPLA
0.1
0.0
-0.1
0.025
0.050
0.075
SNP effect on Arm fat mass (right) || id:ukb-a-283

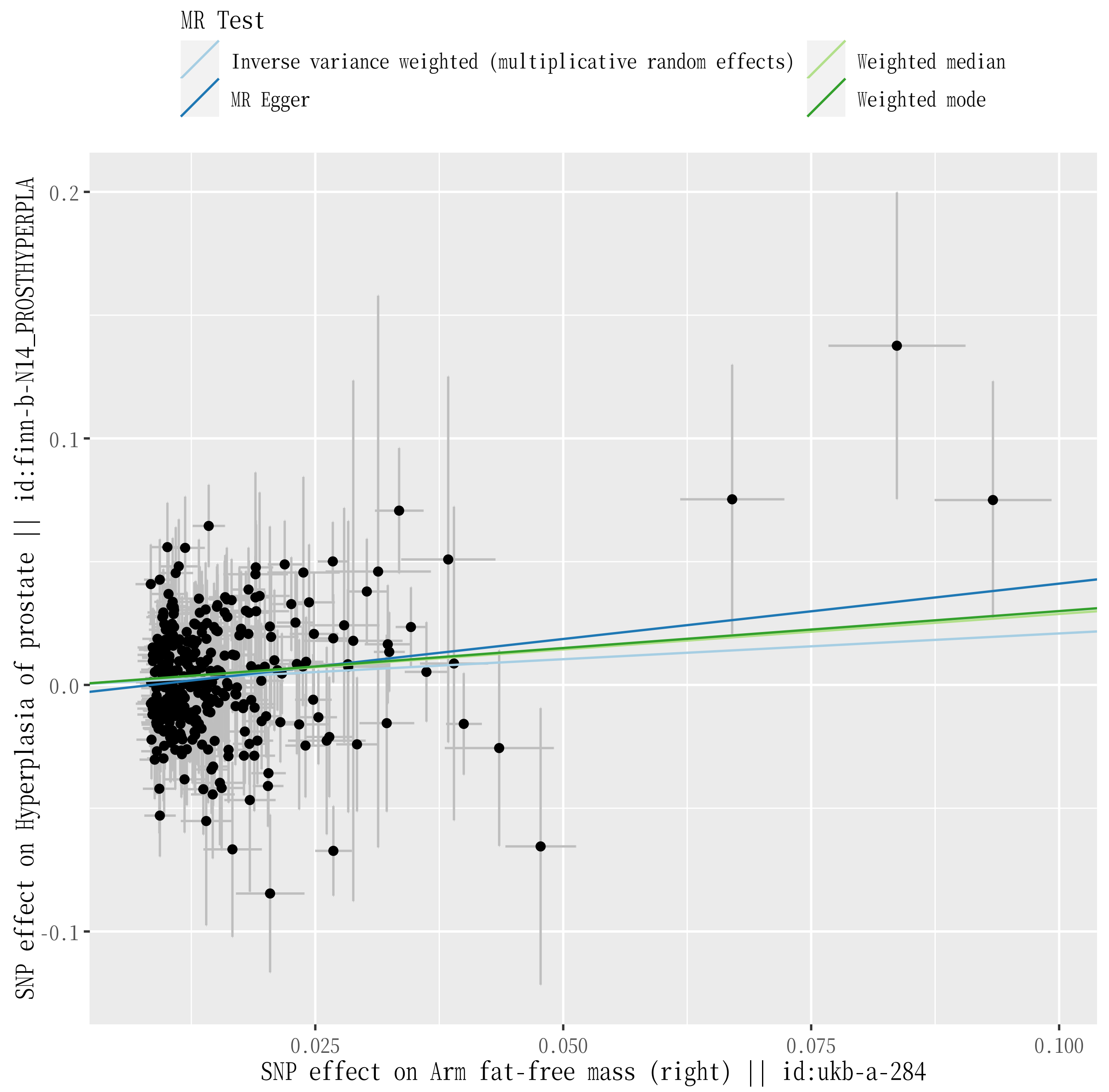
MR Test
Inverse variance weighted (multiplicative random effects)
MR Egger
Weighted median
Weighted mode
0.2
0.1
0.0
-0.1
SNP effect on Hyperplasia of prostate || id:finn-b-N14_PROSTHYPERPLA
0.025
0.050
0.075
0.100
SNP effect on Arm fat-free mass (right) || id:ukb-a-284

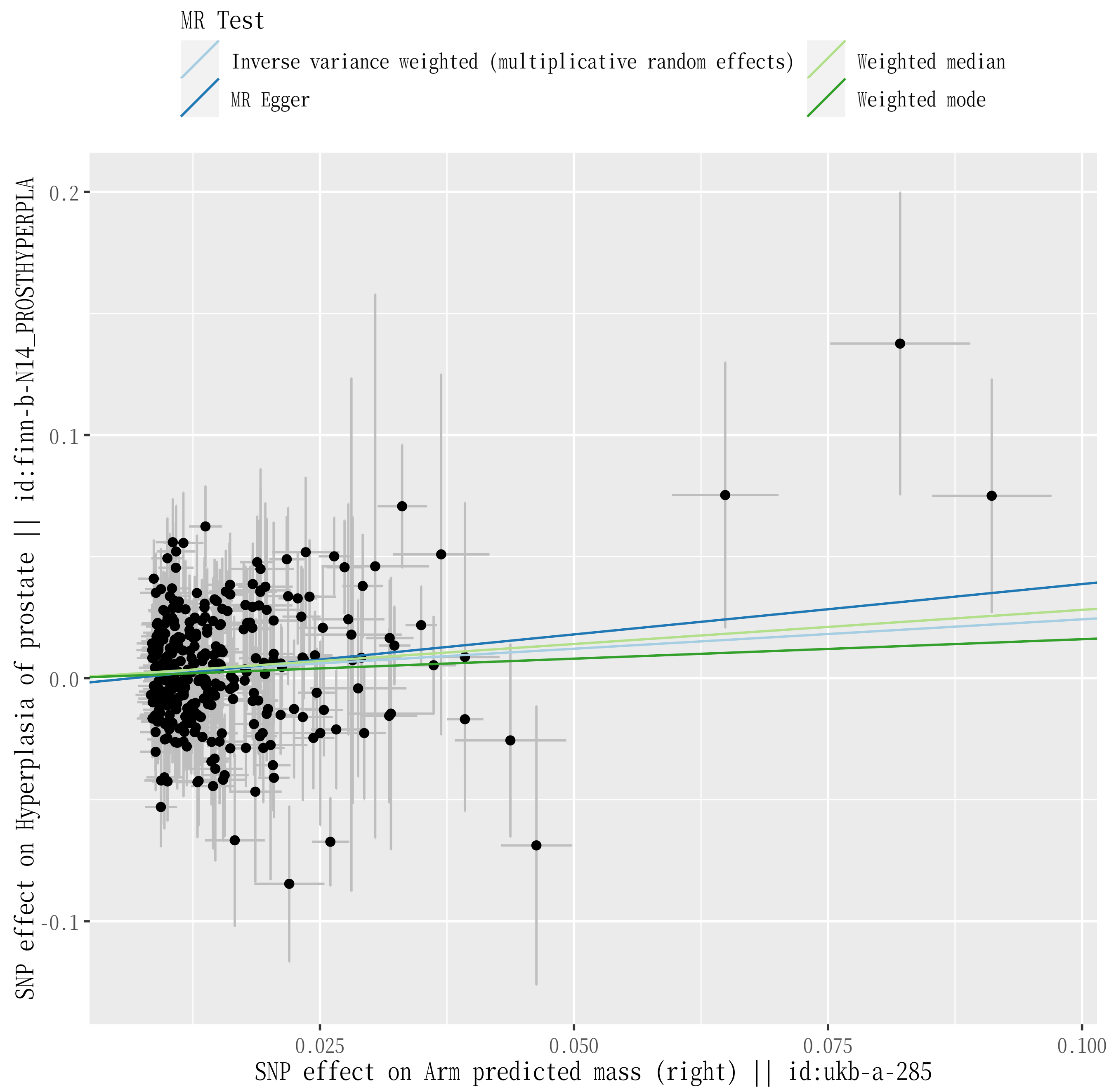
MR Test
Inverse variance weighted (multiplicative random effects)
MR Egger
Weighted median
Weighted mode
SNP effect on Hyperplasia of prostate || id:finn-b-N14_PROSTHYPERPLA
0.2
0.1
0.0
-0.1
0.025
0.050
0.075
0.100
SNP effect on Arm predicted mass (right) || id:ukb-a-285

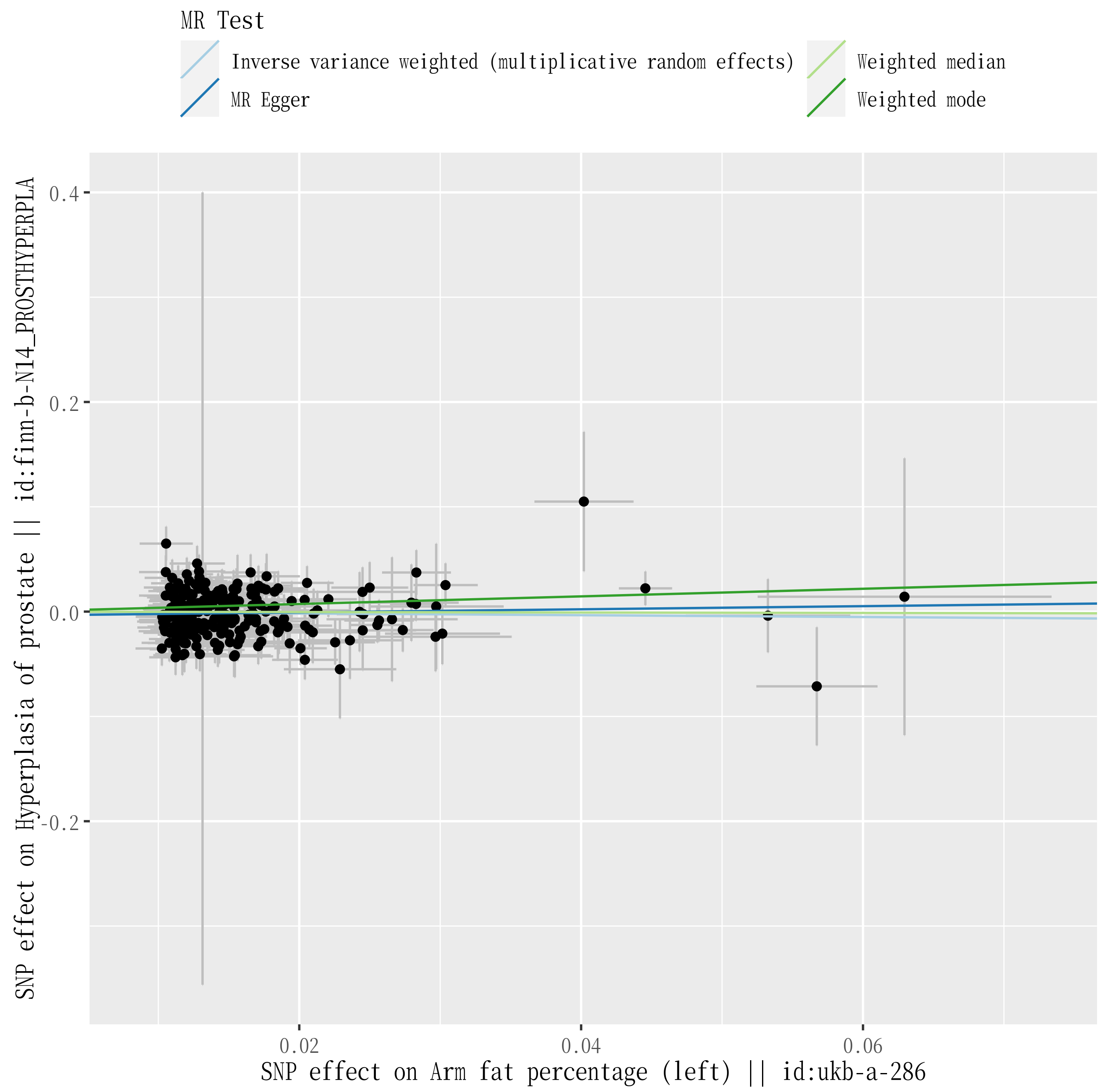

MR Test
Inverse variance weighted (multiplicative random effects)
MR Egger
Weighted median
Weighted mode
0.4
0.2
0.0
-0.2
0.02
0.04
0.06
SNP effect on Hyperplasia of prostate || id:finn-b-N14_PROSTHYPERPLA
SNP effect on Arm fat percentage (left) || id:ukb-a-286

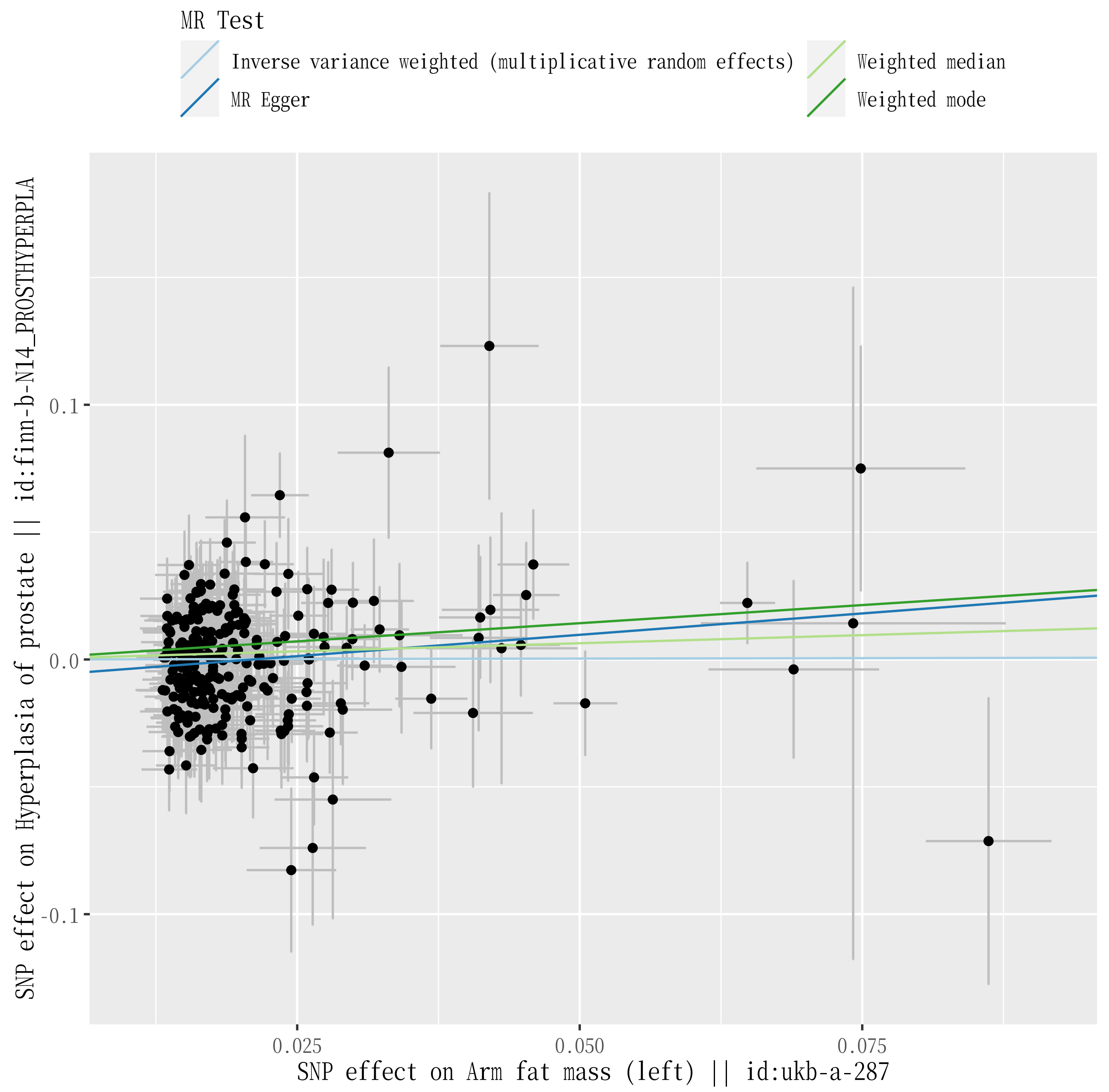

MR Test
Inverse variance weighted (multiplicative random effects)
MR Egger
Weighted median
Weighted mode
0.1
0.0
-0.1
0.025
0.050
0.075
SNP effect on Arm fat mass (left) || id:ukb-a-287
SNP effect on Hyperplasia of prostate || id:finn-b-N14_PROSTHYPERPLA

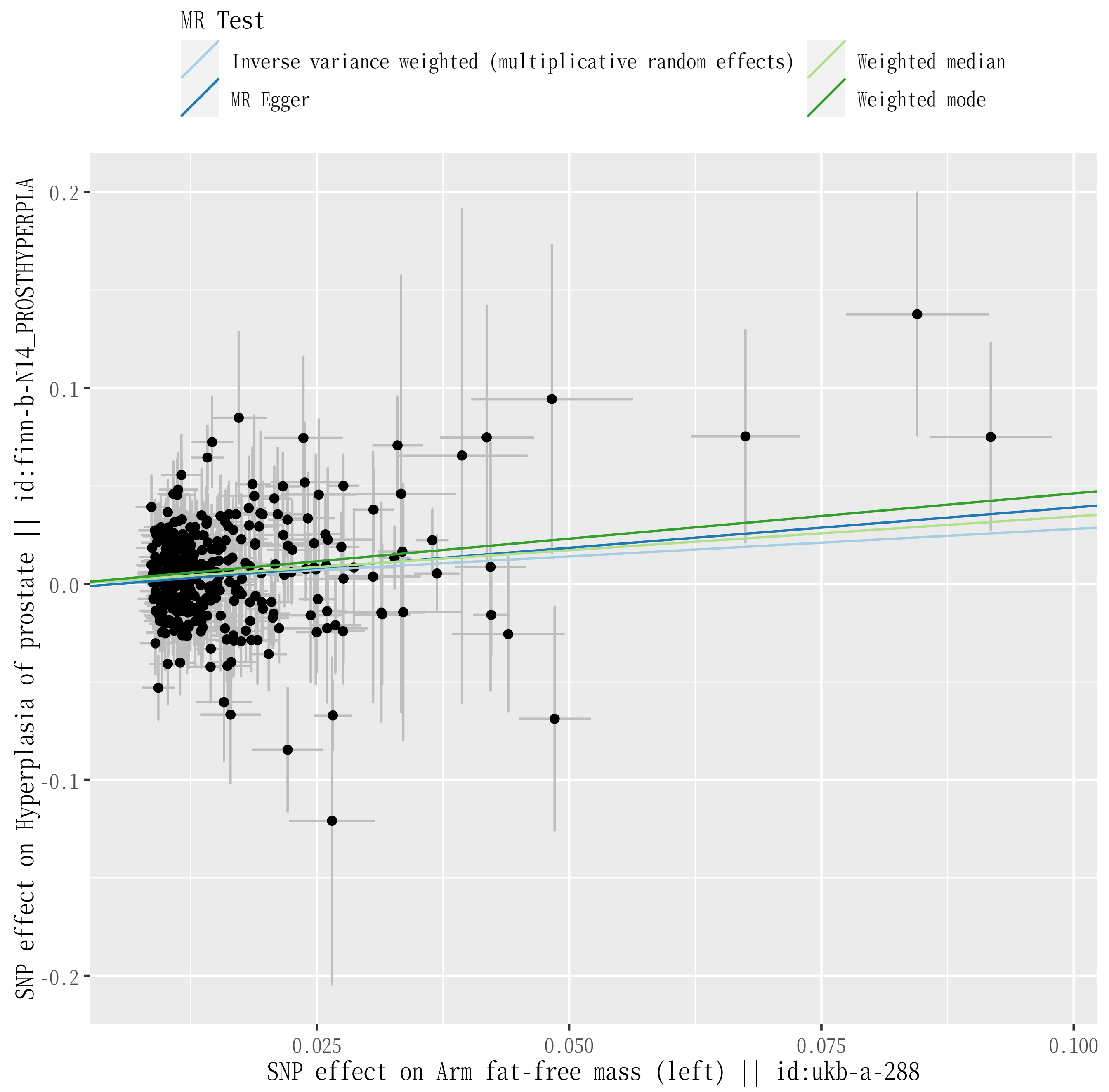
MR Test
Inverse variance weighted (multiplicative random effects)
Weighted median
MR Egger
Weighted mode
SNP effect on Hyperplasia of prostate || id:finn-b-N14_PROSTHYPERPLA
0.2
0.1
0.0
-0.1
-0.2
0.025
0.050
0.075
0.100
SNP effect on Arm fat-free mass (left) || id:ukb-a-288

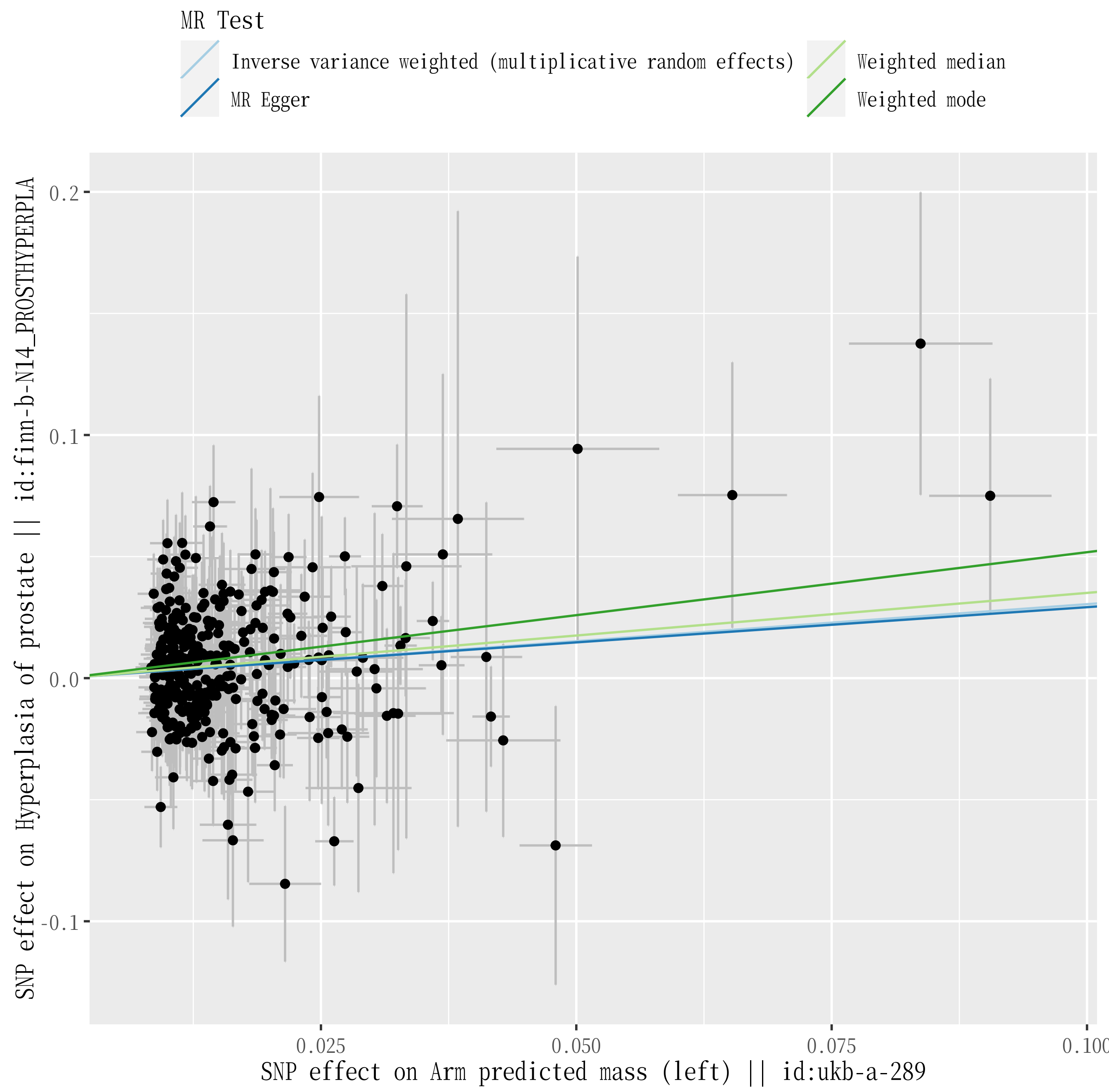
MR Test
Inverse variance weighted (multiplicative random effects)
MR Egger
Weighted median
Weighted mode
0.2
0.1
0.0
-0.1
SNP effect on Hyperplasia of prostate || id:finn-b-N14_PROSTHYPERPLA
0.025
0.050
0.075
0.100
SNP effect on Arm predicted mass (left) || id:ukb-a-289

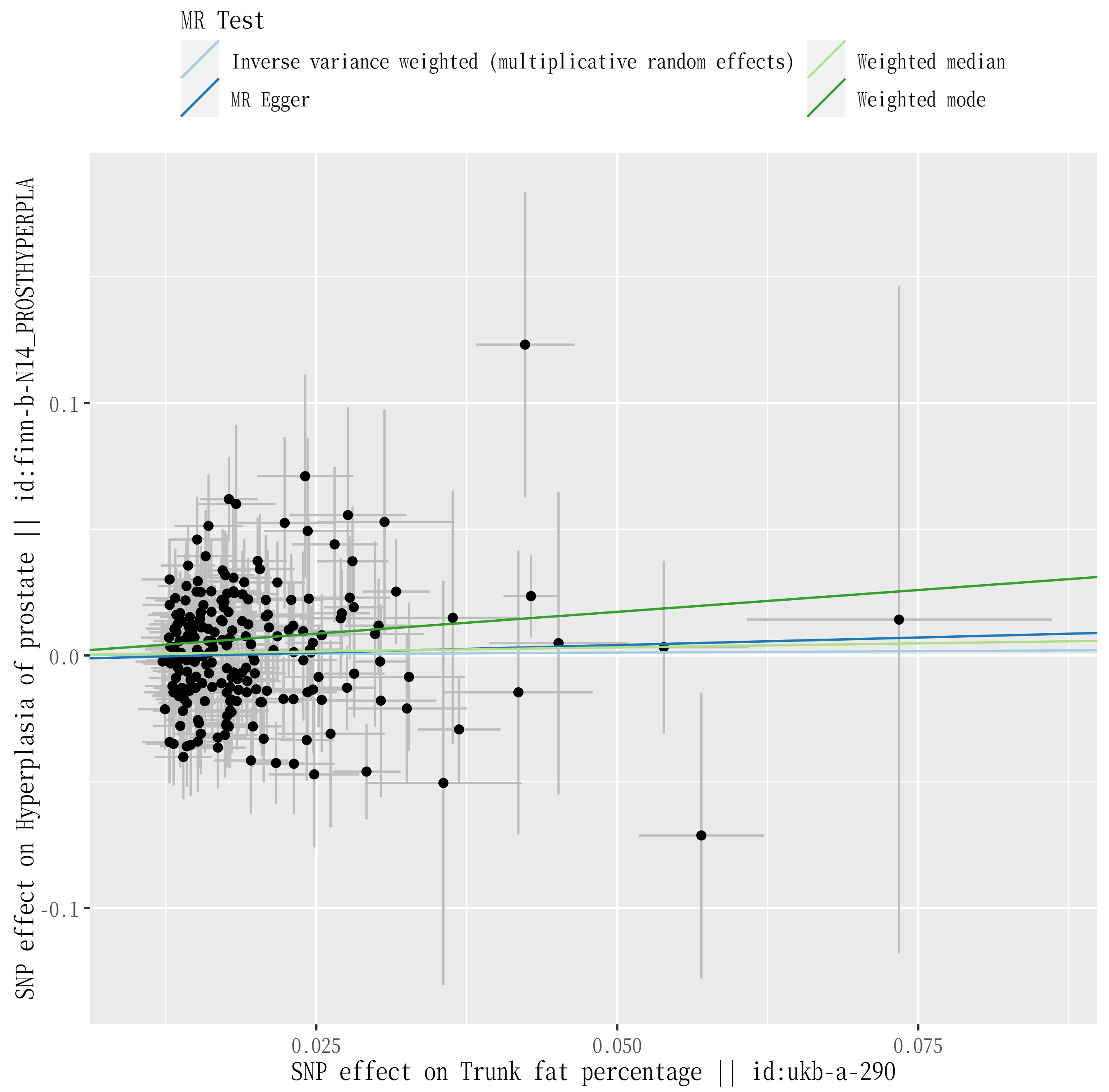

MR Test
Inverse variance weighted (multiplicative random effects)
MR Egger
Weighted median
Weighted mode
SNP effect on Hyperplasia of prostate || id:finn-b-N14_PROSTHYPERPLA
0.1
0.0
-0.1
0.025
0.050
0.075
SNP effect on Trunk fat percentage || id:ukb-a-290

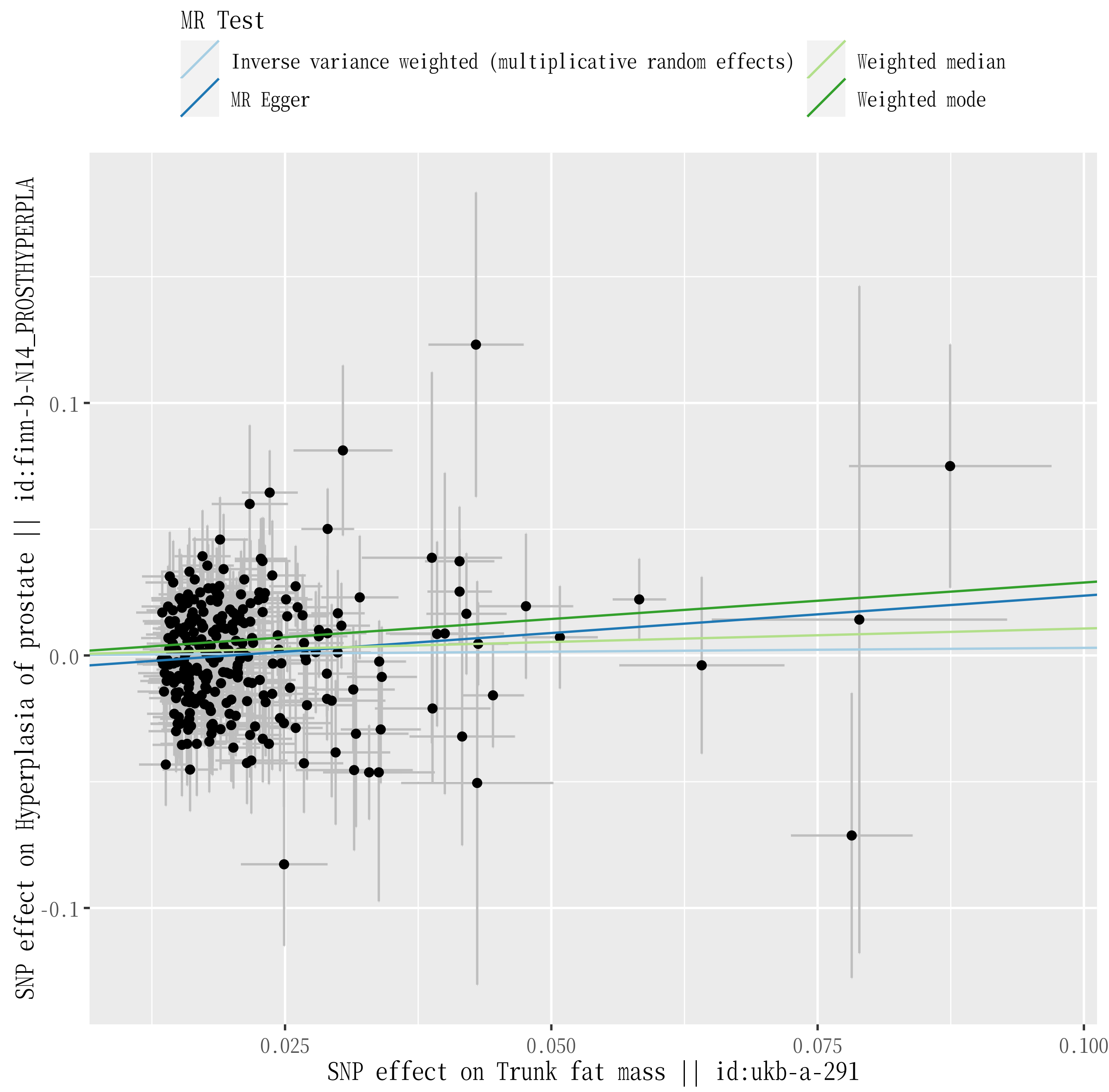
MR Test
Inverse variance weighted (multiplicative random effects)
MR Egger
Weighted median
Weighted mode
SNP effect on Hyperplasia of prostate || id:finn-b-N14_PROSTHYPERPLA
0.1
0.0
-0.1
0.025
0.050
0.075
0.100
SNP effect on Trunk fat mass || id:ukb-a-291

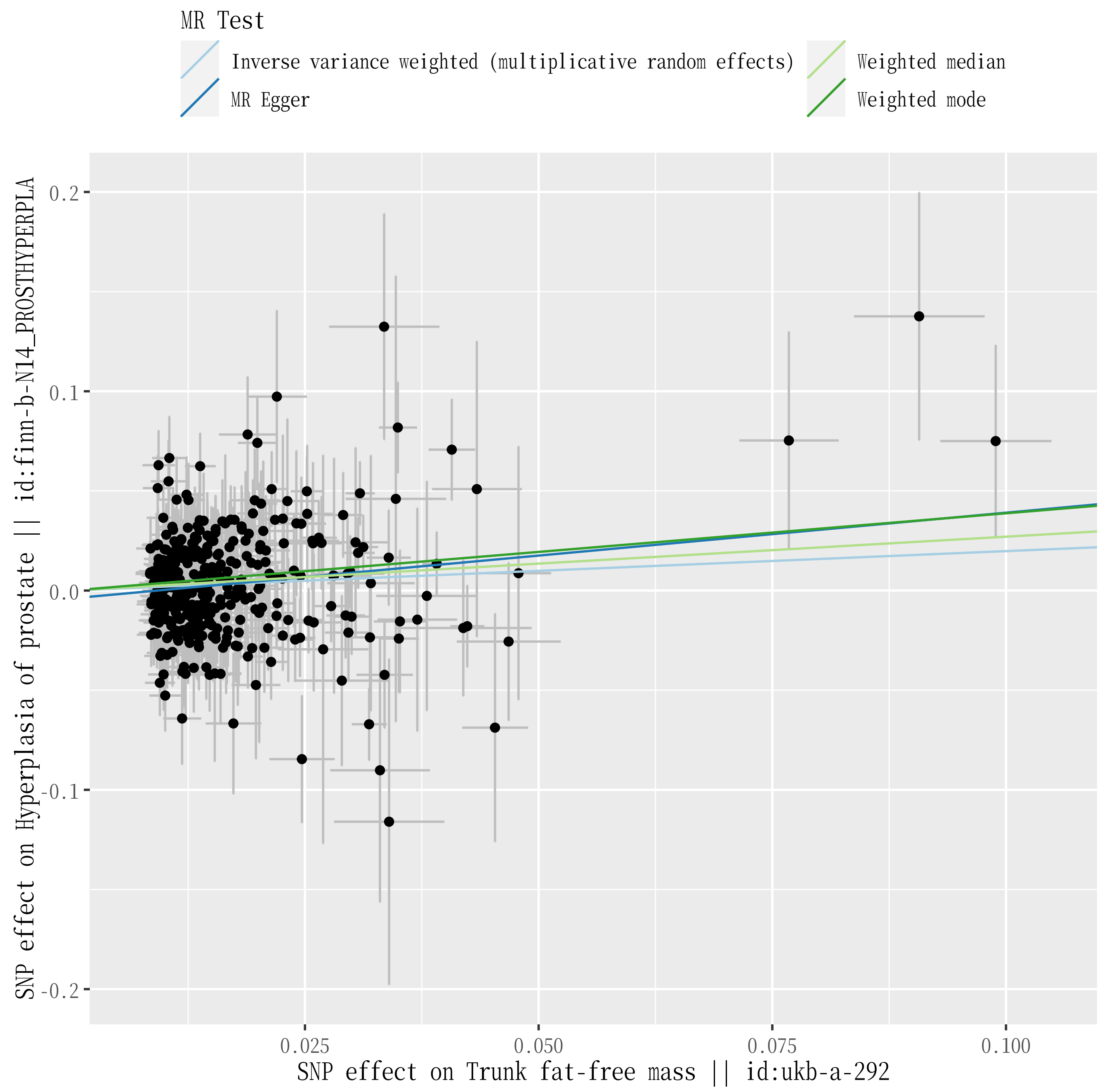
MR Test
Inverse variance weighted (multiplicative random effects)
MR Egger
Weighted median
Weighted mode
SNP effect on Hyperplasia of prostate || id:finn-b-N14_PROSTHYPERPLA
0.2
0.1
0.0
-0.1
-0.2
0.025
0.050
0.075
0.100
SNP effect on Trunk fat-free mass || id:ukb-a-292

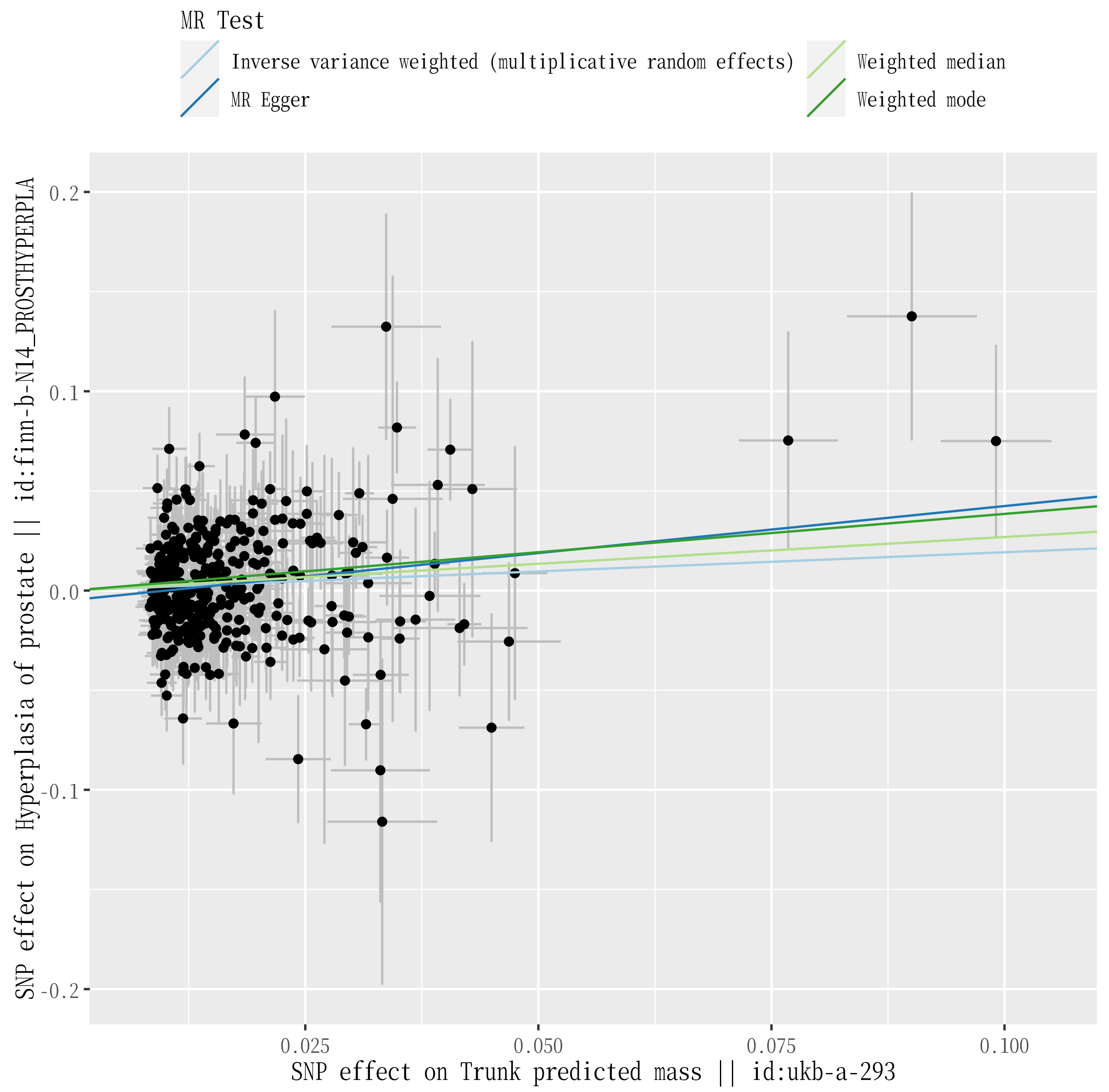

MR Test
Inverse variance weighted (multiplicative random effects)
MR Egger
Weighted median
Weighted mode
0.2
0.1
0.0
-0.1
-0.2
SNP effect on Hyperplasia of prostate || id:finn-b-N14_PROSTHYPERPLA
0.025
0.050
0.075
0.100
SNP effect on Trunk predicted mass || id:ukb-a-293
