# Supplementary material for: The causal relationship between sarcopenic obesity factors and benign prostate hyperplasia
Source: Front Endocrinol (Lausanne). 2023 Nov 8;14:1290639. doi: 10.3389/fendo.2023.1290639 (PMC10663947; doi:10.3389/fendo.2023.1290639)
Supplement: Supplementary Figure 1 — BMR related diagrams. [file DataSheet_1.pdf]

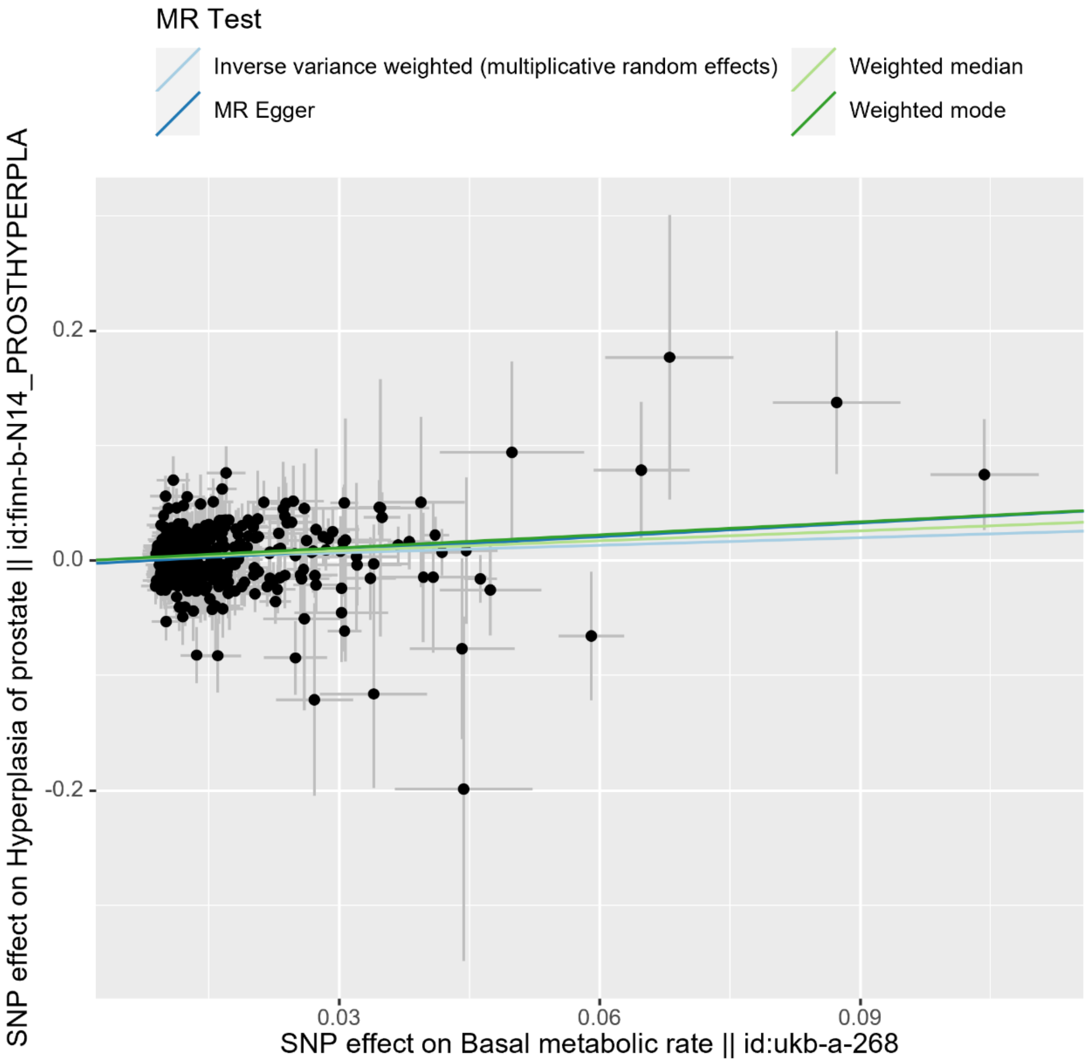
MR Test
Inverse variance weighted (multiplicative random effects)
MR Egger
Weighted median
Weighted mode
0.2
0.0
-0.2
0.03
0.06
0.09
SNP effect on Hyperplasia of prostate || id:finn-b-N14_PROSTHYPERPLA
SNP effect on Basal metabolic rate || id:ukb-a-268

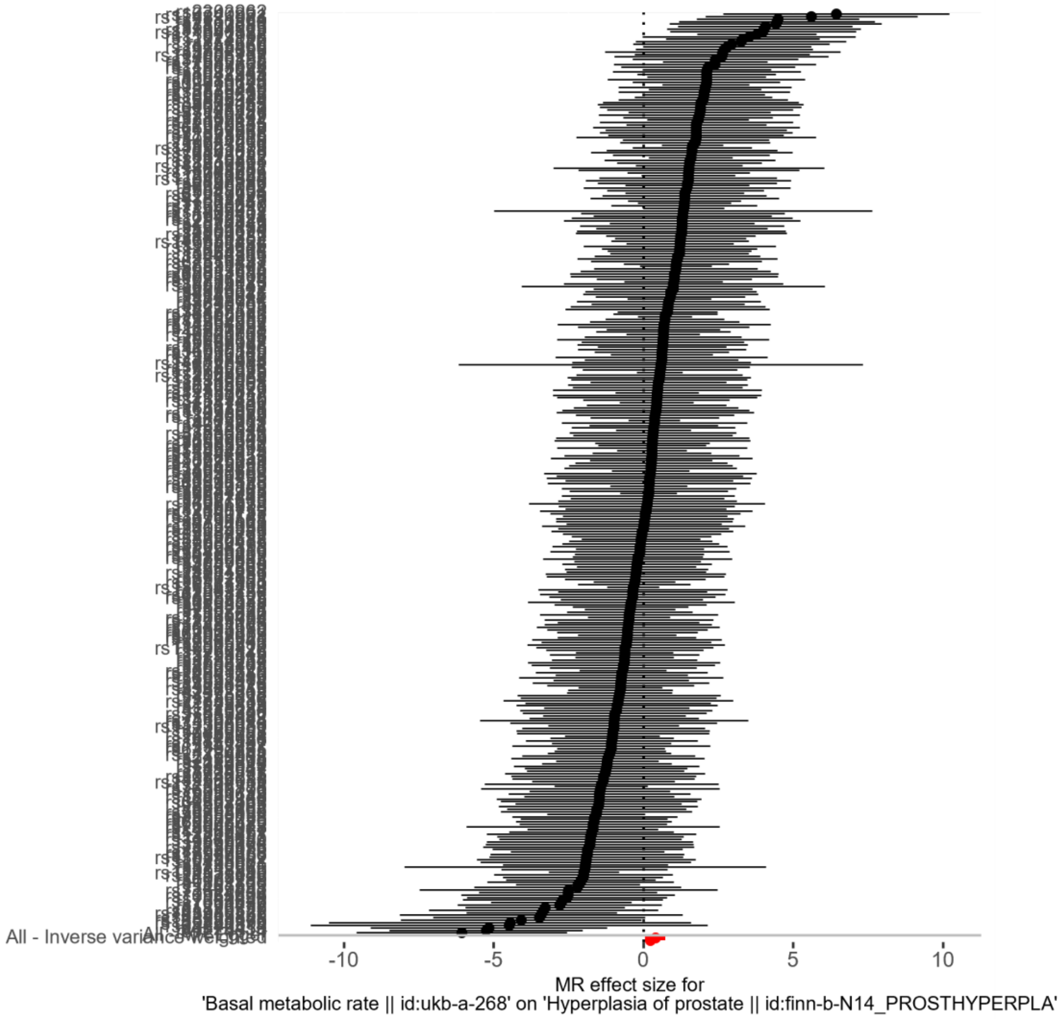

All - Inverse variance weighted
-10
-5
0
5
10
MR effect size for
'Basal metabolic rate || id:ukb-a-268' on 'Hyperplasia of prostate || id:finn-b-N14_PROSTHYPERPLA'

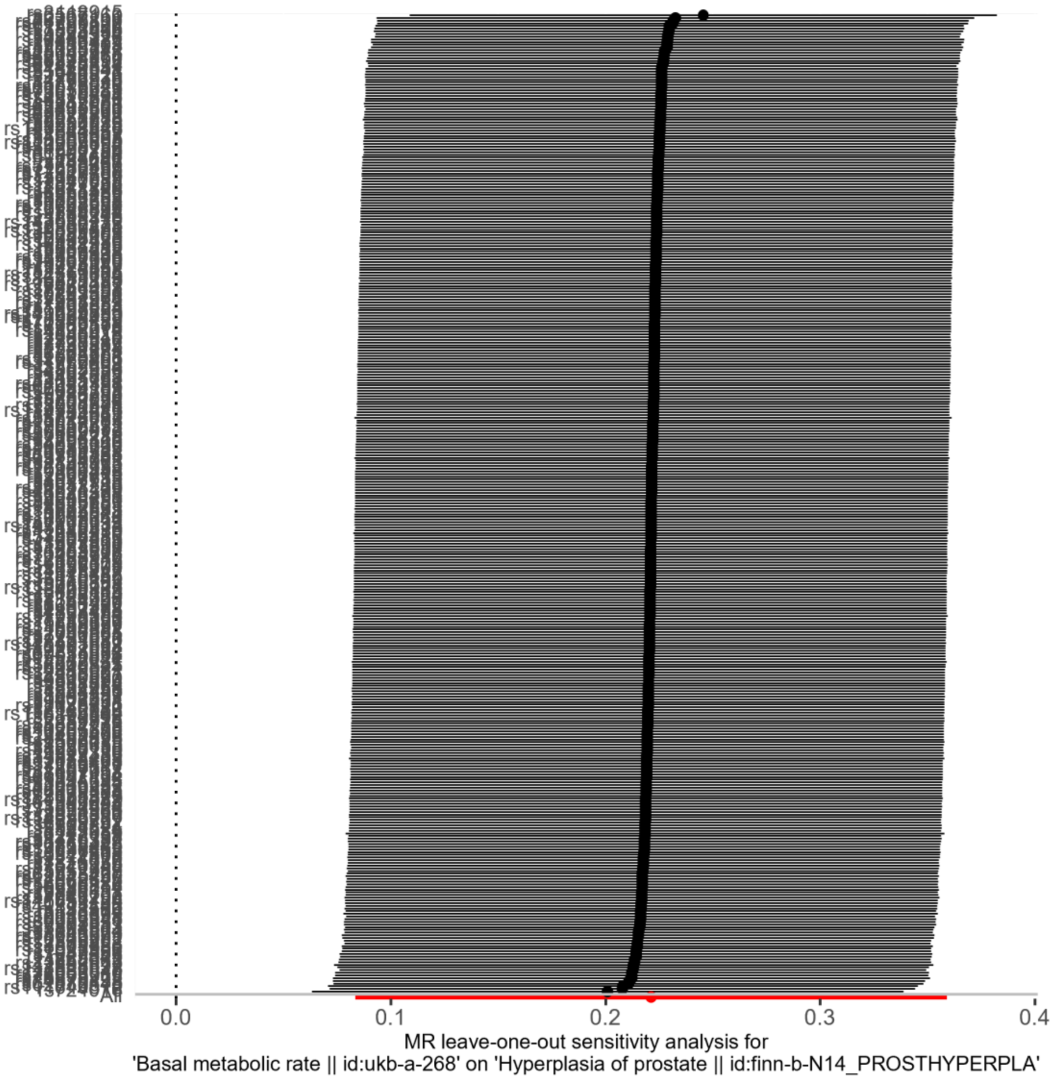
All
0.0
0.1
0.2
0.3
0.4
MR leave-one-out sensitivity analysis for
'Basal metabolic rate || id:ukb-a-268' on 'Hyperplasia of prostate || id:finn-b-N14_PROSTHYPERPLA'

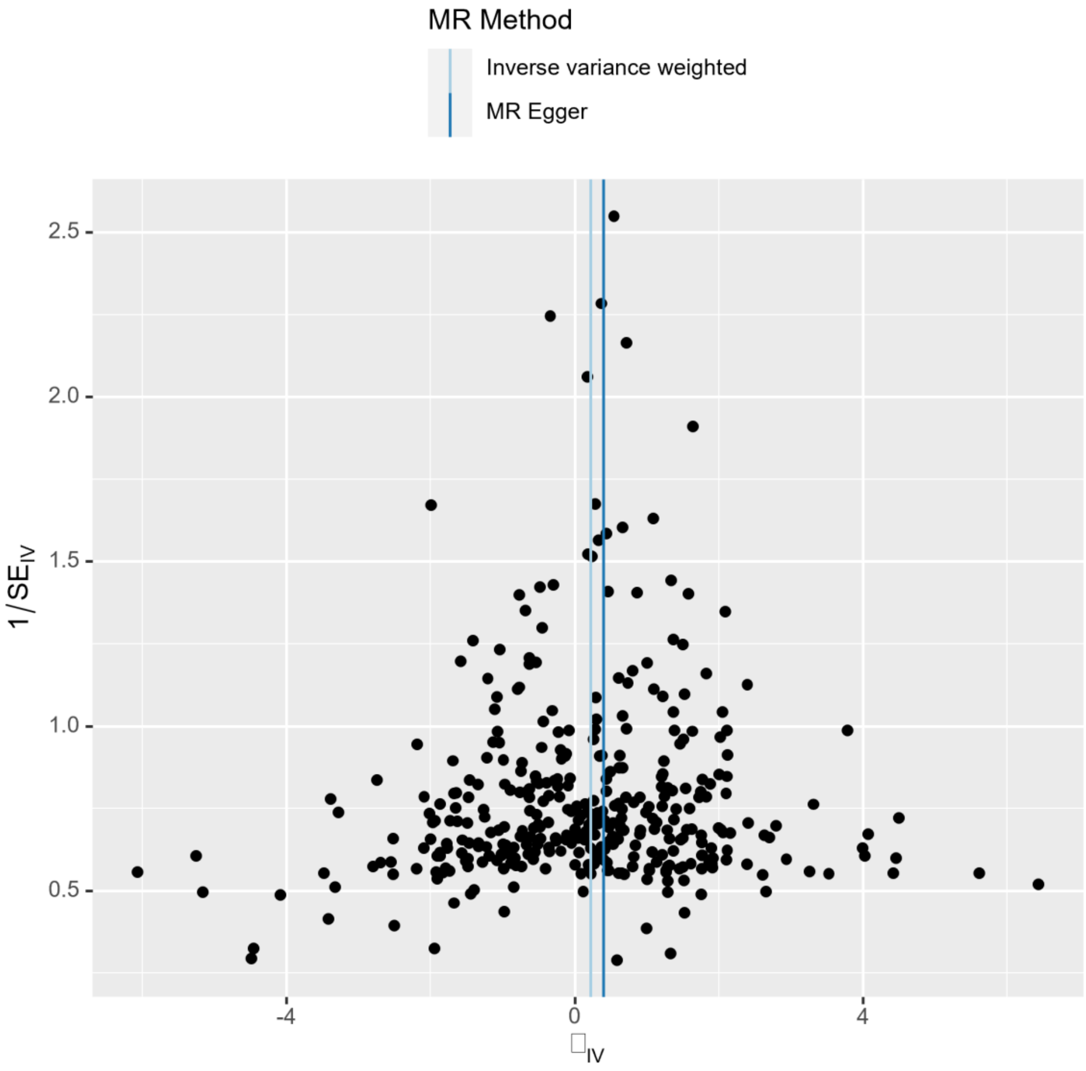

MR Method
Inverse variance weighted
MR Egger
2.5
2.0
1.5
1.0
0.5
1/SE_IV
-4
0
4
□IV
